# SETD5 regulates the OGT-catalyzed *O*-GlcNAcylation of RNA polymerase II, which is involved in the stemness of colorectal cancer cells

**DOI:** 10.1038/s41598-023-46923-1

**Published:** 2023-11-14

**Authors:** Hye In Cho, Sora Jo, Min Seong Kim, Han Byeol Kim, Xingzhe Liu, Yanhua Xuan, Jin Won Cho, Yeun Kyu Jang

**Affiliations:** 1 Department of Systems Biology, College of Life Science and Biotechnology, Yonsei University, 50 Yonsei-ro, Seodaemun-gu, Seoul, 03722 Republic of Korea; 2 Initiative for Biological Function & Systems, Yonsei University, Seoul, 03722 Republic of Korea; 3 Department of Pathology, Yanbian University College of Medicine, No.977, Gongyuan Road, Yanji, 133002 China; 4 Interdisciplinary Program of Integrated OMICS for Biomedical Science, Graduate School, Yonsei University, 50 Yonsei-ro, Seodaemun-gu, Seoul, 03722 Republic of Korea; 5 Present Address: Department of Neurology, Institute for Cell Engineering, School of Medicine, Johns Hopkins University, Baltimore, MD 21205 USA

**Keywords:** Cancer, Molecular biology

## Abstract

The dosage-dependent recruitment of RNA polymerase II (Pol II) at the promoters of genes related to neurodevelopment and stem cell maintenance is required for transcription by the fine-tuned expression of SET-domain-containing protein 5 (SETD5). Pol II *O*-GlcNAcylation by *O*-GlcNAc transferase (OGT) is critical for preinitiation complex formation and transcription cycling. SETD5 dysregulation has been linked to stem cell-like properties in some cancer types; however, the role of SETD5 in cancer cell stemness has not yet been determined. We here show that aberrant SETD5 overexpression induces stemness in colorectal cancer (CRC) cells. SETD5 overexpression causes the upregulation of PI3K-AKT pathway-related genes and cancer stem cell (CSC) markers such as CD133, Kruppel-like factor 4 (*KLF4*), and estrogen-related receptor beta (*ESRRB*), leading to the gain of stem cell-like phenotypes. Our findings also revealed a functional relationship between SETD5, OGT, and Pol II. OGT-catalyzed Pol II glycosylation depends on SETD5, and the SETD5-Pol II interaction weakens in *OGT*-depleted cells, suggesting a SETD5-OGT-Pol II interdependence. *SETD5* deficiency reduces Pol II occupancy at PI3K-AKT pathway-related genes and *CD133* promoters, suggesting a role for SETD5-mediated Pol II recruitment in gene regulation. Moreover, the *SETD5* depletion nullified the SETD5-induced stemness of CRC cells and Pol II *O*-GlcNAcylation. These findings support the hypothesis that SETD5 mediates OGT-catalyzed *O*-GlcNAcylation of RNA Pol II, which is involved in cancer cell stemness gain via CSC marker gene upregulation.

## Introduction

SETD5 is a Mixed Lineage Leukemia 5 (MLL5) homolog^1^. Most SET domain-containing proteins have enzymatic activities that can catalyze protein methylation; nonetheless, SETD5 catalytic activity remains unclear. A recombinant SETD5 protein catalyzes H3K36 and H3K9 methylation in vitro^2,3^. Other researchers have not found it in mammalian SETD5 or its orthologs in yeast and *Drosophila*^4–7^. Further, several studies have revealed that SETD5 is required for embryonic and neuronal development^8–10^. Furthermore, SETD5 has been identified as an intellectual disability (ID) gene, as mutations in *SETD5* have been found in people with ID and autism spectrum disorder (ASD)^4,11^. Furthermore, proteomic studies have revealed that SETD5 is physically and functionally associated with two complexes, polymerase-associated factor 1 (PAF1) and nuclear receptor co-repressor (NCoR)/HDAC3, suggesting its role in transcriptional repression or activation via chromatin regulation^4,5,9–12^. *SETD5* deficiency downregulates the gene involved in stem cell maintenance in murine embryonic stem cells (mESCs)^9^, implying a potential role for SETD5 in cancer stem cell (CSC) maintenance. Therefore, *SETD5* downregulation is linked to ID and ASD pathophysiology, whereas its upregulation may play a role in cancer development.

Evidence for SETD5 functional relevance in cancer has recently accumulated. SETD5 overexpression has been associated with a poor prognosis in patients with prostate cancer and non-small cell lung cancer (NSCLC)^13,14^. SETD5 promotes cell invasion in NSCLC by activating ERK signaling^13^. SETD5 also promotes cancer stem-like properties in esophageal squamous cells and breast carcinomas^15,16^. SETD5 was identified as a critical mediator of adaptive resistance to MEK1/2 inhibition-based pancreatic cancer therapy^5^.

*O*-GlcNAcylation is a reversible post-translational modification in which a single GlcNAc is added to specific serine/threonine residues of proteins using OGT. *O*-GlcNAcase (OGA) catalyzes the removal of* O*-GlcNAc. *O*-GlcNAcylation regulates the functions of proteins involved in various cellular processes, including transcription, cell signaling, and epigenetic regulation^17,18^. Proteomic studies have recently revealed that many epigenetic regulators can interact with OGT, implying that *O*-GlcNAcylation plays an important role in chromatin regulation^19,20^. *O*-GlcNAcylation regulates the functions of epigenetic regulators such as MLL5 and EZH2^17,21,22^. Previous studies have revealed that SETD5 can form complexes with OGT^4,9^, nevertheless, no evidence for the *O*-GlcNAcylation of SETD5 has been found, and its role in SETD5 function regulation remains unknown.

As previously stated, SETD5-mediated chromatin regulation involves neurodevelopment and stem cell maintenance by interacting with two complexes, Paf1C and HDAC3/NCoR. The following is a summary of previous key findings on the functional relationship between SETD5, OGT, and RNA Polymerase II (Pol II): (i) Through Paf1-mediated Pol II recruitment, SETD5 maintains proper levels of Pol II at HDAC3-occupied transcriptional start sites (TSS) of neuro-specific genes^4^. (ii) Stem cell maintenance genes are downregulated in *SETD5* null mESCs, implying that SETD5 may be a positive regulator of stem cell identity^9^. (iii) SETD5 forms a complex with OGT and RNA Pol II subunits^4,9,10^. (iv) *O*-GlcNAcylation of the RNA Pol II C-terminal domain (CTD; consensus heptad repeat Y^1^S^2^P^3^T^4^S^5^P^6^S^7^) is essential for its occupancy at the transcriptional start site (TSS)^23^. (v) The interaction of phosphorylation and *O*-GlcNAcylation of RNA Pol II CTD is required for cycling between preinitiation complex (PIC) formation and transcriptional initiation/elongation^17,24–27^. There is no clear evidence that OGT can be targeted for *O*-GlcNAcylation of RNA Pol II, resulting in transcriptional activation of CSC marker genes in SETD5-overexpressed cancer cells. In this present study, we investigated the association between SETD5 overexpression and the expression of CSC markers and PI3K-AKT pathway-related genes to determine whether SETD5 plays a role in CSC function in CRC cells. Furthermore, we tested the hypothesis that SETD5 could be a potential mediator for *O*-GlcNAcylation of RNA Pol II via OGT recruitment, resulting in transcriptional activation of target genes.

## Results

### Expression of SETD5 is correlated with CD133 expression in vivo and is associated with the survival of colon adenocarcinoma patients

**Figure 1:**
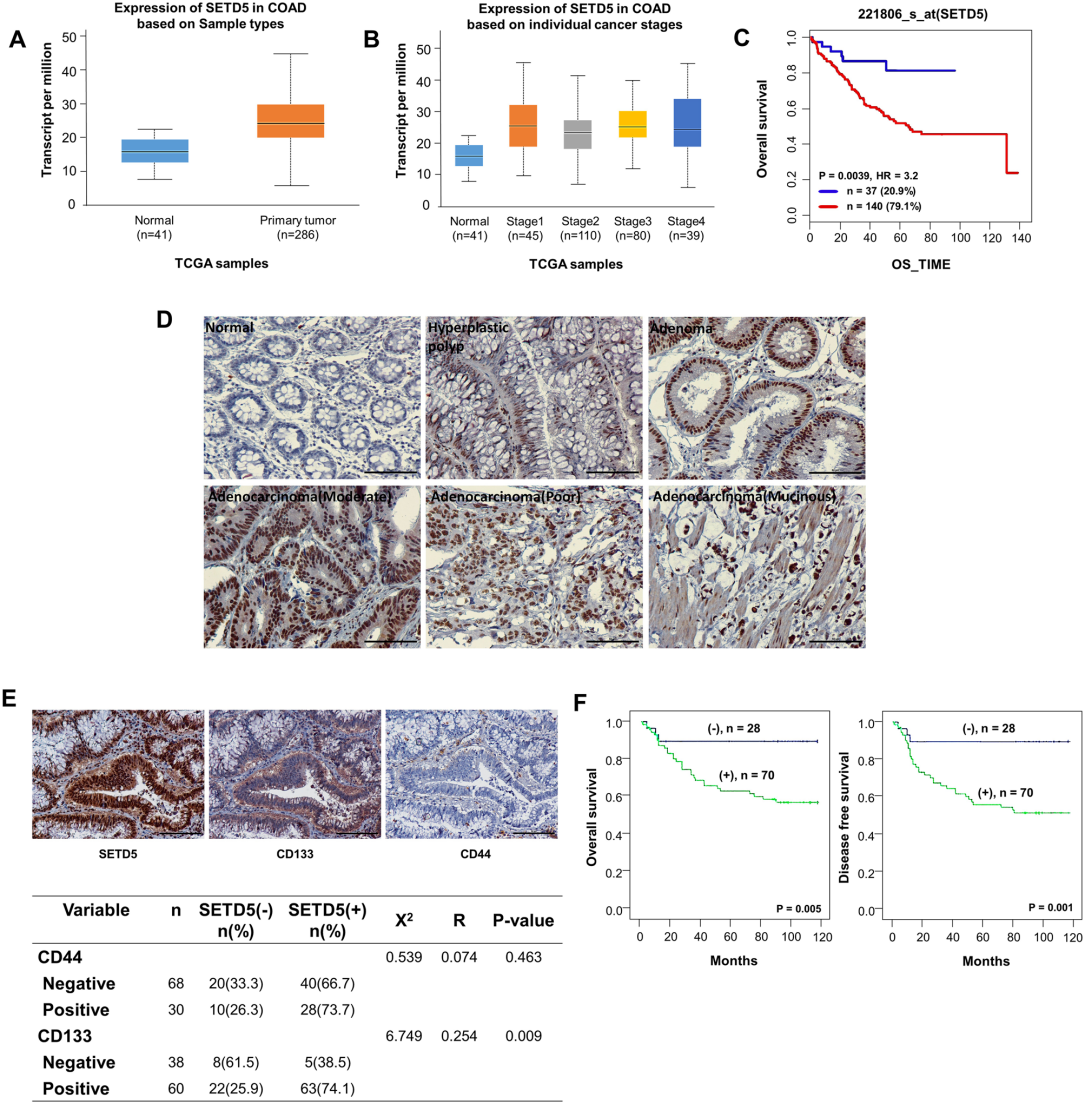
The SETD5 expression is significantly associated with the survival of colon adenocarcinoma patients, as consistent with the positive correlation with CD133, a CSC marker. (**A**–**C**) The high expression of SETD5 in CRC tumor tissues and the dosage-dependent poor survival rates were revealed by UALCAN cancer dataset. Expression of SETD5 in colon adenocarcinoma tissues (COAD) based on normal colon tissues and primary tumor tissues (**A**) and individual cancer stages (**B**). Overall survival of CRC patients with low SETD5 and high SETD5 (**C**). (**D**,**E**) Expression and distribution of SETD5 in colonic lesions (normal mucosa, hyperplastic polyp, adenoma, well differentiated, poorly differentiated, and mucinous adenocarcinoma) detected by immunohistochemistry (original magnification ×100; Scale bar: 50 μm) (**D**). Immunohistochemical staining of colon carcinoma with SETD5, CD133, and CD44 antibodies at serial section (original magnification ×200; Scale bar: 50 μm) (upper panel) (**E**). Association between SETD5 protein and cancer stem cell markers, CD133 (*p* < 0.009) and CD44 (*p* = 0.463) in colorectal cancer tissues (bottom panel) (**E**). (**F**) Kaplan–Meier analyses of overall (OS) and disease-free survival (DFS) curves for SETD5 expression in colon cancer patients. High expression of SETD5 was associated with poor OS (*p* = 0.005) and DFS (*p* = 0.001).

SETD5 is overexpressed and dysregulated in various types of cancer, including prostate cancer^13,14,28^. Consistent with these findings, the UALCAN cancer dataset showed that the transcriptional expression of *SETD5* was significantly upregulated in colon adenocarcinoma (COAD) tissues (Fig. 1A) and was overexpressed at various clinical stages compared to normal colon tissues (Fig. 1B). Patients with CRC with high SETD5 levels had lower overall survival than patients with CRC with low SETD5 levels (Fig. 1C). *SETD5* expression was mainly localized in the nuclei of hyperplastic polyps, adenomas, and colon adenocarcinoma tissues, based on immunohistochemical analyses. However, SETD5 expression did not differ significantly between lesions (Fig. 1D and Supplementary Table S4). Subsequently, we compared the clinicopathological characteristics of CRC tissues with SETD5 expression. *SETD5* expression in colon adenocarcinoma was linked to the distant metastasis (*p* = 0.027) and radiotherapy (*p* = 0.023) of the patients. In contrast, no significant correlation was found between SETD5 expression and age, sex, tumor grade, tumor location, tumor size, differentiation, T stage, lymph node metastasis, clinical stage, or chemotherapy (Supplementary Table S5).

We hypothesized that SETD5 is involved in regulating CSC maintenance because CSCs are frequently a major cause of cancer recurrence and metastasis^29^. The two most critical markers related to colorectal CSCs are CD44 and CD133, and they play significant roles in diagnosis, treatment, and prognosis^30^. To determine whether SETD5 plays a role in CSC function, we investigated the association between *SETD5* and the CSC markers CD133 and CD44 in colon adenocarcinoma. *SETD5* expression was moderately correlated with CD133 expression in colon adenocarcinoma (*p* < 0.009). However, there was no significant association between SETD5 and CD44 expression in colon adenocarcinoma (Fig. 1E). In addition, SETD5 and CD133 were found to be co-localized in adenocarcinoma tissue serial tissue (Fig. 1E). Moreover, OS and DFS analyses revealed that SETD5 expression was significantly associated with poor OS (*p* = 0.005) and DFS (*p* = 0.001) in patients with colon adenocarcinoma (Fig. 1F and Supplementary Tables S6 and S7). Thus, these findings suggest that SETD5 correlates with CD133 expression in vivo and with the survival rates of patients with colon adenocarcinoma. These findings suggest that SETD5 could be used as a biomarker and therapeutic target in colorectal cancer due to its impact on CSC function.

### SETD5 is required to maintain the stem cell-like phenotype of colorectal cancer cells

**Figure 2:**
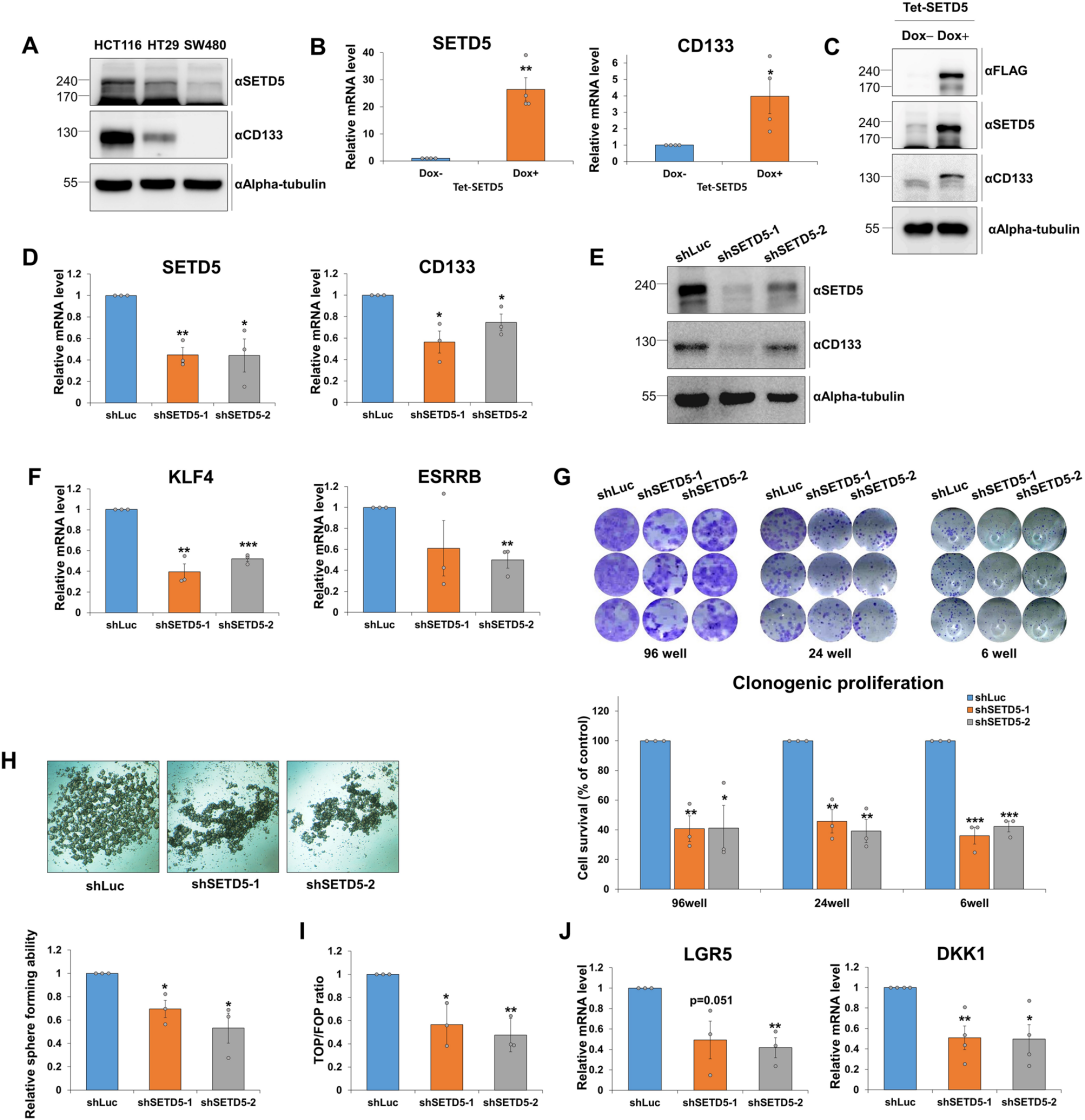
The aberrant overexpression of SETD5 causes the stem cell-like phenotypes of colorectal cancer cells. (**A**) The SETD5 and CD133 protein expression levels in CRC cell lines (HCT116, HT29, and SW480) were examined by western blot. Alpha-tubulin was used as a loading control. (**B**,**C**) The ectopic overexpression of SETD5 causes the aberrant induction of CD133 expression. The expression of SETD5 and CD133 in SETD5-overexpressing SW480 cells was determined by qRT-PCR (**B**) and western blot (**C**). The SETD5 overexpression was induced by 100 ng/ml of doxycycline (Dox)-treatment on Tet-inducible SETD5 cells for 24 h. The mRNA level was normalized to that of GAPDH. The expression level of the control cells was set as 1 (n = 3 independent experiments). Symbols used: Dox(−), mock treatment without doxycycline; Dox(+), treated with doxycycline. (**D**,**E**) The expression of CD133, a CSC marker, was significantly decreased by the *SETD5* depletion in the HCT116 cells overexpressing the SETD5. The expression of SETD5 and CD133 in *SETD5-*knockdown (KD) HCT116 cells was confirmed by qRT-PCR (**D**) and western blot (**E**). n = 3 independent experiments. (**F**) The mRNA expression of CSC markers (*KLF4, ESRRB*) in *SETD5-*KD HCT116 cells was confirmed by qRT-PCR. n = 3 independent experiments. (**G**) Clonogenic proliferation was investigated in *SETD5*-depleted HCT116 cells. The representative images of stained cells were taken from plates with different well numbers (upper panel). The proportion of the alive cells was quantified by measurement of the colony-containing areas from the upper image (bottom panel). Cell survival of the control KD cells was set as 100%. n = 3 independent experiments. (**H**) Tumor-initiating ability of CRC cells was reduced by the *SETD5* depletion. Tumorsphere-forming ability of *SETD5*-depleted HCT116 cells was monitored. Phase contrast images of tumorspheres derived from *SETD5-KD* cells and control KD cells were shown in the upper panel. Quantification of the sphere-forming cells in *SETD5* KD cells was shown (bottom panel). The viability of the control sphere cells was set as 1. n = 3 independent experiments. (**I**) The Wnt/ß-catenin-related transcriptional activity was significantly reduced by the *SETD5* deficiency. The Wnt/ß-catenin activity was measured by TOP/FOP luciferase assay in the *SETD5* KD cells and their control counterpart. The luciferase activity of TOP flash was normalized by FOP flash. The luciferase activity of the control KD cells was set as 1. (**J**) The reduced mRNA expression of Wnt/ß-catenin target genes (*LGR5* and *DKK1*) in the *SETD5*-depleted HCT116 cells was confirmed by qRT-PCR. The mRNA levels were normalized to those of GAPDH. The expression level of the control KD cells was set as 1 (n = 3). Data are represented as mean ± SEM of triplicate measurements; *p < 0.05, **p < 0.01, and ***p < 0.001.

We further investigated the effects of SETD5 on the stemness of CRC cells. Before constructing SETD5 overexpression or knockdown cell lines, we determined SETD5 expression levels in several CRC cell lines, such as HCT116, HT29, and SW480. SETD5 was most highly expressed in HCT116 cells among these cell lines, which also had the highest CD133 expression; however, both SETD5 and CD133 expression levels were the lowest in SW480 cells (Fig. 2A). Subsequently, we used SW480 cells to create doxycycline (Dox)-inducible conditional SETD5 overexpression cell lines and HCT116 cells to create *SETD5*-depleted cell lines. SETD5 overexpression upregulated CD133 mRNA (Fig. 2B) and protein expression (Fig. 2C). Conversely, the knockdown of *SETD5* resulted in the downregulation of CD133 mRNA (Fig. 2D) and protein expression (Fig. 2E). In addition, it aided in the suppression of transcription of other CSC marker genes, such as *KLF4* and *ESRRB* (Fig. 2F). We performed clonogenic proliferation and tumorsphere formation assays to determine how SETD5 affected CRC cell self-renewal and tumor-initiating ability. Therefore, cell survival rates and sphere-forming ability were significantly lower in *SETD5*-depleted cells compared to control knockdown cells, implying that SETD5 may play a role in CRC self-renewal and tumor-initiating abilities (Fig. 2G,H).

Aberrant Wnt/β-catenin signaling is a major contributor to the maintenance and progression of colorectal CSC and CRC cells^31–34^. Therefore, TOP/FOP assay and qRT-PCR were used to see if *SETD5* deficiency affects transcriptional activity associated with Wnt/β-catenin signaling in CRC cells. Using the TOP/FOP assay, we found that *SETD5* depletion reduced the transcriptional activity associated with Wnt/β-catenin signaling (Fig. 2I) and mRNA expression of Wnt/β-catenin target genes, such as leucine-rich repeat-containing G-protein coupled receptor 5 (*LGR5*) and Dickkopf-related protein 1 (*DKK1*) (Fig. 2J). Overall, our findings support the notion that SETD5 regulates the stem cell-like phenotypes of CRC cells.

### SETD5 is *O*-GlcNAcylated by OGT, and its C-terminal region is essential for its function

Next, we aimed to determine how SETD5 affects the stem cell-like phenotype of CRC. SETD5 has a SET domain that may contribute to methyltransferase activity; however, its enzymatic activity remains debatable^2,4,9^. Therefore, we used a histone methylation assay with purified histone proteins to determine whether the methyltransferase activity of SETD5 was involved in regulating the expression of CSC marker genes in CRC. H3 and H4 methylation levels in Dox-inducible SETD5 overexpressed SW480 cells were comparable to those in untreated SW480 cells (Supplementary Fig. S1A). To confirm that its methyltransferase activity does not interfere with SETD5 function, we generated a dSET mutant lacking the SET domain and analyzed CD133 expression using western blotting and qRT-PCR. Our results showed that CD133 expression in SW480 cells expressing the dSET mutant was not significantly different from that in SW480 cells expressing WT SETD5 (Supplementary Fig. S1B,C). Therefore, these findings indicate that SETD5 does not function as a methyltransferase in colorectal cancer cells and is not required for *CD133* gene regulation.

**Figure 3:**
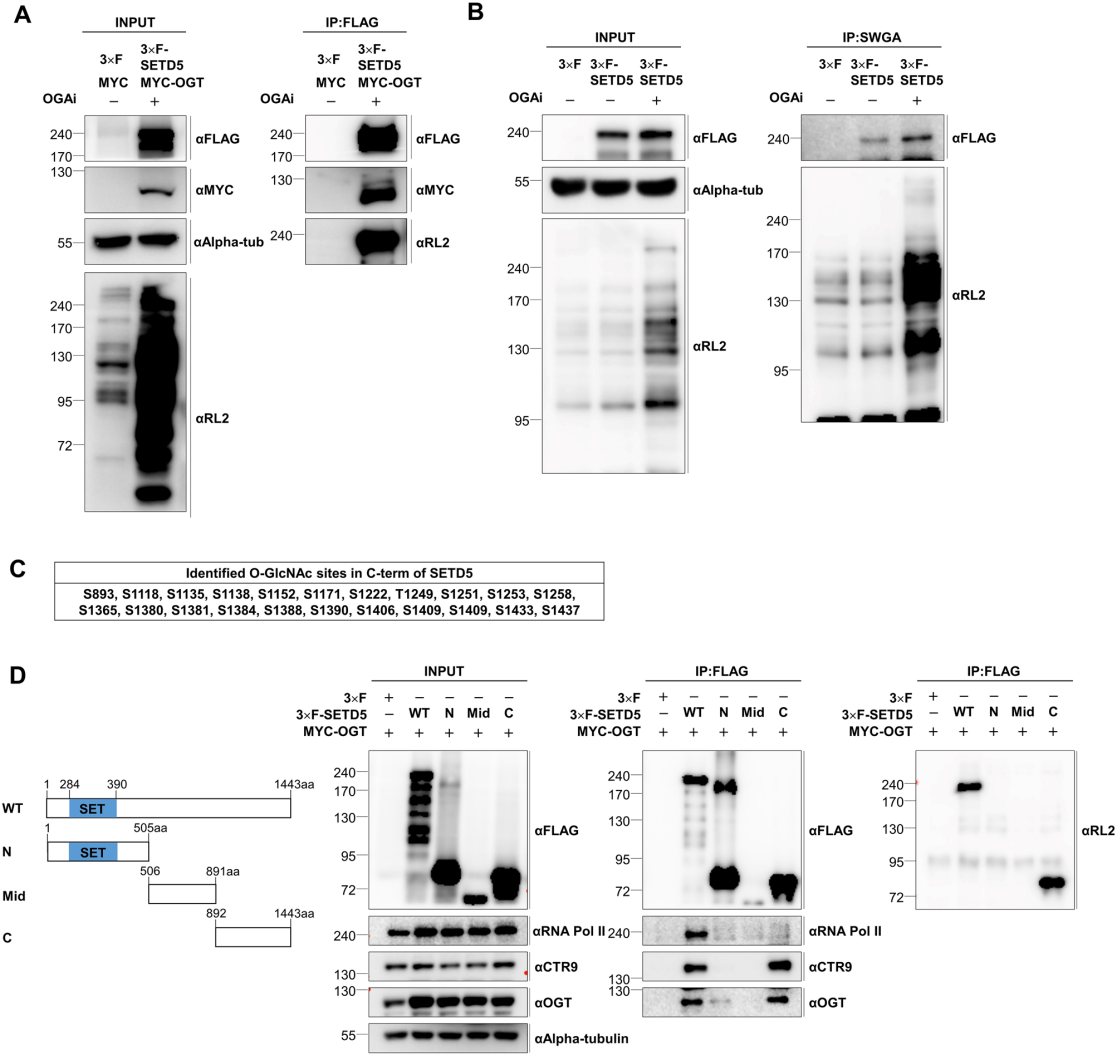
The C-terminal region of SETD5 is the main part of OGT-catalyzed *O*-glycosylation, which is essential for the functional relationship between SETD5, OGT, and RNA Pol II. (**A**) SETD5 interacted with OGT and was glycosylated. The interaction of SETD5 with OGT and the *O*-GlcNAcylation of SETD5 were analyzed by immunoprecipitation (IP) assay. 3× F-SETD5 and MYC-OGT were co-transfected into 293 T cells, followed by treatment of 100 nM OGA inhibitor (Thiamet-G; OGAi) for 24 h. FLAG-tagged SETD5 was immunoprecipitated by FLAG-affinity gel, and the immunoprecipitates were subjected to western blot. Symbols used: 3× F, 3× FLAG-tagged empty vector; MYC, MYC-tagged empty vector; 3× F-SETD5, 3× FLAG-tagged-SETD5 plasmid; MYC-OGT, MYC-tagged OGT plasmid; αAlpha-tub, Alpha-tubulin antibody; αRL2, *O*-GlcNAc-specific antibody. (**B**) SETD5 is *O*-GlcNAcylated by OGT. The SETD5 *O*-GlcNAcylation was determined by in vivo glycosylation assay using sWGA beads-based IP. Following immunoprecipitation, SETD5 was detected using a FLAG antibody as a primary antibody. The OGAi of 100 nM was treated for maximizing *O*-GlcNAcylation of SETD5 prior to the immunoprecipitation of SETD5. Symbols used: sWGA, IP using succinylated wheat germ agglutinin beads for the enrichment of *O*-GlcNAcylated proteins. (**C**,**D**) The *O*-GlcNAcylation site(s) of SETD5 were identified by LC–MS/MS analysis. The FLAG-tagged SETD5 was purified from 293 T cells and subjected to LC–MS/MS analysis to identify the potential *O*-GlcNAcylation site(s) (**C**). The *O*-GlcNAc sites were mostly found in the C-terminal region of SETD5 (**D**). (**E**) The schematic diagram for full-length SETD5 (WT, wild-type) and its three truncated forms (N, N-terminal part; Mid, middle part; C, C-terminal part) (left panel). Immunoprecipitations revealed the interaction between SETD5, OGT, RNA Pol II, and CTR9 (right panel). The *SETD5* deletion mutants were co-transfected with MYC-tagged OGT into 293 T cells, then immunoprecipitated by FLAG affinity gel, and following western blot analysis.

Previous studies have revealed that SETD5 interacts with OGT^4,9^, suggesting that SETD5 may be *O*-GlcNAcylated by OGT. Hence, we performed an immunoprecipitation assay to assess whether OGT regulates SETD5 via *O*-GlcNAc modification. Our findings revealed that SETD5 interacted with OGT and was *O*-GlcNAcylated by OGT (Fig. 3A). Conversely, our immunoprecipitation using sWGA beads revealed that SETD5 is glycosylated. Furthermore, this treatment with an OGA inhibitor (OGAi) increases this glycosylation, implying that the glycosylation of SETD5 was OGT-mediated *O*-GlcNAc (Fig. 3B). The *O*-GlcNAcylation sites within SETD5 were mapped using mass spectrometry. Our finding revealed 22 potential *O*-GlcNAcylation sites, most of which were found in the SETD5 C-terminus (Fig. 3C, and Supplementary Fig. S2A), suggesting that the C-terminus may play an important role in SETD5 function.

We used three *SETD5* deletion mutants (N-terminal, middle, and C-terminal) to assess the importance of the C-terminal region of SETD5 in mediating its function. Our findings revealed that *O*-GlcNAcylation of SETD5 was concentrated in the C-terminus, which is consistent with the mass spectrometry data (Fig. 3D). Moreover, the *SETD5* deletion mutant containing a C-terminal region could interact with the well-known binding partners CTR9 and OGT. However, not with N-terminus and Middle part mutants (Fig. 3D). Thus, our findings suggest that the C-terminus of SETD5 is important for *O*-GlcNAcylation and protein–protein interactions.

We used site-directed mutagenesis to create glycosylation-defective mutants to determine the critical *O*-GlcNAcylation site(s) of SETD5. Among the 22 sites identified by Mass-spec, six threonine(T)/serine(S) residues (T735, S817, S874, T1249, S1380, T1388) were selected as potential target sites with higher detection frequency in Mass-spec data, and subjected to mutagenesis to replace the residue with alanine (A). Further, when the glycosylation levels of the double (S1380A/T1388A) and triple (T1249A/S1380A/T1388A) mutants were compared to those of WT-SETD5, none showed significant defects in glycosylation (Supplementary Fig. S2B,C). Thus, we speculated that *O*-GlcNAc modification of SETD5 may occur at most of the potential sites within the C-terminal region, as identified by Mass-spec. We tested whether the C-terminal deletion mutants (1258* and 1368*) were glycosylation-defective to map the domain responsible for the glycosylation of SETD5. The 1258* mutant is a deletion mutant that is unable to bind with its partners, whereas the 1368* mutant is a frameshift mutation found in patients with ID^4^. However, our data showed that the glycosylation levels in SETD5 were comparable to those in WT-SETD5 (Supplementary Fig. S2D), suggesting that the domain (S893–S1258) is required for SETD5 glycosylation.

### SETD5 mediates *O*-GlcNAcylation of RNA polymerase II

To determine the role of *O*-GlcNAcylation in SETD5 function, we first examined whether *O*-GlcNAcylation influenced SETD5 protein stability under conditions of *O*-GlcNAcylation enrichment or suppression. To put this hypothesis to the test, *O*-GlcNAcylation was either enriched by ectopic expression of MYC-tagged OGT or OGAi treatment or suppressed by treatment with small-interfering RNA targeting OGT (siOGT). Our findings revealed that enriched or suppressed *O*-GlcNAcylation had no effect on SETD5 protein stability (Supplementary Fig. S3A). Further, since SETD5 interacts with the PAF1 complex (CTR9, PAF1, and CDC73) and NcoR1 complex (NCOR1, TBL1X, and HDAC3), we investigated whether *O*-GlcNAcylation of SETD5 affects its interaction with its partner proteins, such as CTR9 and TBL1X^4,9,10^. Furthermore, the IP assay reveals that different levels of *O*-GlcNAcylation had a minor effect on the interaction between SETD5 and its binding partners (Supplementary Fig. S3B,C). The SETD5-PAF1 complex is tightly associated with RNA polymerase II during transcription, and RNA Pol II is a well-known substrate for OGT^9,35,36^. Therefore, we used IP assays to determine whether SETD5 can interact with RNA Pol II. Our findings revealed that RNA Pol II only binds to full-length SETD5, whereas CTR9 and OGT can form a complex with both the C-terminal region and full-length SETD5 (Fig. 3D). Hence, these findings suggest that SETD5 directly or indirectly interacts with RNA Pol II.

**Figure 4:**
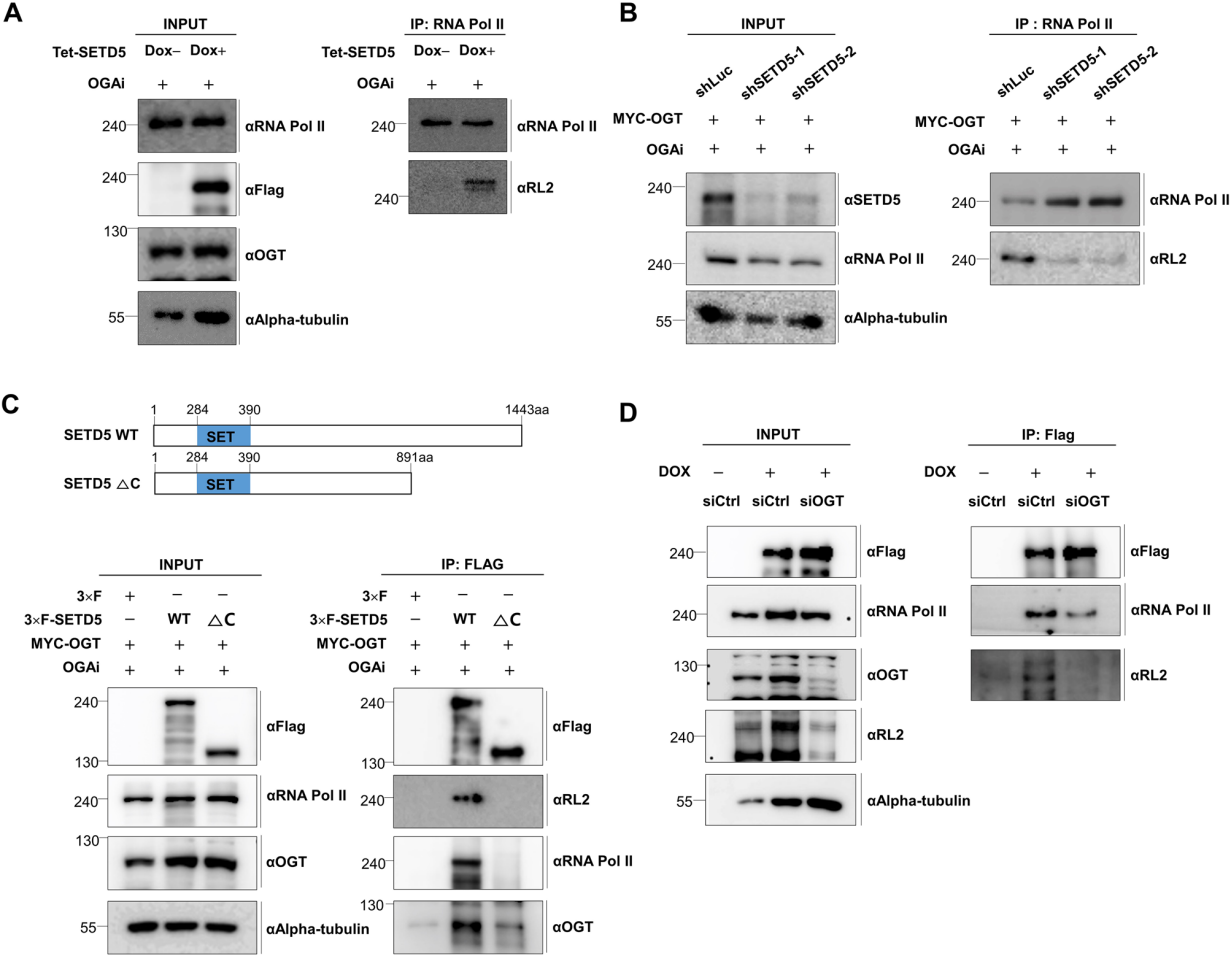
SETD5 mediates the OGT-catalyzed *O*-GlcNAcylation of RNA Pol II. (**A**) The *SETD5* overexpression resulted in the promotion of RNA Pol II *O*-GlcNAcylation. The *O*-GlcNAcylation of RNA Pol II in the SETD5-overexpressed cells was determined by immunoprecipitation assay using an RNA Pol II antibody and the subsequent western blot with an *O*-GlcNAc-specific antibody. The *O*-GlcNAcylation in the immunoprecipitates was monitored by western blot using RL2, an *O*-GlcNAc-specific antibody. The SETD5 overexpression was induced by 100 ng/ml of Dox treatment on Tet-inducible SETD5-based SW480 cells, and 100 nM of OGA inhibitor (OGAi) for 24 h. Symbols used: Dox(−), mock treatment without doxycycline; Dox(+), treated with doxycycline. (**B**) The *SETD5* depletion resulted in the reduction of RNA Pol II *O*-GlcNAcylation. The RNA Pol II *O*-GlcNAcylation in the *SETD5-*depleted cells was determined as mentioned in (**A**) of this figure. MYC-tagged OGT was transfected into the *SETD5*-depleted HCT116 cells and then treated with 100 nM of OGAi for 24 h. (**C**) The schematic diagram showing the full-length SETD5 (WT) and its SETD5 deletion mutant (ΔC) lacking the C-terminal region containing all potential *O*-GlcNAcylation sites of SETD5 (upper panel). The SETD5 *O*-GlcNAcylation and the interaction of SETD5 with OGT or RNA Pol II were analyzed by IP assay (bottom panel). MYC-tagged OGT was transfected into the *SETD5*-depleted HCT116 cells and then treated with 100 nM of OGAi for 24 h. (**D**) The SETD5-RNA Pol II interaction weakened when *OGT* was depleted in the SETD5-overexpressed cells. The siRNAs targeting the OGT mRNA (siOGT) were transfected into Tet-inducible SETD5-based SW480 cells, and 100 ng/ml of doxycycline was treated for inducing the SETD5 overexpression. Symbols used: siCtrl, control siRNA.

By recruiting RNA Pol II to gene promoters, *O*-GlcNAcylation of RNA Pol II is essential for transcription initiation^23,26^. We tested whether SETD5 influences the *O*-GlcNAcylation level of RNA Pol II using in vivo glycosylation assay because SETD5 interacts with both OGT and RNA Pol II. An *O*-GlcNAcylation assay was performed under OGAi-treated conditions to induce *O*-GlcNAcylation of SETD5. Our findings revealed that SETD5 overexpression upregulated *O*-GlcNAcylation of RNA Pol II (Fig. 4A), whereas *SETD5* depletion downregulated *O*-GlcNAcylation of RNA Pol II (Fig. 4B). These findings suggest that SETD5 regulates the *O*-GlcNAcylation of RNA Pol II. In addition, IP assay showed that endogenous SETD5 can form a complex with endogenous OGT and RNA Pol II (Supplementary Fig. S4A).

Based on our findings, we hypothesized that SETD5 *O*-GlcNAcylation is involved in the recruitment of OGT to RNA Pol II, allowing RNA Pol II* O*-GlcNAcylation. To test this hypothesis, we constructed a *SETD5* deletion mutant (ΔC) with a deletion of the C-terminal region (892–1443), deleting all 22 potential glycosylation sites. Therefore, this ΔC mutant is considered an *O*-GlcNAcylation-defective form of SETD5 (Fig. 4C) and was tested using an IP assay. Our findings revealed that the interaction between the ΔC mutant and OGT decreased relative to that of WT-SETD5. Hence, the *O*-GlcNAcylation of the ΔC mutant was abolished (Fig. 4C). Additionally, the ΔC mutant was unable to bind RNA Pol II (Fig. 4C). The C-terminal region of SETD5 itself may be required for binding to OGT or RNA polymerase II; thus, we cannot exclude the possibility that the results observed in the ΔC mutant may be due to the absence of a protein domain essential for binding with its partner, rather than to the reduced SETD5 *O*-GlcNAcylation. To resolve this, we tested whether the interaction between SETD5 and RNA Pol II was affected by *OGT*-depleted conditions in which SETD5 *O*-GlcNAcylation was blocked. Our IP assay revealed that the interaction between SETD5 and RNA Pol II was decreased in *OGT*-depleted cells compared to that in the control cells (Fig. 4D). SETD5 *O*-GlcNAcylation was also completely abolished under the same conditions (Fig. 4D), suggesting that SETD5 *O*-GlcNAcylation affected OGT-catalyzed glycosylation of RNA Pol II.

Many studies have demonstrated that OGT protein is important in protein–protein interaction^17^. Therefore, we used the OSMI-1 (OGT catalytic site inhibitor; OGTi) to clarify that the reduction in SETD5-RNA Pol II interaction revealed by siOGT treatment might be correlated with a decrease in glycosylation of SETD5 but not induced by changes in the scaffolding system due to OGT loss. Flag-tagged SETD5 was transfected with MYC-tagged OGT or MYC-tagged empty vector into HCT116 cells, which were further treated with 50 µM OSMI-1 or DMSO. Our IP assay data showed that OSMI-1 treatment reduced the global *O*-GlcNAcylation level while not affecting the OGT protein level (Supplementary Fig. S4B). The *O*-GlcNAcylation of SETD5 and the interaction between SETD5 and RNA Pol II were significantly reduced in cells treated with OSMI-1 compared to those treated with DMSO (mock treatment), suggesting the role of SETD5 *O*-GlcNAcylation in the interaction between SETD5 and RNA Pol II (Supplementary Fig. S4B). Thus, these findings suggest that SETD5 may function as a mediator of RNA Pol II *O*-GlcNAcylation.

### SETD5 regulates the PI3K-AKT pathway by recruitment of RNA polymerase II on the promoter DNA

**Figure 5:**
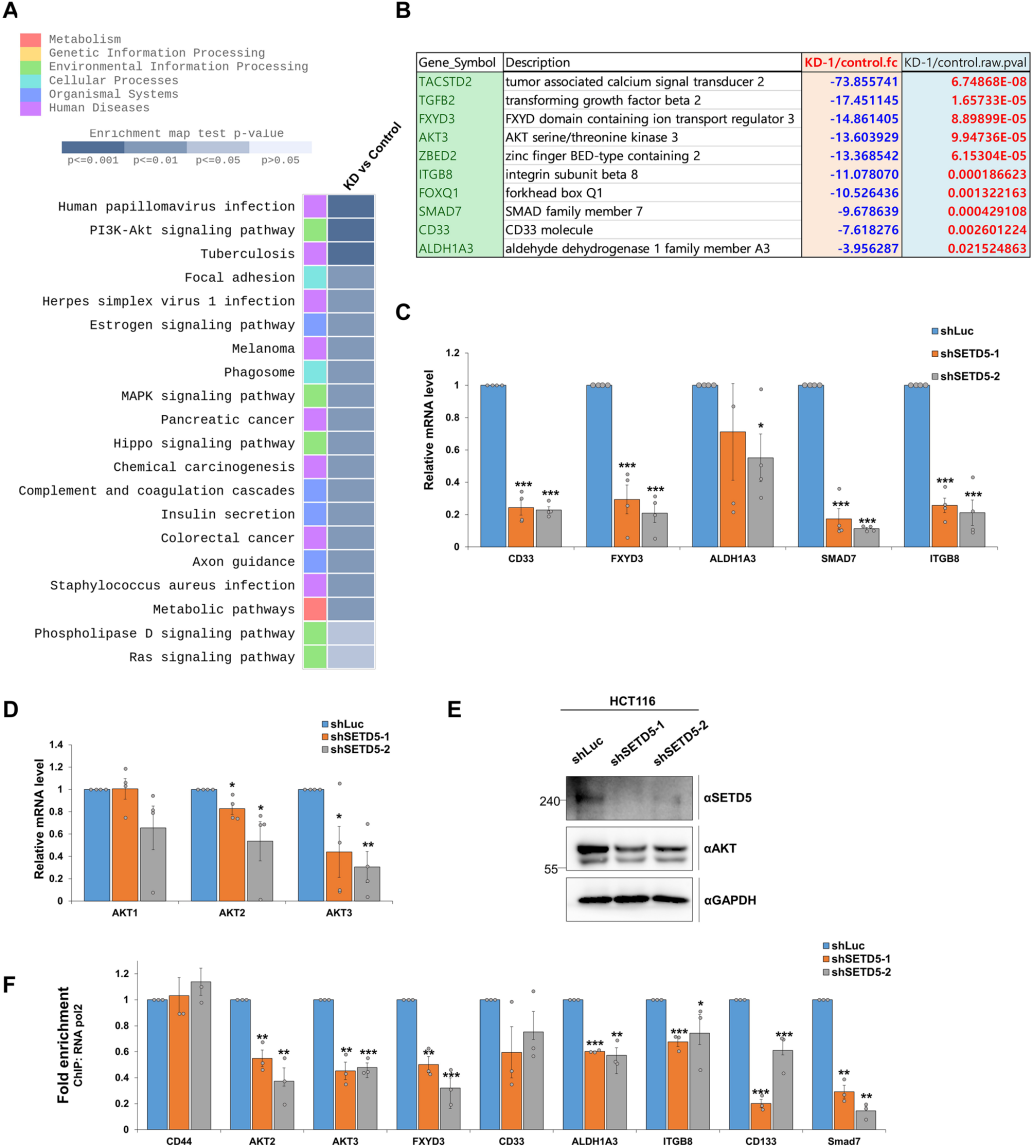
SETD5 is required for the recruitment of RNA Pol II on the promoters, which is involved in the expression of PI3K-AKT pathway-related genes and CD133, a CSC marker. (**A**,**B**) RNA-seq analyses were performed in Control and *SETD5* knockdown cells. Map of KEGG pathway (**A**). Table showing the top 10 oncogenes that are down-regulated by the *SETD5-*KD-based depletion (**B**). (**C**) The downregulated mRNA expression of target genes (*CD33, FXYD3, ALDH1A3, SMAD7,* and *ITGB8*) was confirmed in the *SETD5*-depleted HCT116 cells by qRT-PCR. The mRNA levels were normalized to that of *GAPDH*. The expression level of the control KD cells was set as 1. n = 3 independent experiments. (**D**,**E**) The mRNA expression of the *AKT* isoforms was determined by qRT-PCR (**D**). The expression level of the control KD cells was set as 1. n = 3 independent experiments. The reduced protein expression of AKT was also confirmed in *SETD5-*KD HCT116 cells (**E**). (**F**) The RNA Pol II occupancy at the promoters of PI3K-AKT-related genes and CD133 was significantly reduced by the *SETD5* depletion. ChIP assays were investigated in *SETD5*-depleted HCT116 cells using RNA Pol II antibody. CD44 is a *SETD5*-nonregulated gene and is thus used as a negative control. The expression level of the control KD cells was set as 1. n = 3 independent experiments. Data are represented as mean ± SEM of triplicate measurements; *p < 0.05, **p < 0.01, and ***p < 0.001.

Next, we performed RNA-seq analysis on *SETD5*-depleted HCT116 cells to determine the biological function of SETD5 (Fig. 5A). Our findings revealed that SETD5 is involved in various biological processes, including colorectal cancer and PI3K-AKT signaling. We selected the TOP10 oncogenes that were most reduced under *SETD5* depletion conditions to select targets that SETD5 largely regulates (Fig. 5B). Seven of these genes were found to be involved in PI3K-AKT signaling, which was consistent with our previous finding in a study of esophageal squamous cell carcinoma cells^15^. qRT-PCR data confirmed that the mRNA expression levels of the target genes (*CD33, FXYD3, ALDH1A3, SMAD7, and ITGB8*) were significantly lower in *SETD5*-depleted HCT116 cells than in control cells (Fig. 5C). We investigated whether SETD5 also influences the transcription of isoforms other than *AKT3* because AKT is the central mediator of the PI3K-AKT pathway and has three isoforms (*AKT1, AKT2*, and *AKT3*)^37,38^. *AKT2* and *AKT3* expression was reduced in *SETD5*-depleted HCT116 cells, but not *AKT1* (Fig. 5D), and the reduced AKT protein levels were confirmed by western blotting under the same conditions (Fig. 5E), indicating the role of *AKT2* and *AKT3* in the SETD5-regulated pathway.

We speculated that SETD5 regulates gene transcription by controlling RNA Pol II recruitment to the promoter region because *O*-GlcNAcylation of RNA Pol II is required for its occupancy of promoter DNA and the formation of PIC complexes^23,26^. We used a ChIP assay to determine whether *SETD5* deficiency affected RNA Pol II occupancy. Our findings revealed that except for *CD33,* the enrichment of RNA Pol II within the promoter regions of six PI3K-AKT-related target genes and CD133, a SETD5-regulated CSC marker, was significantly decreased compared to that in control cells. In addition, RNA Pol II occupancy in promoters of WNT target genes (*KLF4, DKK1*, and *LGR5*) was also significantly reduced in the *SETD5*-depleted HCT116 cells (Supplementary Fig. S5). In contrast, *SETD5* depletion had no effect on RNA Pol II occupancy on the promoter of CD44, a non-SETD5-regulated CSC marker (Fig. 5F). These findings suggest that SETD5 is required for RNA Pol II recruitment to promoter regions, resulting in transcriptional activation of target genes. These findings support the notion that SETD5 is involved in activating the PI3K-AKT signaling pathway by recruiting RNA polymerase II to PI3K-AKT pathway-related gene promoters.

### Depletion of *SETD5* abrogates the acquisition of stem cell-like phenotypes and *O*-GlcNAcylation of RNA polymerase II in colorectal cancer cells

**Figure 6:**
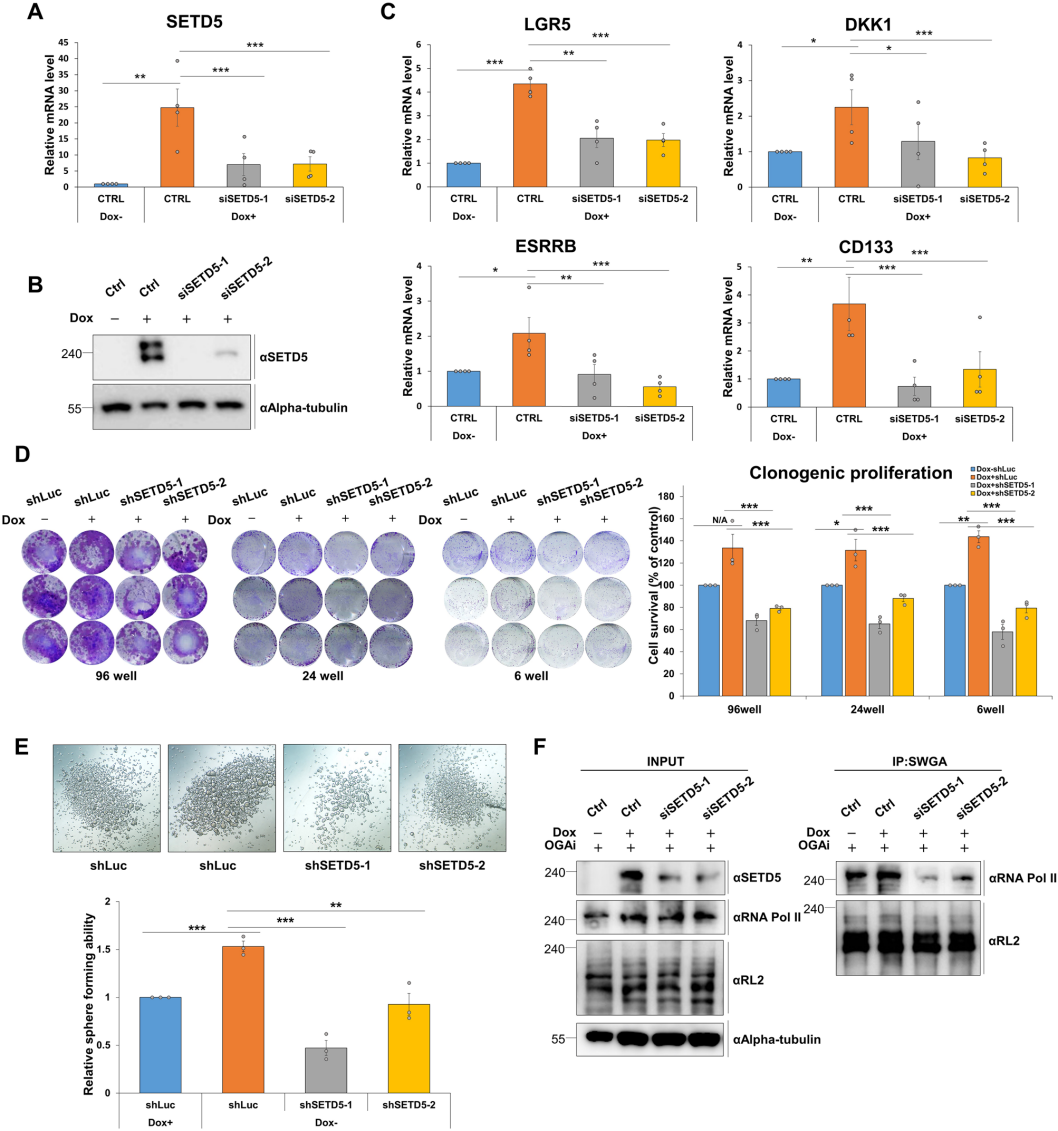
The acquisition of stem cell-like phenotypes and RNA Pol II *O*-GlcNAcylation in the SETD5-overexpressed CRC cells was nullified by the *SETD5* depletion. (**A**,**B**) The *SETD5* mRNA expression level was determined by qRT-PCR (**A**) and the protein level was examined by western blot (**B**) in Tet-inducible SETD5-based SW480 cells. Two siSETD5s targeting different mRNA sequences were used for transfection of host cells, and 100 ng/ml of doxycycline was treated to induce the SETD5 overexpression for 24 h. Symbols used: Dox(−), mock treatment without doxycycline; Dox(+), treated with doxycycline. (**C**) The mRNA levels of CSC marker genes (*ESRRB, CD133*) and WNT target genes (*LGR5, DKK1*) were examined by qRT-PCR when the *SETD5* was depleted by siSETD5s in the SETD5-overexpressed SW480 cells. The Dox-inducible overexpression of SETD5 and siRNA-based *SETD5* depletion were done as same in (**A**,**B**) of this figure. n = 3 independent experiments. (**D**) Clonogenic proliferation was investigated in Tet-inducible SETD5-based SW480 cells, which are stably expressing shRNAs targeting the *SETD5* mRNA. The SETD5 overexpression was induced by 100 ng/ml of Dox treatment. Images of stained cells were taken from plates with different well numbers (left panel). The percentage of the alive cells was quantified by measuring the colony-containing areas from the image of the left panel (right panel). The cell survival rate of the control KD cells (shLuc) was set as 100%. n = 3 independent experiments. (**E**) Tumor-initiating ability of Tet-inducible SETD5-based SW480 cells, which are stably expressing shRNAs targeting the *SETD5* mRNA (*shSETD5-1* & *shSETD5-2*). Overexpression of SETD5 was induced by 100 ng/ml of Dox treatment. Phase contrast images of tumorspheres derived from Tet-SETD5 SW480 cells (upper panel). Quantification of the sphere-forming cells in *SETD5* knockdown cells (bottom panel). The spheres-derived cell viability from the control KD cells (shLuc) was set as 1. n = 3 independent experiments. (**F**) The *O*-GlcNAcylation of RNA Pol II was determined by immunoprecipitation using sWGA beads in Tet-inducible SETD5-based SW480 cells. The Dox-inducible overexpression of SETD5 and siRNA-based *SETD5* depletion were done as same in (**A**,**B**) of this figure. Data are represented as mean ± SEM of triplicate measurements; *p < 0.05, **p < 0.01, and ***p < 0.001. Symbols used: Ctrl, control siRNAs; sWGA, IP using succinylated wheat germ agglutinin beads for the enrichment of *O*-GlcNAcylated proteins.

We used siRNA or shRNA to target the different regions within the mRNA sequences of *SETD5* to determine whether the acquisition of stem cell-like phenotypes and RNA Pol II glycosylation by Dox-inducible overexpression of SETD5 was nullified by *SETD5* depletion. qRT-PCR and western blotting were used to confirm the mRNA and protein expression levels of SETD5 in the Tetracycline-inducible system of SW480 cells (Fig. 6A and B). Furthermore, our results showed that *SETD5* depletion inhibited the overexpression of CSC markers, including *LGR5, DKK1, ESRRB*, and *CD133,* and PI3K-AKT-related genes, which was comparable to the expression levels without Dox treatment (Fig. 6C and Supplementary Fig. S6). Thus, these findings imply that SETD5 overexpression is critical for the induction of CSC marker gene expression. Moreover, the Dox-inducible SETD5 overexpression-induced increase in cell survival rates and tumor-initiating ability was abolished in *SETD5*-depleted SW480 cells but not in control knockdown cells (Fig. 6D and E). In addition, in SW480 cells overexpressing Flag-tagged SETD5, siRNA-based *SETD5* deficiency prevented SETD5-mediated *O*-GlcNAcylation of RNA Pol II (Fig. 6F). In summary, overexpression of SETD5 in colon cancer cells induces the expression of CSC marker genes, resulting in the gain of stem cell-like properties in colorectal cancer cells, such as increased survival rates and tumor-initiating ability, and the promotion of RNA Pol II *O*-GlcNAcylation. However, subsequent *SETD5* depletion prevented the acquisition of stem cell-like phenotypes and *O*-GlcNAcylation of RNA polymerase II, suggesting overexpressed SETD5 plays a direct role in the induction of stem cell-like phenotypes in CRC cells.

## Discussion

In this study, we found that aberrant overexpression of SETD5 in patients with colorectal cancer is associated with CD133, a cancer stem cell marker, and induces the acquisition of stem cell-like phenotypes in CRC cells. In addition, SETD5 promotes the expression of PI3K-AKT pathway-related genes and several CSC markers. Our findings revealed a functional relationship between SETD5, OGT, and RNA polymerase II. We concluded that SETD5 mediates OGT-dependent *O*-GlcNAcylation of RNA Pol II, which is involved in the gain of stemness in CRC cells because it interacts with both OGT and RNA Pol II, the OGT-catalyzed glycosylation of RNA Pol II is dependent on SETD5. Furthermore, the interaction between SETD5 and RNA Pol II weakens in *OGT*-depleted cells.

The effects of SETD5 on gene transcription were addressed in this study. During the early developmental stage, neuronal-related genes and stem cell maintenance-related genes are differentially regulated depending on the SETD5 dosage^4,9^. Owing to the fact that SETD5 can reprogram chromatin at target gene promoters via mutual interaction with the transcriptional repression-related HDAC3 complex and the transcriptional initiation-related PAF1 complex, diseases caused by SETD5 dysregulation may develop in a dosage-dependent and partner-dependent manner^4,5,9^. Similar to bivalent chromatin with active histone markers (H3K4me3) and inactive markers (H3K27me3), the mutual interaction of SETD5 with partners of opposite functions may be required for rapid response on the arrival of differentiation signals^39^. *SETD5* haploinsufficiency in mESCs caused an increase in PAF1-mediated RNA Pol II occupancy at the TSS of neuronal-related genes^4^, while genes related to stem cell identities, such as *KLF4* and *ESRRB,* were downregulated in *SETD5* null mESCs, suggesting a positive role for SETD5 in maintaining stem cell identity^9^. This suggests that fine-tuned SETD5 expression is critical for properly regulating target genes. Unlike stem cell marker proteins, such as Oct4 and Nanog, SETD5 is required for both stem cell identity and differentiation processes, such as neurodevelopment. Most differentiated somatic cell types should have turned off SETD5-mediated transcription of stem cell maintenance genes. However, aberrant overexpression of SETD5 may cause reactivation of stem cell marker genes via PAF1-mediated recruitment of RNA Pol II at their TSS, contributing to the stem cell-like phenotypes of cancer cells. We found that ectopic overexpression of SETD5 caused upregulation of CSC marker genes such as *CD133*, *KLF4, ESRRB,* and PI3K-AKT pathway-related genes, resulting in stemness gain in CRC cells. This stemness was nullified by the *SETD5* depletion, and importantly, the RNA Pol II occupancy at promoters of PI3K-AKT pathway-related genes and CD133, a CSC marker, was decreased in the *SETD5*-depleted cells. Aberrant SETD5 overexpression is associated with cancer progression in various cancer types, which is consistent with our findings^13–16^. Several studies have revealed that the cross-talk between Wnt/β-catenin and PI3K/AKT pathways is associated with cancer progression and the maintenance of CSC function in colorectal cancer^37,40,41^. In addition, the PI3K/AKT pathway-related genes including FXYD3, SMAD7, and ITGB8, are involved in stem cell-like properties in various cancer types, which are transcriptionally regulated by SETD5^41–47^. Therefore, the findings support the hypothesis that aberrant SETD5 upregulation reactivates the transcription of stem cell marker genes via SETD5-mediated chromatin reprogramming, resulting in cancer cells gaining stemness. In this present study, we found that SETD5 overexpression promotes the expression of PI3K-AKT pathway-related genes and CSC marker genes by increasing RNA Pol II occupancy at the promoters. Nonetheless, the overall target selection by overexpressed SETD5 remains to be addressed by genome-wide mapping using ChIP-sequencing.

Our findings support that SETD5 mediates RNA Pol II *O*-GlcNAcylation via OGT recruitment. We proposed a model in which SETD5 is one of the adaptor proteins responsible for providing OGT substrate specificity. A long-standing question in the field of *O*-GlcNAcylation is how the paired enzymes of OGT and OGA recognize their substrates. There are three plausible hypotheses: consensus sequence model, non-specific recognition, and adaptor protein hypothesis^17^. Recent studies have focused on the effect of *O*-GlcNAcylation on transcription factors and epigenetic regulators^17,19–22,25,48–50^. The work on glycosylation-dependent regulation of epigenetic programs allows the adaptor protein hypothesis to be expanded. In particular, host cell factor C1 (HCF1) forms complexes with approximately 50% of nuclear OGT, linking to various histone modifications^17^. BAP1 (a component of polycomb repressive deubiquitinase), PPAR-γ co-activator 1α (PGC1α), and TET1 are *O*-GlcNAcylated by OGT via the assistance of HCF1, suggesting that they are major adaptors for OGT-mediated glycosylation^17^. TET functions as an adaptor protein for OGT-catalyzed *O*-GlcNAcylation of histone proteins^51^. According to recent studies, OGT-catalyzed *O*-GlcNAcylation of RNA Pol II is critical for PIC formation and transcription cycling, including PIC assembly, initiation, and elongation^17,23–26,36^. The findings point to the formation of complexes between OGT and RNA Pol II, OGT and OGA activity recruitment for transcription during PIC assembly, OGT-dependent occupancy of RNA Pol II at the B-cell specific promoters, exclusive enrichment of *O*-GlcNAcylated form of RNA Pol II at TSS, and enrichment of most of OGT and *O*-GlcNAcylation at TSS^23,25,26,51,52^. The recognition of RNA Pol II by OGT remains unknown despite the extensive study. We found that SETD5 interacts with OGT and RNA Pol II. *O*-GlcNAcylation of RNA Pol II was, more importantly, SETD5-dose dependent. SETD5 is also *O*-GlcNAcylated, with glycosylation primarily occurring in the C-terminus. SETD5 deletion mutants lacking the C-terminal region exhibited reduced interaction with both RNA Pol II and OGT, suggesting a role for SETD5 *O*-GlcNAcylation in RNA Pol II interaction. Moreover, the interaction of SETD5 with RNA Pol II weakens in *OGT*-depleted cells, indicating the importance of *O*-GlcNAcylation in this interaction. These findings support the hypothesis that SETD5 mediates *O*-GlcNAcylation of RNA Pol II via SETD5-dependent OGT recruitment. Thus, we propose that SETD5 is an adaptor protein that determines the OGT substrate specificity. In this present study, we were unable to identify the amino acid residues responsible for the *O*-GlcNAcylation of SETD5 due to the abundance of potential sites. Identifying specific *O*-GlcNAcylation sites for SETD5 may be necessary for future work to understand the impact of SETD5 *O*-GlcNAcylation on OGT-catalyzed glycosylation of RNA Pol II. Additionally, we investigated the potential role of SETD5-OGT-mediated *O*-Glycosylation of RNA Pol II in driving CSC marker gene transcription. However, future studies might investigate whether the SETD5-OGT-mediated glycosylation of RNA Pol II is sufficient for the induction of CSC marker gene expression or whether other factors, including transcriptional activators and epigenetic regulators, are involved.

In summary, our findings revealed that SEDT5-mediated induction of CSC marker genes and PI3K-AKT pathway-related genes result in the gain of stem cell-like phenotypes in CRC cells. OGT-catalyzed *O*-GlcNAcylation of RNA Pol II is a SETD5-dosage-dependent event that promotes CSC marker gene expression in CRC cells. Our findings support the idea that SETD5 is identified as one of the adaptor proteins responsible for OGT substrate specificity and that SETD5-OGT-mediated *O*-GlcNAcylation of RNA Pol II is involved in the transcriptional induction of CSC markers and PI3K-AKT pathway-related genes, thereby contributing to the acquisition of stemness in CRC cells.

## Materials and methods

### Cell culture, construction of stable cell lines, and chemicals

CRC cell lines (HCT116, HT29, & SW480) were cultured in RPMI-1640 medium (Welgene, Gyeongsangbuk-do, South Korea) containing 10% fetal bovine serum (FBS) (Atlas Biologicals, CO, USA) and 100 U/ml penicillin-100 μg/ml streptomycin (Corning). OGA inhibitor (Thiamet G, Cat. #SML0244/ Sigma-Aldrich, MO, USA) or OGT inhibitor (OSMI-1, Cat. #SML-1621/ Sigma-Aldrich) was used as follows: cells transfected with plasmid DNA were treated with 100 nM OGA inhibitor (OGAi) or 50 µM OGT inhibitor for 24 h. We cloned a pair of *SETD5* shRNA targeting the coding sequence of *SETD5* into a lentivirus-based pLKO.1 TRC cloning vector (Addgene; kindly provided by Dr. David Root) and produced lentiviral particles in 293FT cells to create stable knockdown cell lines. HCT116 cells were infected with these lentiviral particles and selected by culturing in a medium supplemented with puromycin (2 μg/ml; Santa Cruz Biotechnology, TX, USA). The primers used were shSETD5 (CDS), 5′-GCCATCAGACTTACGGACTAT-3′; and SETD5 (UTR), 5′-GTCTGAAGCAGAGACTATAAA-3′. Chemically synthesized siRNAs were also used to knockdown SETD5 and OGT. The primers used were siOGT, 5′-UAAUCAUUUCAAUAACUGCUUCUGC-3′ (forward) and 5′-GCAGAAGCAGUUAUUGAAAUGAUUA-3′ (reverse); siSETD5-1, 5′-CUGUAUCCAGUGAUCAUGA-3′ (forward) and 5′-UCAUGAUCACUGGAUACAG-3′ (reverse); and siSETD5-2, 5′-GACUAGAACUCAGCACCU-3′ (forward) and 5′-UAGGUGCUGAGUUCUAGU-3′ (reverse). We used the Lenti-X Tet-On 3G inducible expression system (Clontech/Takara Bio, Shiga, Japan) to create a conditional SETD5-overexpression cell line, following the manufacturer's protocol. Briefly, the coding sequence (CDS) of *SETD5* was cloned into the pLVX-TRE3G-ZsGreen1 vector. SW480 cells were infected with viral particles after being produced in 293FT cells and selected by culturing in a medium supplemented with puromycin (2 μg/ml; Santa Cruz Biotechnology). The CRC cell lines (HT116, HT29, SW480) and 293FT were obtained from the American Type Culture Collection (Manassas, VA, USA).

### Cloning and site-directed/deletion mutagenesis

We used the mammalian expression vector pCMV-3Tag-6 (Addgene, Teddington, UK) to tag proteins with the 3xFlag epitope at the N-terminus and pCMV-3Tag-2 (Addgene, Teddington, UK) to tag proteins with the 3xMyc epitope at the N-terminus. cDNA was used as a template to amplify the coding sequence of the target genes after being reverse transcribed from RNA extracted from HCT116 cells using oligo-dT primers (Thermo Fisher Scientific, Waltham, MA, USA). The amplified target gene sequences were cloned into plasmid vectors using appropriate restriction enzymes (Enzynomics, Daejeon, Korea) and a DNA ligation kit (Takara Bio, Shiga, Japan), according to the manufacturer’s protocols. In addition, plasmids containing wild-type target genes were used as templates to amplify mutant target genes for site-directed/deletion mutagenesis. Supplementary Table S1 lists the primers used for cloning.

### Western blotting

The cells were harvested by centrifugation. The pellets were resuspended in immunoprecipitation assay buffer containing 150 mM NaCl, 50 mM Tris (pH 8.0), 1% NP-40, 0.5% sodium deoxycholate, 0.1% SDS, and protease inhibitors, and incubated on ice. After centrifugation, supernatants containing the proteins of interest were collected. The Bradford assay was used to determine protein concentration. For Western blot analysis, proteins were separated using 10% SDS-PAGE and transferred to polyvinylidene fluoride membranes (EMD Millipore, Billerica, MA, USA). Following that, the membranes were treated with primary antibodies. The following primary antibodies were used: SETD5 (sc-515645; Santa Cruz Biotechnology), CD133 (130-092-395; Miltenyi Biotec, Bergisch Gladbach, Germany), RNA polymerase II (ab26721; Abcam, Cambridge, UK), RL2 (MA1-072; Invitrogen), OGT (O6264; Sigma-Aldrich, MO, USA), CTR9 (ab84487; Abcam), AKT (4691S; Cell Signaling, MA, USA), and TBl1XR1 (sc137083; Santa Cruz Biotech.), H3 (ab1791; Abcam), H3K9me1 (ab9045; Abcam), H3K9me2 (ab1220; Abcam), H3K9me3 (ab8898; Abcam), H3K27me3 (ab6002; Abcam), H3K4me3 (ab8580; Abcam), and H3K36me3 (ab9050; Abcam).

### RNA isolation and quantitative RT-PCR (qRT-PCR)

Total RNA was extracted using TRI reagent (TR118-200; MRC, Cambridge, UK) and chloroform (Merck, Kenilworth, NJ, USA) and reverse-transcribed into cDNA using a RevertAid First Strand cDNA Synthesis Kit (K1622; Thermo Fisher Scientific, Waltham, MA, USA) with oligo-dT primers. qRT-PCR was performed using SYBR Premix EXTM Taq II (RR82LRB; Takara Bio, Shiga, Japan) on a 7300 Real-time PCR system (Applied Biosystems, Foster City, CA, USA). Dissociation curves were generated to determine the presence of off-target amplification products or contaminants. Supplementary Table S2 lists the primers used. qRT-PCR data were quantified using at least three independent experiments, and results are presented as the mean ± SD.

### Clonogenic assay

To investigate clonogenic proliferation, 100 CRC cells were seeded in 96-well, 24-well, and 6-well plates (SPL, Gyeonggi-do, South Korea). After 10 d, the colonies were stained with crystal violet solution and scanned to determine the colony area. For quantification, the ImageJ software was used.

### TOP/FOP assay

A TOP/FOP assay was used to confirm the activity of Wnt/β-catenin signaling. In 24-well plates, we seeded 1.5 × 10^5^ *SETD5*-knockdown cells (SPL). After 24 h, TOP or FOP flash plasmids were transfected into cells with the SV40-Renilla plasmid (control for transfection) using Lipofectamine™ 2000-transfection reagent (Invitrogen). After 2 d, luciferase activity was detected using a dual-luciferase reporter assay system (Promega, Madison, WI, USA) and Promega GLOMAX 20/20 system according to the protocol of the manufacturer.

### RNA sequencing (RNA-seq)

RNA sequencing was conducted by Macrogen, Inc. (Seoul, Korea). RNA extracts from *SETD5*-knockdown HCT116 cells were subjected to cDNA library construction using the TruSeq Stranded mRNA LT Sample Prep Kit (Illumina, San Diego, CA, USA). The samples were tested for quality using the FastQC v0.11.5 software before being sequenced using a HiSeq 4000 sequencer (Illumina). The Kyoto Encyclopedia of Genes and Genomes (KEGG) database was used to determine the pathways of differentially expressed genes. The pathways were ranked using Fisher’s exact test with a *p*-value set as the significance threshold. The data discussed in this publication were deposited in NCBI’s SRA data and are accessible under SRA series accession number PRJNA940572.

### Protein isolation and digestion for Mass spectrometry (MS) analysis

Flag-tagged SETD5 transfected in 293 T cells by OGT co-transfection and ThiaMet-G treatment for hyper-*O*-GlcNAcylation condition. SETD5 was enriched using anti-FLAG immunoprecipitation, then separated using SDS-PAGE, and stained with Coomassie Brilliant Blue reagent. SDS-PAGE gel fraction for SETD5 was cut and in-gel tryptic digestion was performed. Destaining the target protein fraction was performed using 50% (v/v) ACN in 25 mM NH_4_HCO_3_. The fraction was then reduced by 20 mM DTT at 60 °C for 1 h and alkylated with 55 mM iodoacetamide at 25 °C for 45 min without light. Finally, the fraction was digested with trypsin at 37 °C overnight. The peptides were extracted using 50% (v/v) ACN in 5% (v/v) FA and 80% (v/v) in 5% (v/v) FA, respectively. This experiment was duplicated.

### MS analysis

The extracted peptide samples by in-gel digestion were suspended in 20 µl of solvent A (0.1% formic acid prepared in water; Optima LC/MS grade, ThermoFisher Scientific). The peptides were separated in PepMapTM RSLC C18 column (Thermo Fisher Scientific, San Jose, CA) with a linear gradient of 2 to 38% Solvent B (0.1% FA in ACN) over 65 min and at a flow rate of 300 nl/min. The sample was analyzed by Orbitrap Fusion Lumos Tribrid mass spectrometer (Thermo Fisher Scientific, San Jose, CA), interfaced with Easy nanoLC 1000 system (Thermo Fisher Scientific, San Jose, CA). The spray voltage was set to 1.8 kV. The temperature of the heated capillary was set at 275 °C. A data-dependent mode with one full MS scan followed by twenty MS/MS scans and a dynamic exclusion time of 20 s was used in the operation of the Orbitrap Fusion Lumos Tribrid mass spectrometer. In MS/MS scans, peptides were fragmented using higher energy collision dissociation (HCD). If oxonium product ions (m/z 204.0867, 138.0545) were observed in the HCD spectra, the peptides were fragmented with electron transfer/higher energy collision dissociation (EThcD) in a subsequent scan on the same precursor ion selected for HCD. The full scans were acquired at 300 to 1400 m/z. The first mass of HCD and EThcD MS/MS scans were 120 and 110 m/z. The resolutions of full MS scans and MS/MS scans (HCD and EThcD) were 120,000 and 300,000. In full scans, the advanced gain control target was 4 × 10^5^ and the maximum injection time was 100 ms. In MS/MS scans, the advanced gain control target was 5 × 10^4^, the maximum injection time was 54 ms, and the isolation window was set to 1.6 m/z. In EthcD MS/MS scans, the advanced gain control target was 8.5 × 10^4^, the maximum injection time was 54 ms, and the isolation window was set to 1.6 m/z. HCD collision energy was set to 28%. ETD reaction time was 50 ms and supplemental activation (SA) collision energy was 20%.

For MS data analysis, two raw files derived from duplicated experiments were used. The raw data were compared with a Uniprot human database (released in Nov. 2018, entry no. 174,355) using the SEQUEST HT search engine in Proteome Discoverer 2.2 (Thermo Fisher Scientific). Trypsin was selected as the proteolytic enzyme with a maximum allowance of up to two missed cleavages. Precursor mass error tolerance was set to 10 ppm and fragment mass error tolerance was set to 0.5 Da. The carbamidomethyl of cysteine was considered a fixed modification, and variable modification was set for the oxidation of methionine and HexNAc of serine and threonine with a maximum of three PTMs at one peptide. The weight of b and y ions was set to 0.5, and the weight of c and z ions was set to 1, respectively. Target FDR (Strict) and target FDR (Relaxed) were set to 0.01 and 0.05 respectively in the percolator node at the peptide spectrum match level and in the protein FDR validator node at the protein level. Protein grouping was carried out by using a strict parsimony principle to generate the final protein groups. The MS proteomics data have been deposited to the PRIDE repository with the dataset identifier PXD041141.

### Tissue specimens and ethics approval

141 formalin-fixed and paraffin-embedded samples were used in this study, including 98 adenocarcinomas, nine adenomas, and 34 hyperplastic polyp tissues from patients who underwent curative surgical resection for primary colon adenocarcinoma at Yanbian University Affiliated Hospital, Yanji, China, in 2000. The Institutional Review Board of Yanbian University Affiliated Hospital approved this retrospective study and conducted it in accordance with the Declaration of Helsinki of 1996 (IRB Code number: 82160594). According to the institutional guidelines, all patients provided written informed consent. Further, none of the patients had received preoperative chemotherapy or had undergone radiotherapy. Age, sex, differentiation, lymphatic invasion, vascular invasion, nerve invasion, T stage, lymphovascular invasion, TNM stage, and recurrence were all examined in the clinical and pathological reports. The median follow-up period was 112 months (range: 2–120 months). pTNM classification was followed according to the guidelines of the 2010 American Joint Committee on Cancer Staging Manual (AJCC 7th edition).

### Immunohistochemical staining

To quench endogenous peroxidase activity, microslide sections were deparaffinized with xylene, hydrated using a diluted alcohol series, and immersed in 3% H_2_O_2_ in methanol. At 98 °C for 30 min, the sections were treated with TE buffer (10 mM Tris and 1 mM EDTA, pH 9.3). The sections were then incubated for 60 min at room temperature with anti-SETD5 (1:300, Novus Biologicals, CO, USA), anti-CD133 (1:100, Novus Biologicals, CO, USA), or anti-CD44 (1:100, Abcam, Cambridge, UK) antibodies diluted in phosphate-buffered saline supplemented with Tween 20 (PBST) containing 3 mg/ml goat globulin (Sigma-Aldrich, MO, USA). Following that, three successive washes with buffer were performed. The sections were then incubated for 30 min at room temperature with an anti-mouse/rabbit antibody (Envision Plus, DAKO, Glostrup, Denmark). The chromogen used was 3,3′-diaminobenzidine (DAKO, Glostrup, Denmark). Finally, the sections were counterstained with Meyer’s hematoxylin. By omitting the primary antibodies, negative controls for immunostaining were generated.

### Evaluation of the immunohistochemical analysis

According to the staining intensity and proportion of positive stromal cells, immunohistochemical scores for SETD5, CD133, and CD44 were measured by pathologists without prior knowledge of the clinicopathological results. Scoring was as follows: 1, weak staining in < 50% or moderate staining in < 20% of stromal cells; 2, weak staining in $\ge$ 50%, moderate staining in 20–50%, or intense staining in < 20%; and 3, moderate staining in $\ge$ 50% or intense staining in $\ge$ 20%. Cases with scores of 2 or 3 for each protein expression were considered positive.

### Statistical analysis for immunohistochemical analysis

Correlations were examined using Pearson’s chi-squared or Fisher’s exact test. Overall survival (OS) and disease-free survival (DFS) were determined using the Kaplan–Meier method and compared using the log-rank test. Survival was calculated from the date of surgery. For the multivariate analysis, the Cox proportional hazards model was used. Clinicopathological factors that were statistically significant in univariate analysis were included as covariates in the multivariate analysis. Hazard ratios (HR) and 95% confidence intervals (CI) were calculated for each factor. All tests were two-sided, with a P < 0.05 considered significant. Statistical analysis was performed using the SPSS statistical software (SPSS Inc., Chicago, IL, USA).

### Chromatin immunoprecipitation (ChIP) Assay

A ChIP assay was performed using a SimpleChIP Enzymatic Chromatin IP Kit (Cell Signaling #9002S) according to the instruction of the manufacturer. Briefly, the cells were cross-linked in situ by adding 37% formaldehyde to a final concentration of 1%, incubated at room temperature for 10 min, and then incubated with glycine for 5 min. Chromatin was digested and immunoprecipitated using IgG (Cell Signaling Technology #2729) and RNA polymerase II (ab4729; Abcam) antibodies overnight at 4 °C. PCR amplification was performed on purified DNA using primers specific to the promoter fragments of the indicated genes. Supplementary Table S3 lists the primers used.

### Immunoprecipitation (IP) assay

Harvested cells were lysed with an IP lysis buffer (1% Triton X, 10% glycerol in 20 mM Tris–HCl [pH8.0] containing 137 mM NaCl, 2 mM ETDA) or 1% NP40 buffer (1% NP-40, 10% glycerol, 50 mM Tris–HCl, pH 7.4, 5 M NaCl) supplemented with protease inhibitors. Following that, they were incubated with anti-FLAG affinity gel (Sigma-Aldrich) or indicated antibodies overnight at 4 °C under rotation. The next day, the anti-FLAG affinity gel binding sample goes directly into the washing stage. Antibody binding samples were added to protein A or G beads and incubated for 3–5 h at 4 °C under rotation. After washing the beads, the proteins were eluted with SDS sample buffer and boiled. The eluted protein samples were subjected to western blot analysis.

### sWGA affinity purification for glycosylation assay

The procedures for in vivo glycosylation assay were described elsewhere^20^. Briefly, cells were lysed with 1% NP40 buffer (1% NP-40, 10% glycerol, 50 mM Tris–HCl, pH 7.4, 5 M NaCl) supplemented with protease inhibitors. Protein concentrations of cell lysates were determined by the Bio-Rad protein assay (Hercules, CA, USA). They were incubated with agarose-conjugated succinylated wheat germ agglutinin (sWGA, Vector Laboratories, Burlingame, CA, USA) overnight at 4 °C under rotation. Immunoprecipitates were washed five times with cold PBS, eluted with SDS sample buffer, and subjected to SDS-PAGE and western blotting.

### Tumorsphere assay

To investigate the ability of HCT116 cells to form tumorspheres, we seeded 2 × 10^4^ cells in ultra-low-attachment 6-well plates (Corning, NY, USA) in Dulbecco's Modified Eagle Medium: Nutrient Mixture F-12 (DMEM: F12) (Welgene) containing 20 ng/ml epidermal growth factor (EGF), 20 ng/ml fibroblast growth factor (FGF), 5 µg/ml insulin, and 1X B27 supplement. The medium was added to the cells every 2 days. After 7 days, viable cell-forming tumorspheres were detected using the CCK-8 assay (Dojindo, Kumamoto, Japan).

### Statistical analysis

Data are expressed as mean ± SEM or mean ± SD. Data between the control and experimental groups were analyzed using two-tailed Student’s t-tests. The significance levels were as follows: *p < 0.05, **p < 0.01, and ***p < 0.001.

### Supplementary Information

Supplementary Information 1.

Supplementary Information 2.

Supplementary Information 3.

Supplementary Information 4.

## Data Availability

All data generated or analyzed during this study are included in this published article and its supplementary information files. The RNA-seq raw datasets generated during and/or analyzed during the current study are available in the NCBI Sequence Read Archive (SRA) repository, [SRA series accession number PRJNA940572; https://www.ncbi.nlm.nih.gov/bioproject/PRJNA940572]. The MS proteomics data have been deposited to the PRIDE repository with the dataset identifier PXD041141 [https://www.ebi.ac.uk/pride/archive/projects/PXD041141/private; Username: reviewer_pxd041141@ebi.ac.uk; Password: PkaYxVQa].

## Abbreviations

ASD: Autism spectrum disorder
CRC: Colorectal cancer
CSC: Cancer stem cell
CTD: C-terminal domain
ID: Intellectual disability
IP: Immunoprecipitation
mESCs: Murine embryonic stem cells
OGA: *O*-GlcNAcase
OGAi: OGA inhibitor
OGT: *O*-GlcNAc transferase
PIC: Preinitiation complex
Pol II: RNA polymerase II
qRT-PCR: Quantitative RT-PCR
SETD5: SET-domain-containing protein 5
shRNA: Small interfering RNA derived from vector
TSS: Transcriptional start site
