# Supplementary material for: SETD5 regulates the OGT-catalyzed O-GlcNAcylation of RNA polymerase II, which is involved in the stemness of colorectal cancer cells
Source: Sci Rep. 2023 Nov 14;13:19885. doi: 10.1038/s41598-023-46923-1 (PMC10646014; doi:10.1038/s41598-023-46923-1)

| Identified O-GlcNAc sites in c-term of SETD5 |
| --- |
| S893 |
| S1118 |
| S1135 |
| S1138 |
| S1152 |
| S1171 |
| S1222 |
| T1249 |
| S1251, S1253 |
| S1258 |
| S1365 |
| S1380 |
| S1381, S1384 |
| S1388 |
| S1390 |
| S1406, S1409 |
| S1409 |
| S1433 |
| S1437 |

S893

EThcD #16038

$[M+2H+HexNAc]^{+2}$= 1076.5192

**NGYSLM(ox)FSPVTSLTTASR**

red = O-GlcNAc

ox = Oxidation

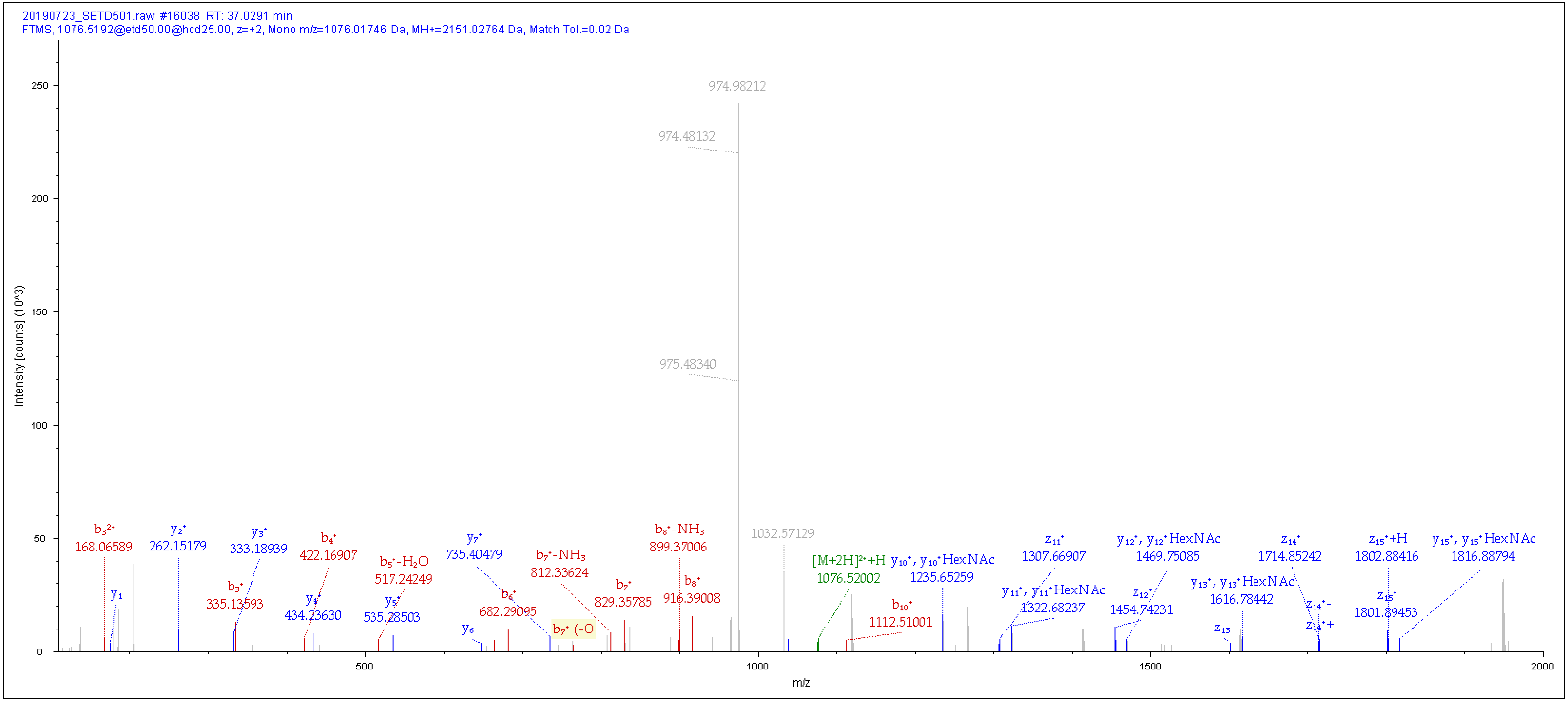

S1118

EThcD #2710

$[M+3H+HexNAc]^{+3}$= 846.0505

**SAGAGQGSSNSVSDTGAHGVQGSSAR**

red = O-GlcNAc

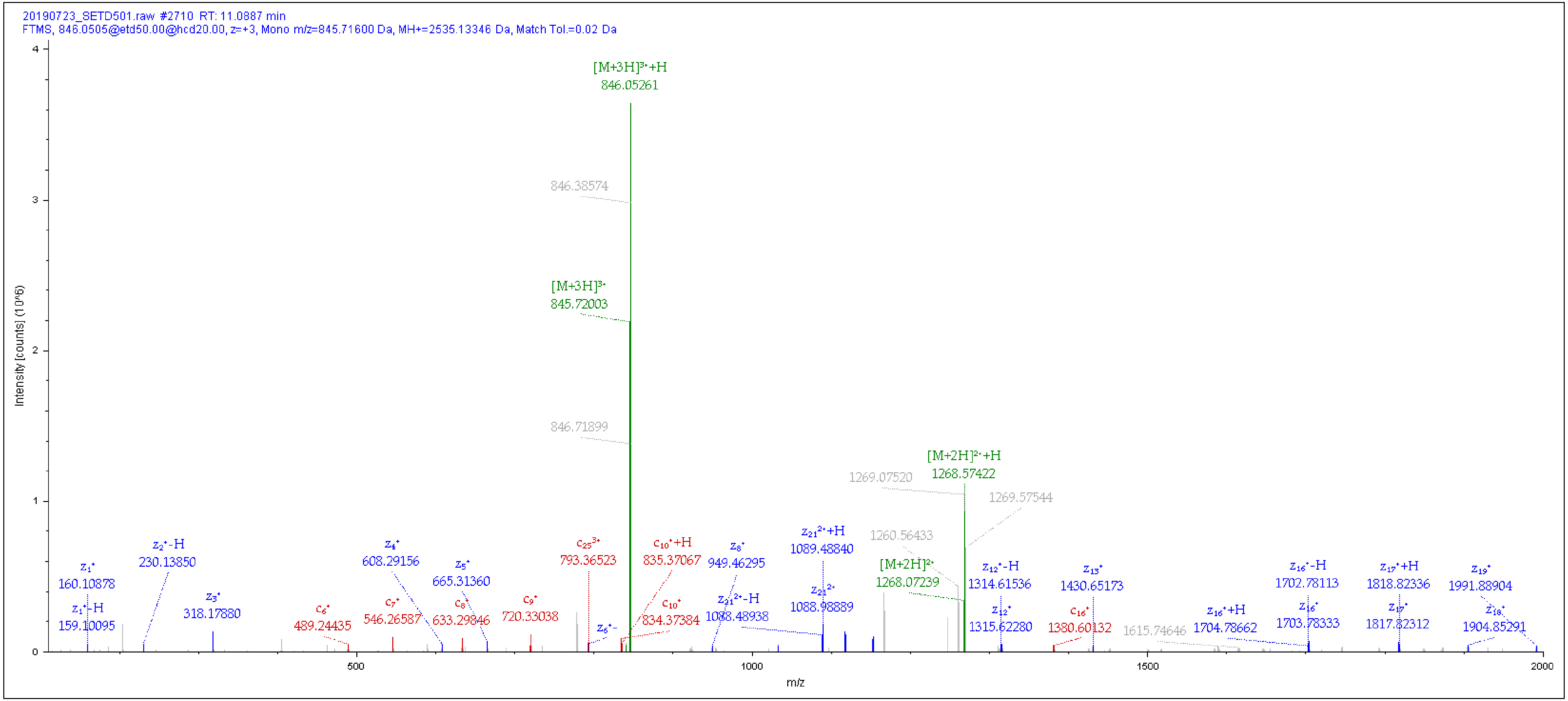

S1135

EThcD #2883

[M+4H+HexNAc]$^{+4}$= 624.0403

**KFSPSHSSM(ox)SHLEAVSPSDSR**

red = O-GlcNAc

ox = Oxidation

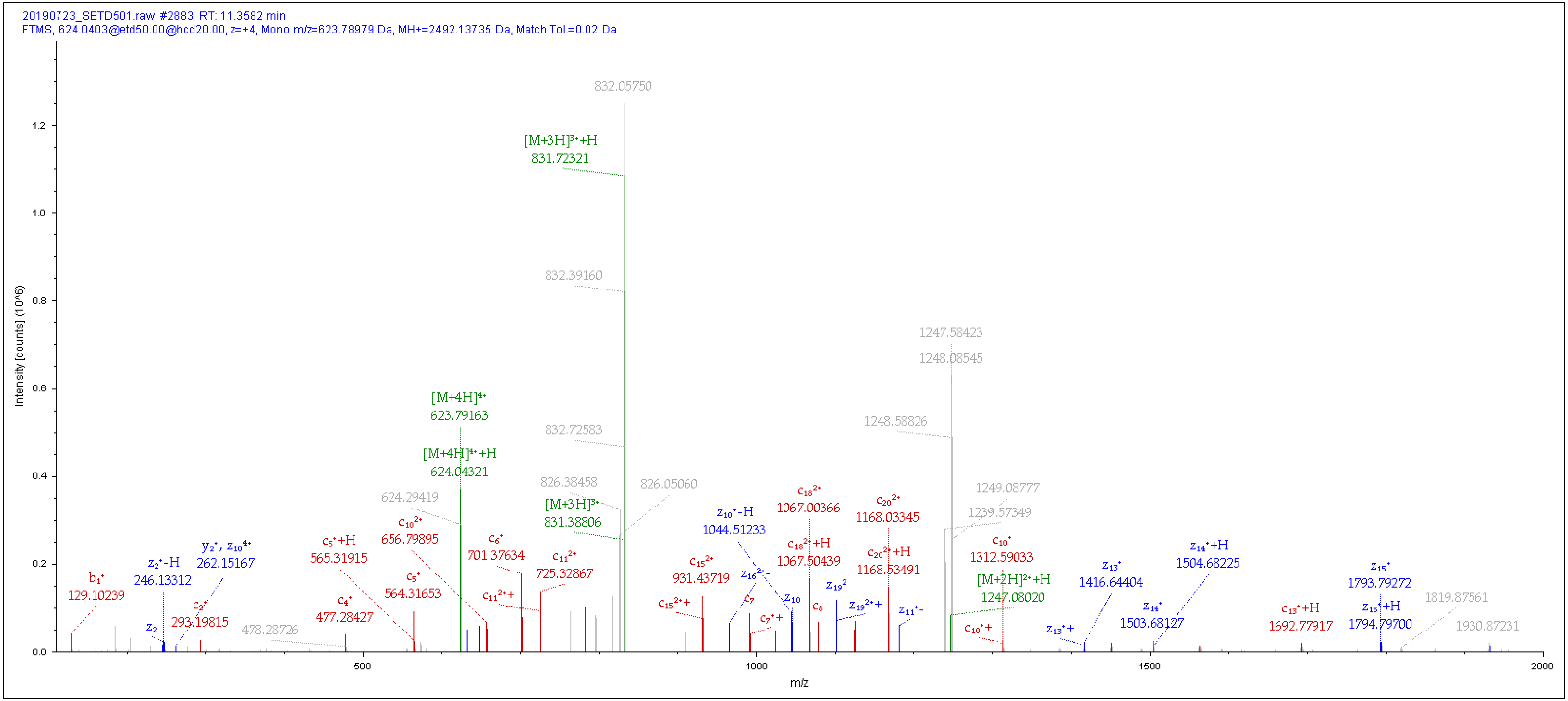

S1138

EThcD #3864

[M+3H+HexNAc]$^{+3}$= 789.0202

**KFSPSHSSM(ox) SHLEAVSPSDSR**

red = O-GlcNAc

ox = Oxidation

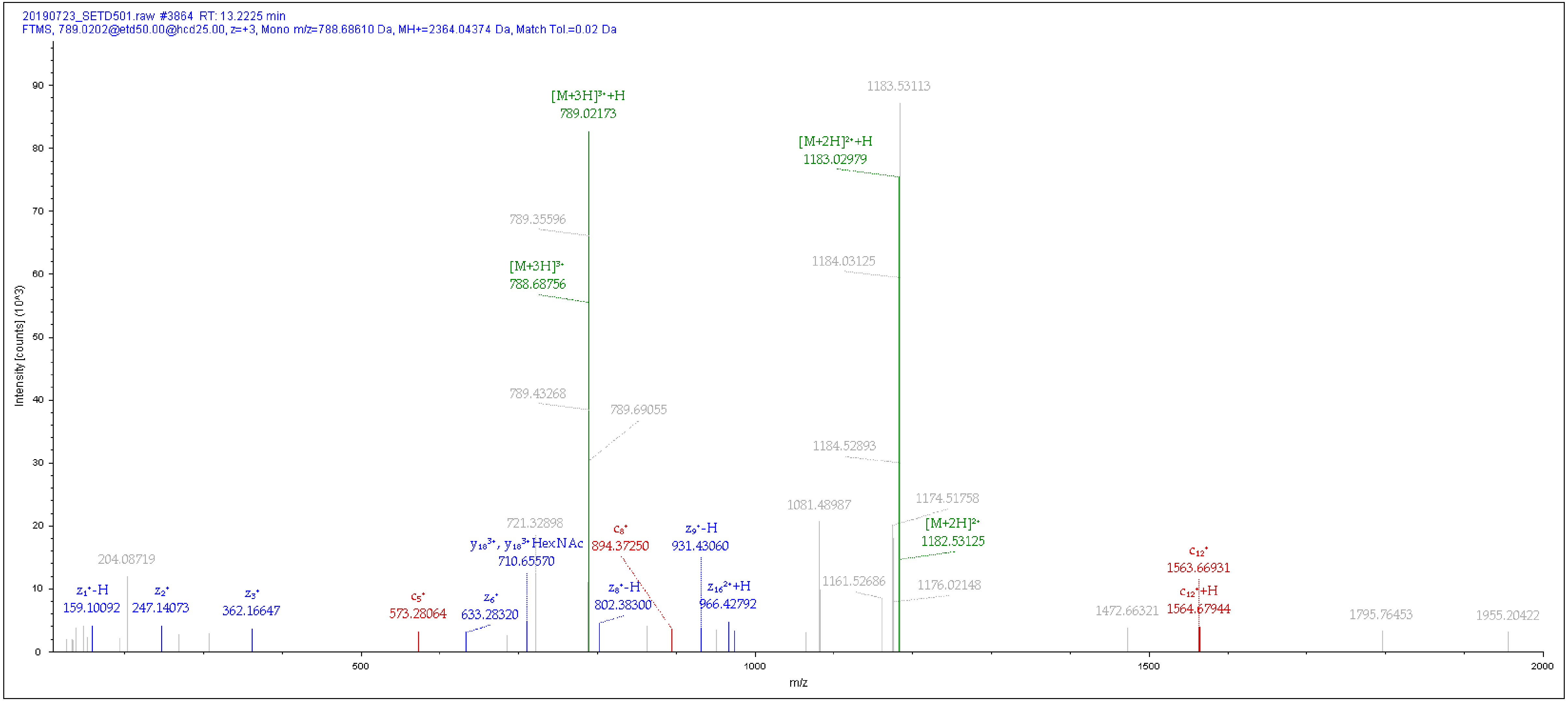

S1152

EThcD #1860

[M+4H+HexNAc]$^{+4}$= 502.4807

**GTSSSHC(carba)RPQENISSR**

red = O-GlcNAc

carba = Carbamidomethyl

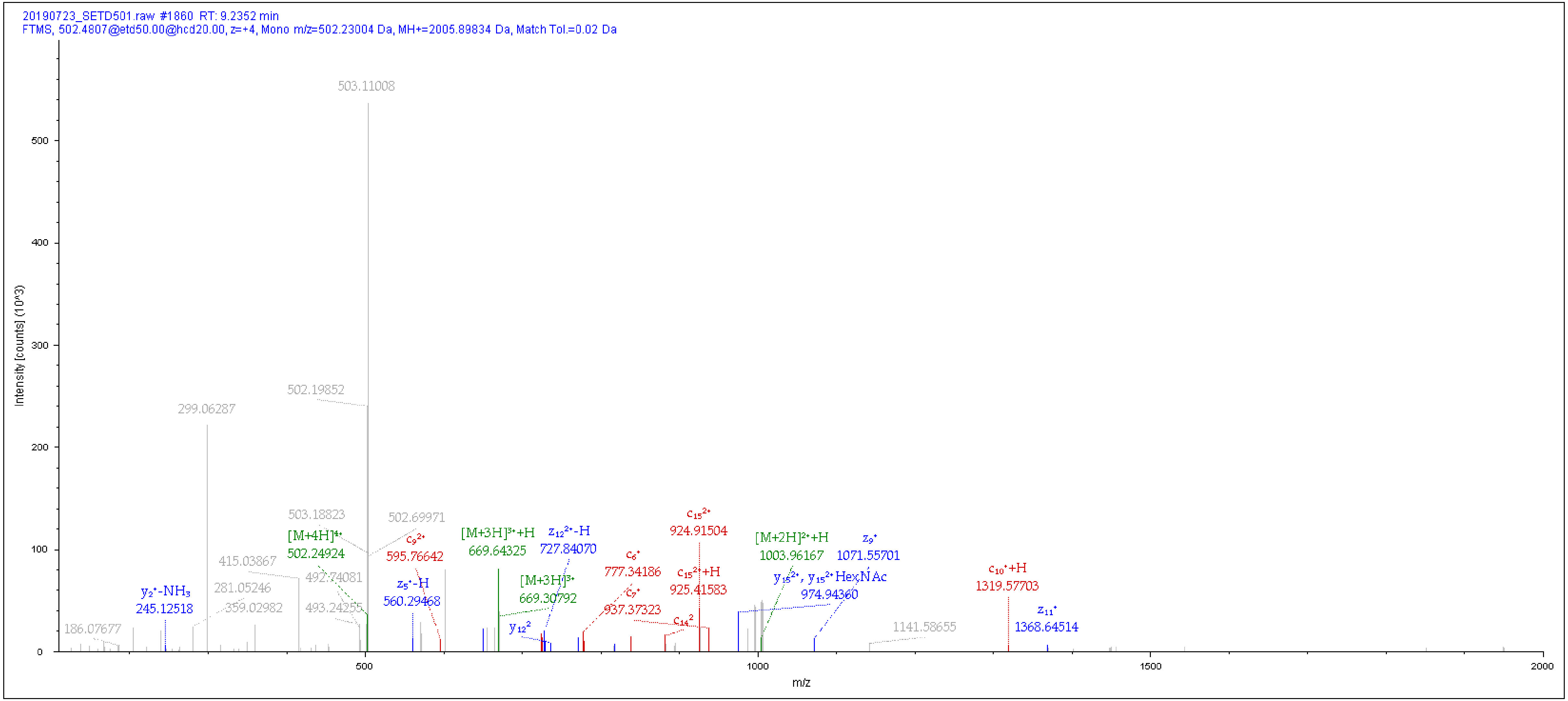

S1171

EThcD #9519

[M+2H+HexNAc]+2= 662.3163

**WM(oxi)VPTSVER**

red = O-GlcNAc

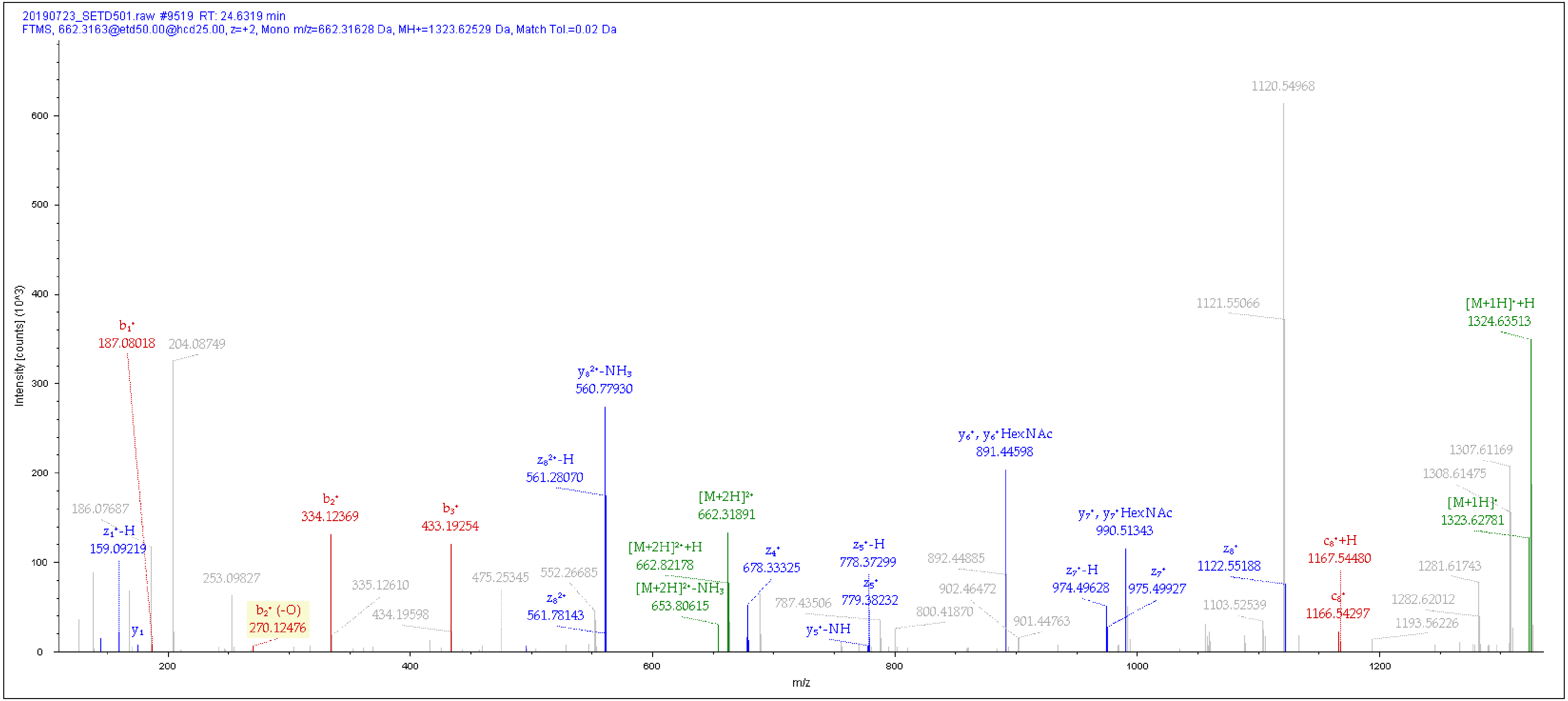

S1222

EThcD #11344

[M+2H+HexNAc]$^{+2}$= 1040.4678

**DSDPADGEGPETLSSALSK**

red = O-GlcNAc

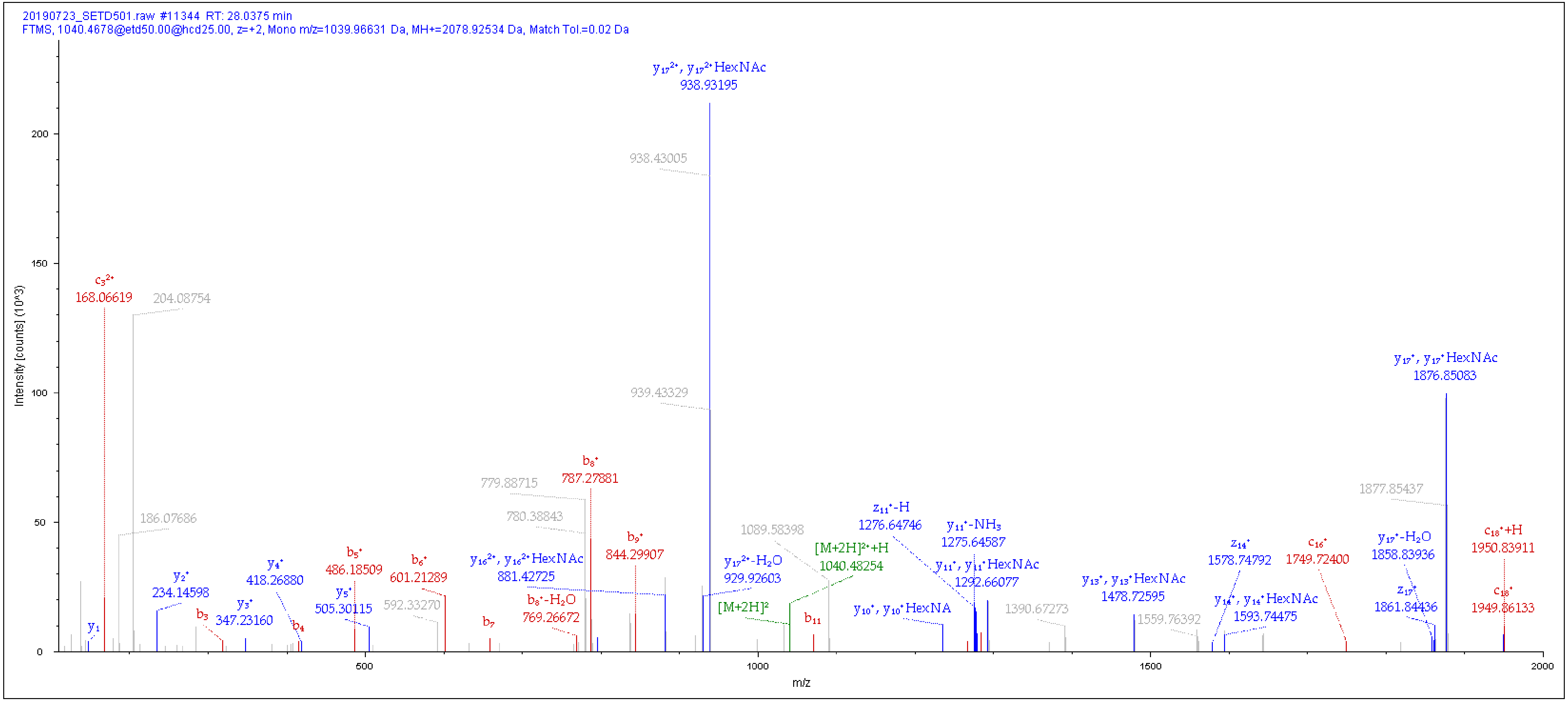

T1249

EThcD #15724

[M+3H+HexNAc]$^{+3}$= 1137.2053

**YSYQLLQC(carba)DSPRTESQSLLQQSSSPFR**

red = O-GlcNAc

carba = Carbamidomethyl

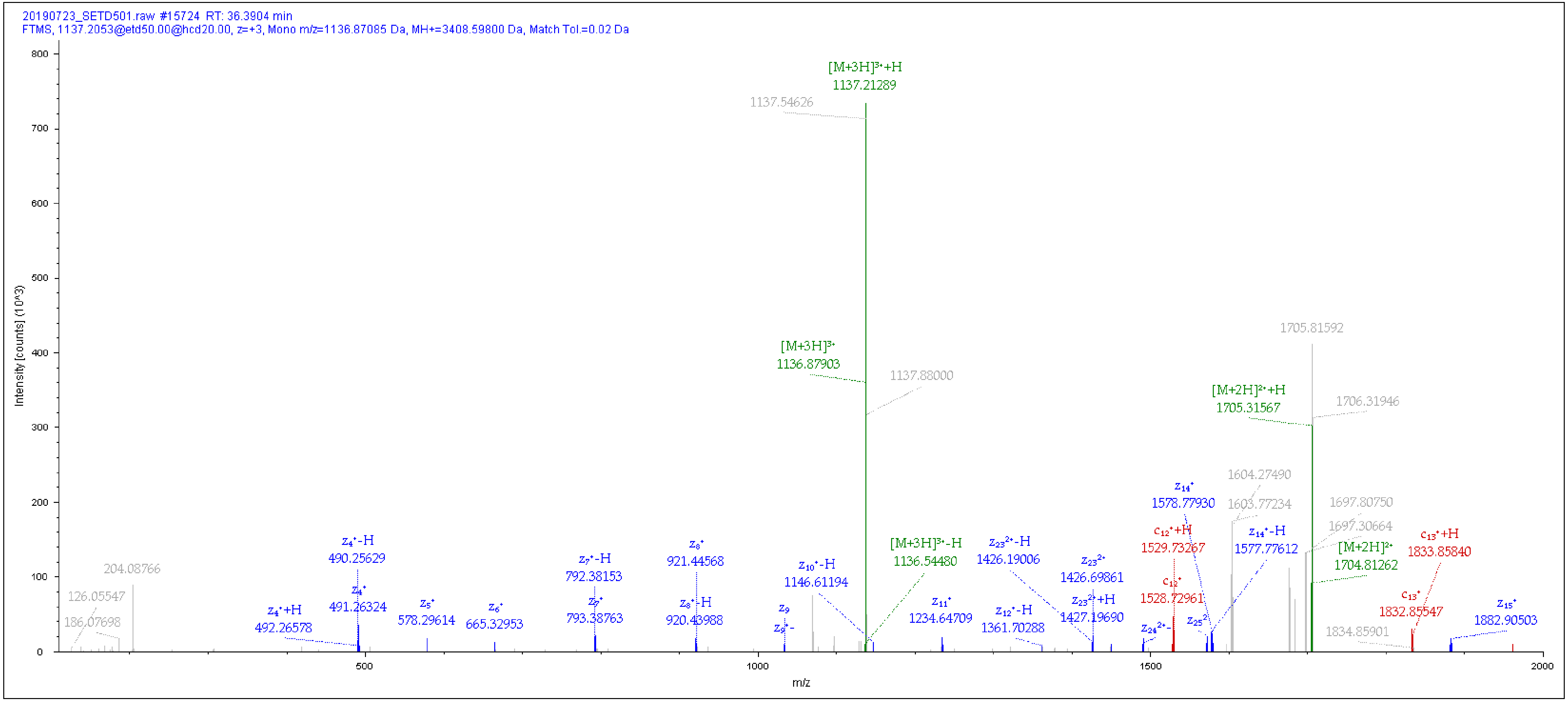

S1251, S1253

EThcD #10586

[M+2H+HexNAc]$^{+2}$= 1051.0037

**TESQSLLQQSSSPFR**

red = O-GlcNAc

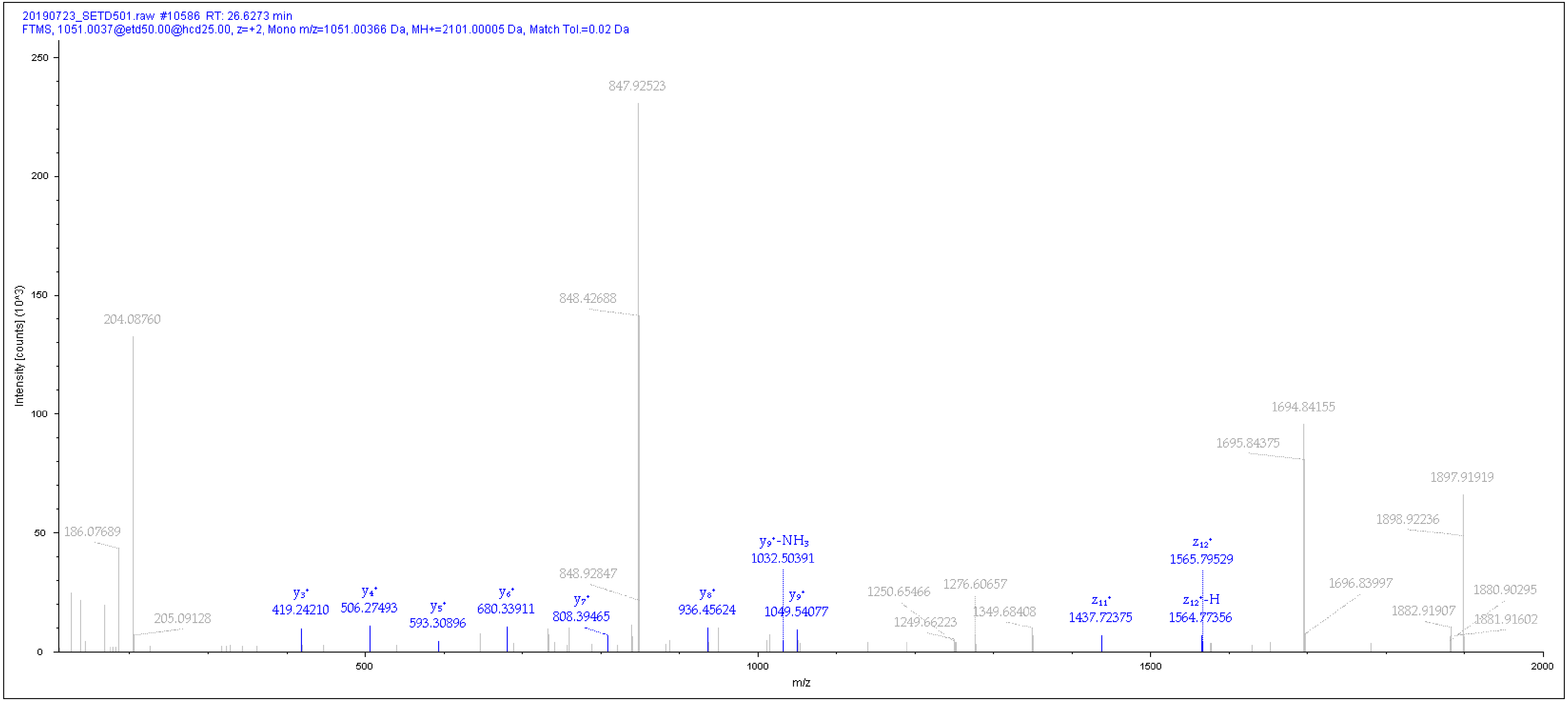

S1258

EThcD #10953

$[M+2H+HexNAc]^{+2}$= 949.4611

**TESQSLLQQSSSPFR**

red = O-GlcNAc

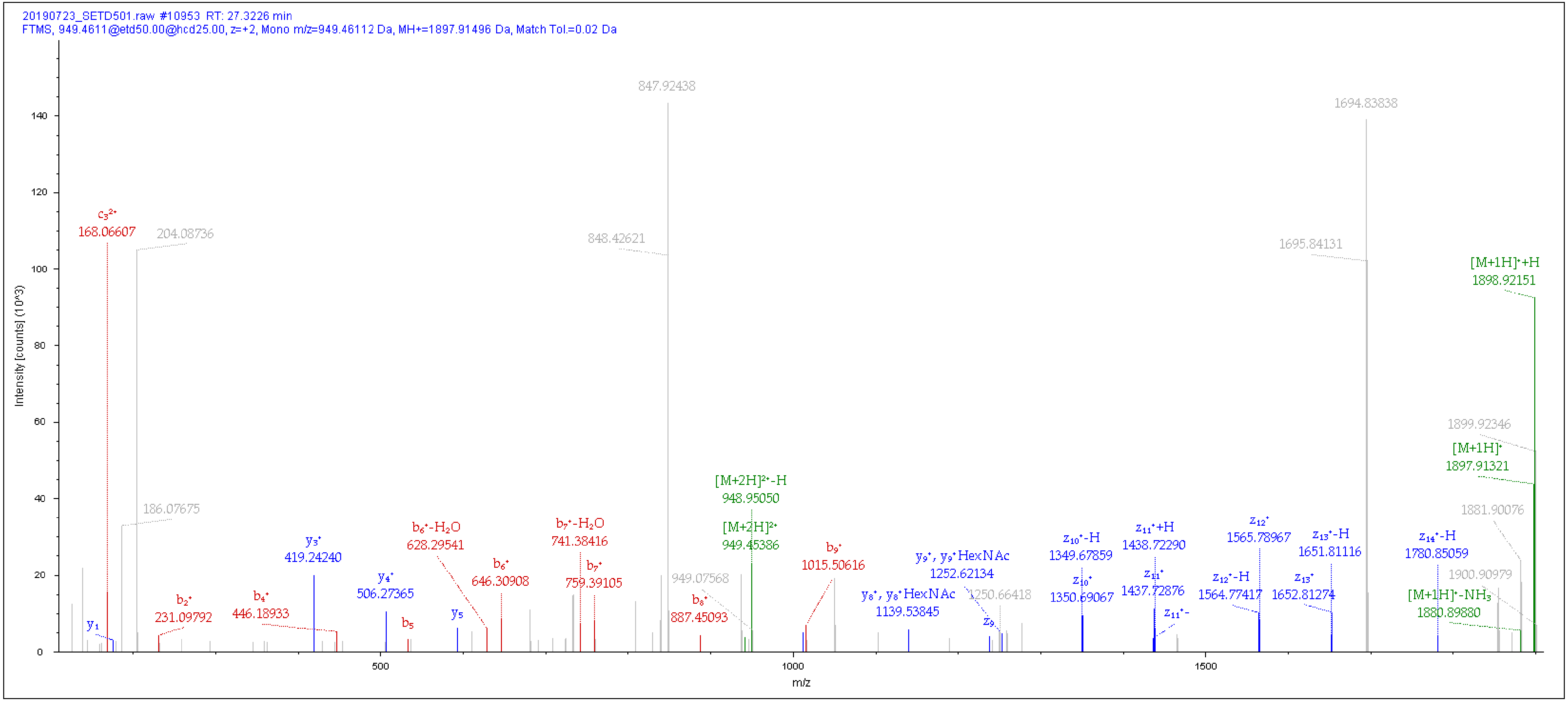

S1365

EThcD #10392

[M+4H+HexNAc]$^{+4}$= 1113.0089

**RSC(carba)PSSAASPTLQGPSDSPTSDSVSQSSTGTLSSTSFPQNSR**

red = O-GlcNAc
carba = Carbamidomethyl

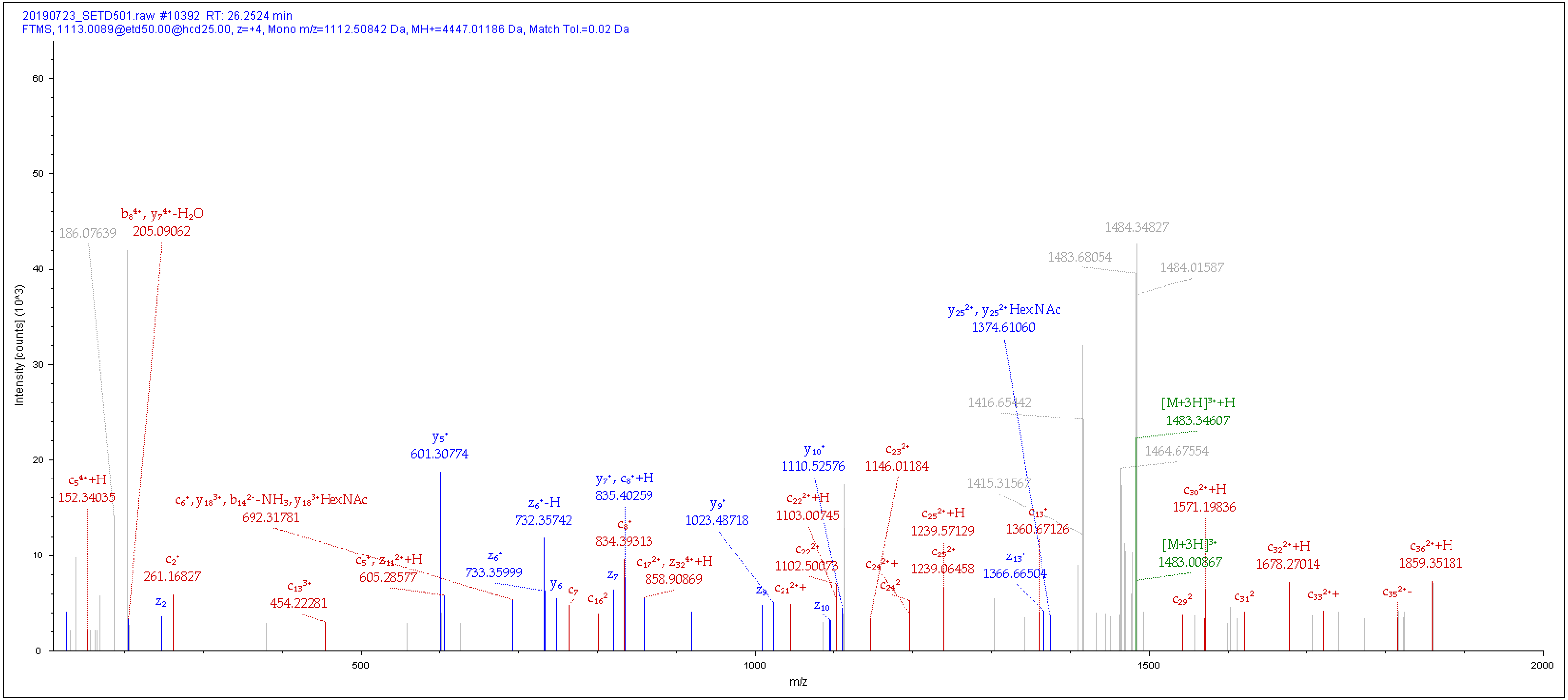

S1380

EThcD #5439

$[M+2H+HexNAc]^{+2} = 539.2749$

**SSLPSDLR**

red = O-GlcNAc

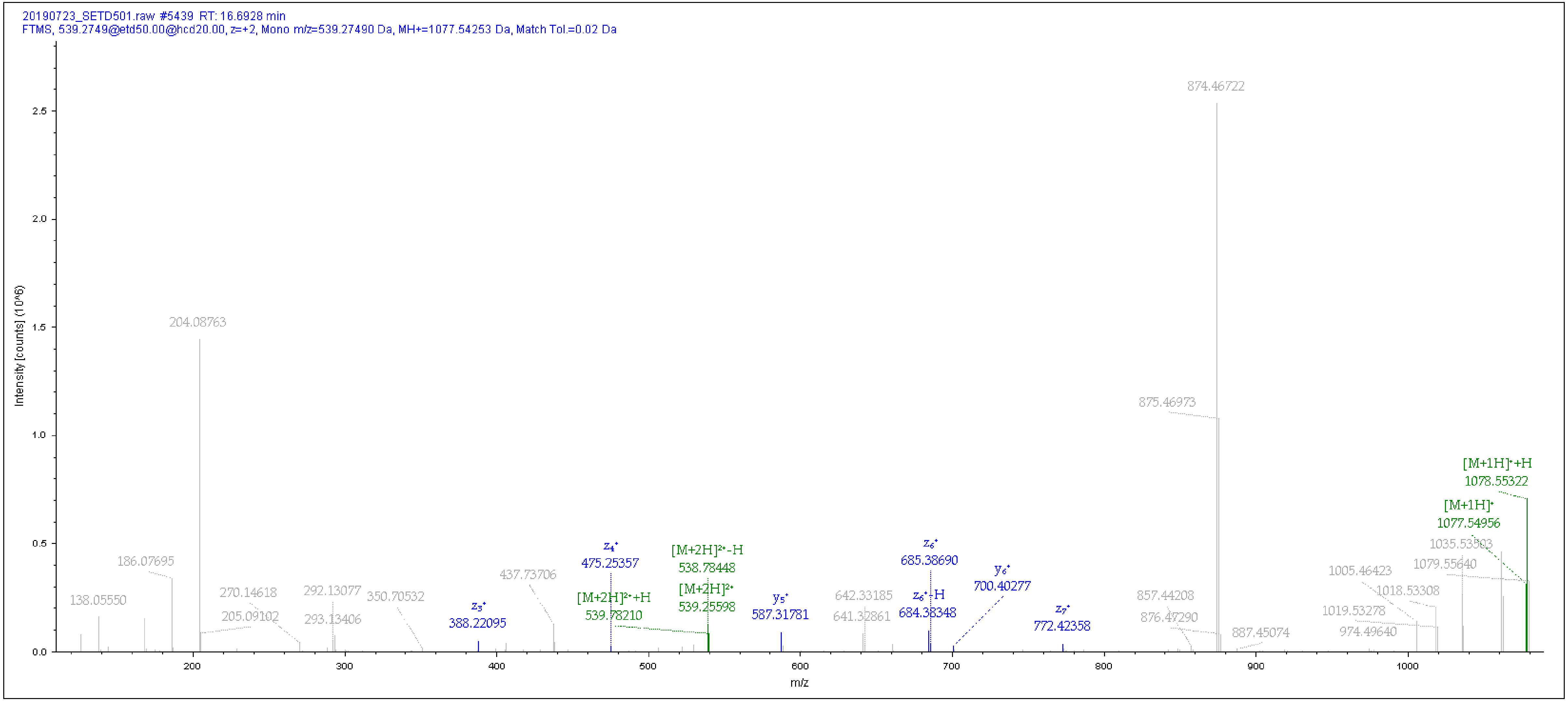

S1381, S1384

EThcD #13680

[M+3H+HexNAc]$^{+3}$= 1000.1733

**SSLPSDLRTISLPSAGQSAVYQASR**

red = O-GlcNAc

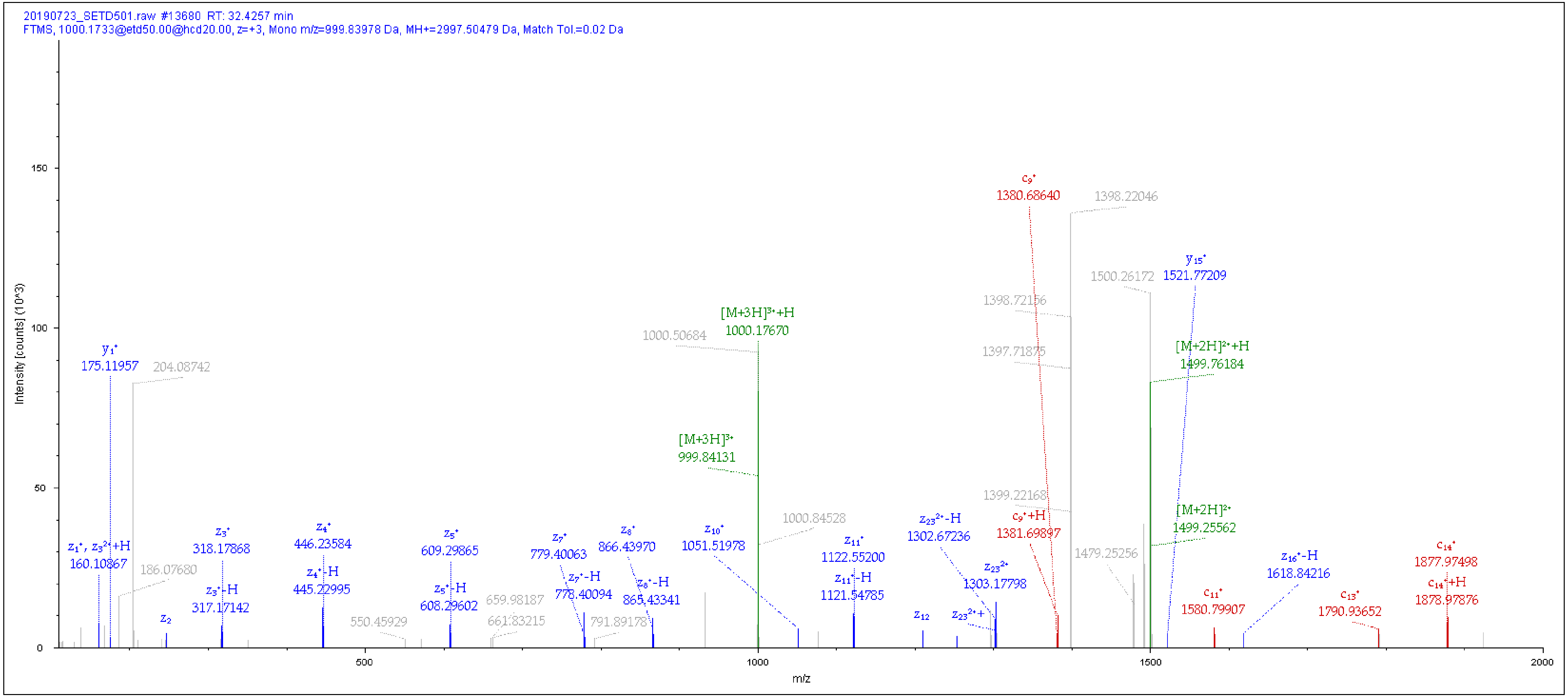

S1388

EThcD #14047

$[M+3H+HexNAc]^{+3} = 932.4807$

**SSLPSDLRTISLPSAGQSAVYQASR**

red = O-GlcNAc

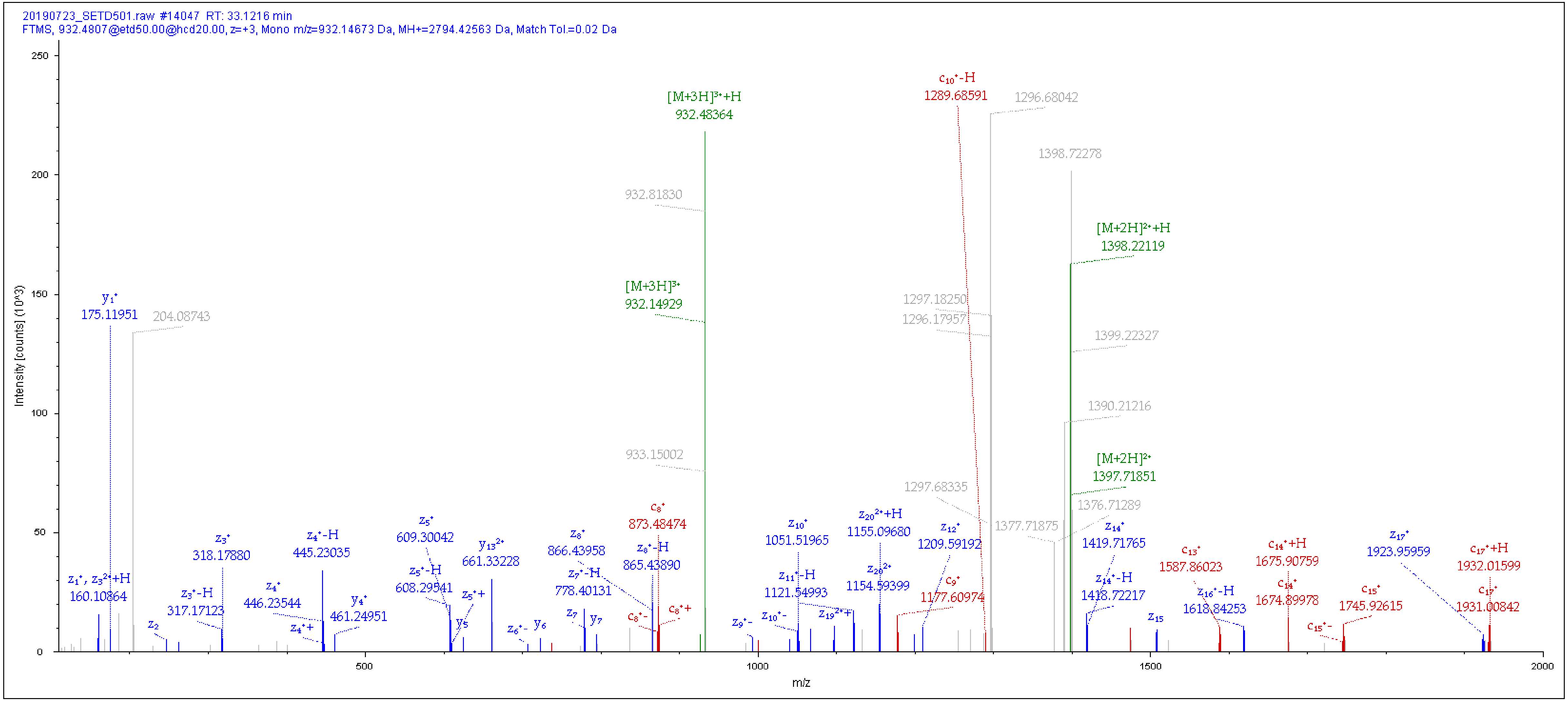

S1390

EThcD #11476

$[M+2H+HexNAc]^{+2}$= 970.4940

**TISLPSAGQSAVYQASR**

red = O-GlcNAc

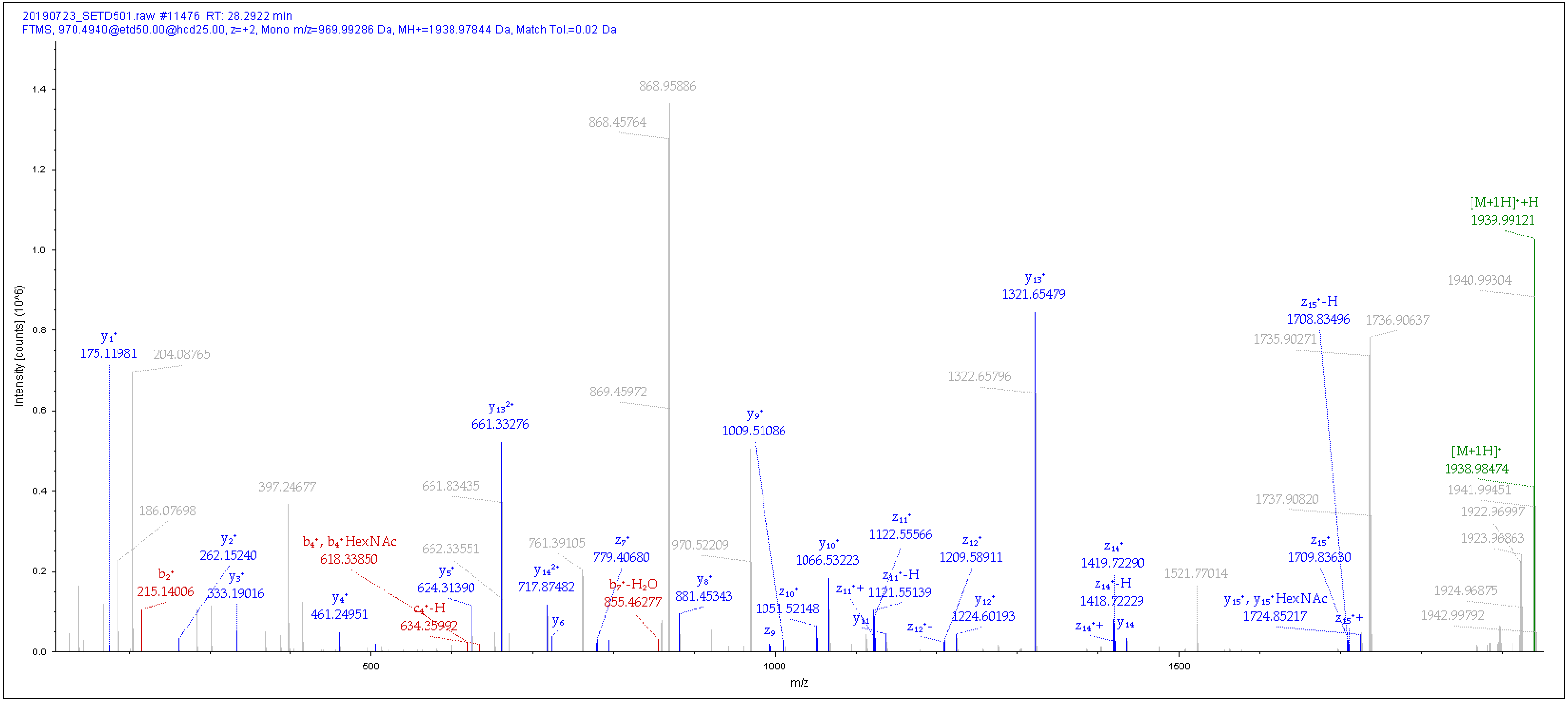

S1406, S1409

EThcD #2606

$[M+3H+HexNAc]^{+3}$= 629.9669

**VSAVSNSQHYPHR**

red = O-GlcNAc

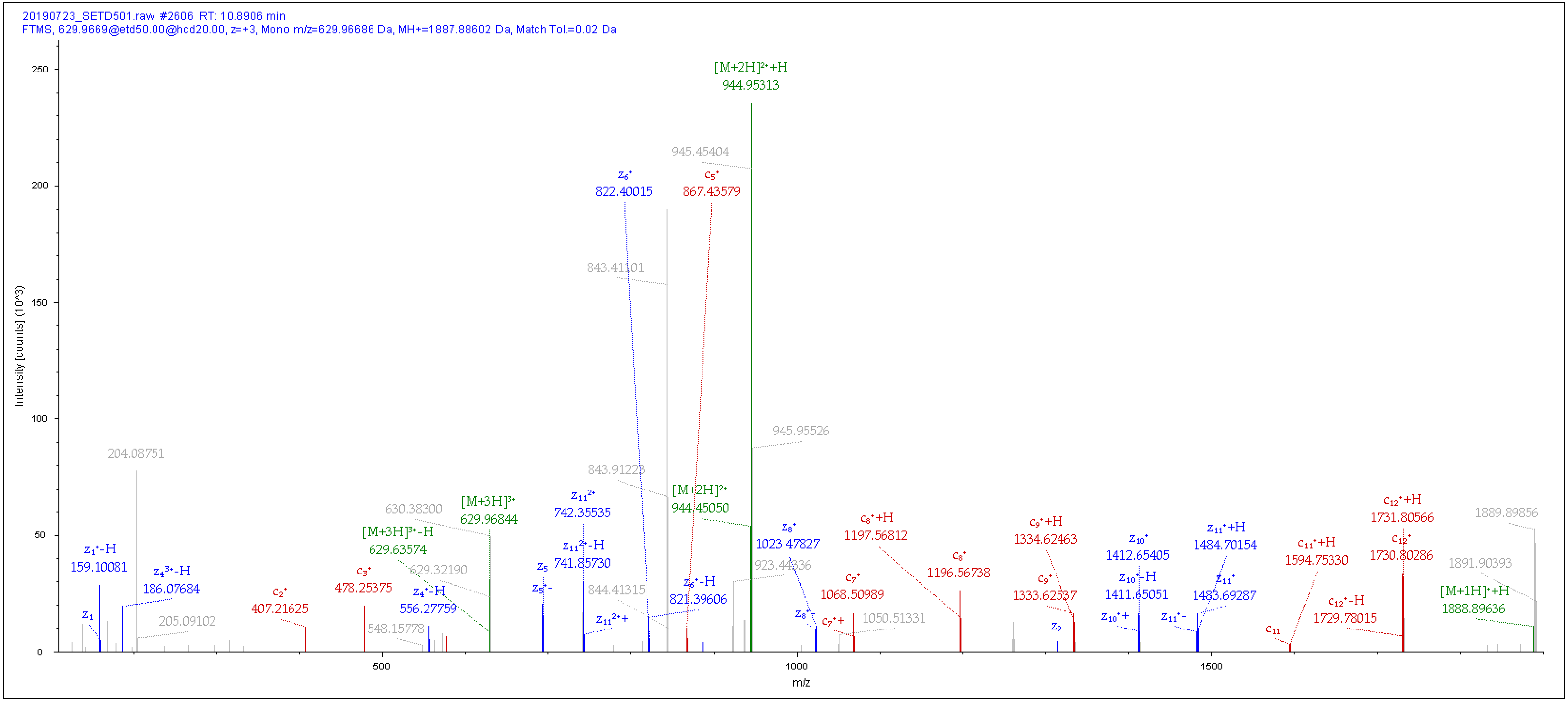

S1409

EThcD #2616

$[M+4H+HexNAc]^{+4}$ = 422.2069

**VSAVSNSQHYPHR**

red = O-GlcNAc

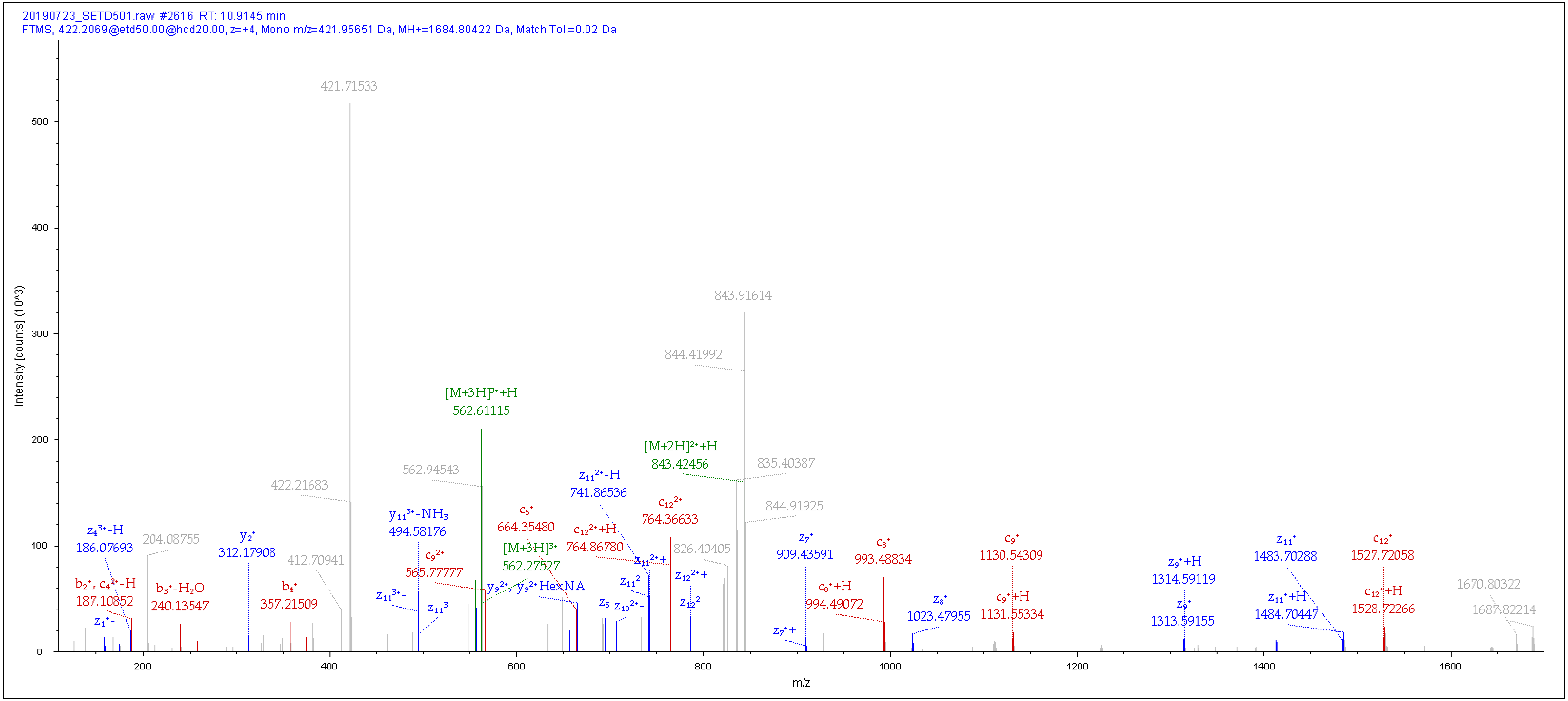

S1433

EThcD #4467

[M+2H+HexNAc]$^{+2}$= 615.3406

**LQPLQGSGVK**

red = O-GlcNAc

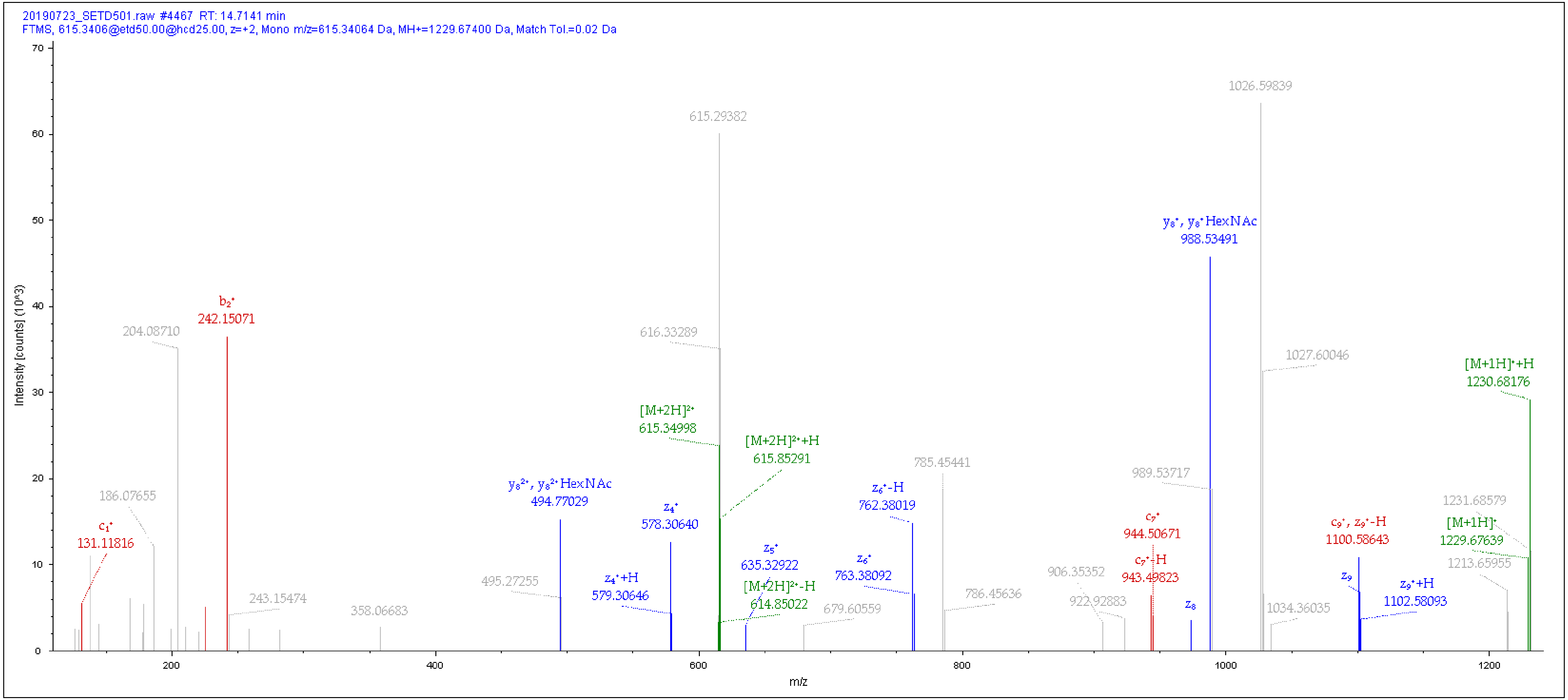

S1437

EThcD #8502

$[M+2H+HexNAc]^{+2}$= 909.4884

**LQPLQGSGVKTQTGLS**

red = O-GlcNAc

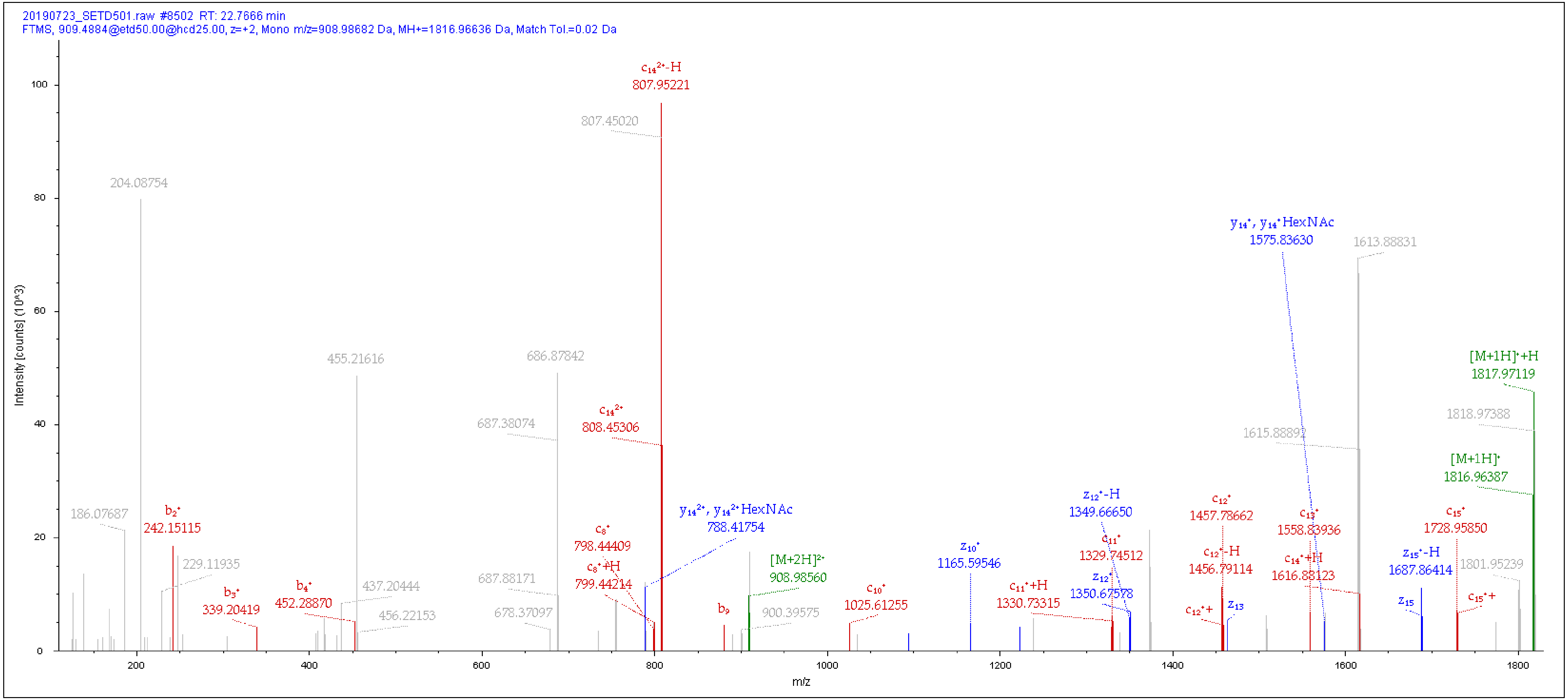

Supplement: Supplementary file 1 — Supplementary Information 1. [file 41598_2023_46923_MOESM1_ESM.pdf]
