# Supplementary material for: SETD5 regulates the OGT-catalyzed O-GlcNAcylation of RNA polymerase II, which is involved in the stemness of colorectal cancer cells
Source: Sci Rep. 2023 Nov 14;13:19885. doi: 10.1038/s41598-023-46923-1 (PMC10646014; doi:10.1038/s41598-023-46923-1)
Supplement: Supplementary file 3 — Supplementary Information 3. [file 41598_2023_46923_MOESM3_ESM.pdf]

Supplementary information

SETD5 regulates the OGT-catalyzed *O*-GlcNAcylation of RNA polymerase II, which is involved in the stemness of colorectal cancer cells

Hye In Cho, Sora Jo, Min Seong Kim, Han Byeol Kim, Xingzhe Liu, Yanhua Xuan, Jin Won Cho, and Yeun Kyu Jang

Inventory

1. **Supplementary Figure S1: relates to Figure 3**
2. **Supplementary Figure S2: relates to Figure 3**
3. **Supplementary Figure S3: relates to Figure 4**
4. **Supplementary Figure S4: relates to Figure 4**
5. **Supplementary Figure S5: relates to Figure 6**
6. **Supplementary Figure S6: relates to Figure 6**
7. **Supplementary Table S1**
8. **Supplementary Table S2**
9. **Supplementary Table S3**
10. **Supplementary Table S4: relates to Figure 1**
11. **Supplementary Table S5: relates to Figure 1**
12. **Supplementary Table S6: relates to Figure 1**
13. **Supplementary Table S7: relates to Figure 1**

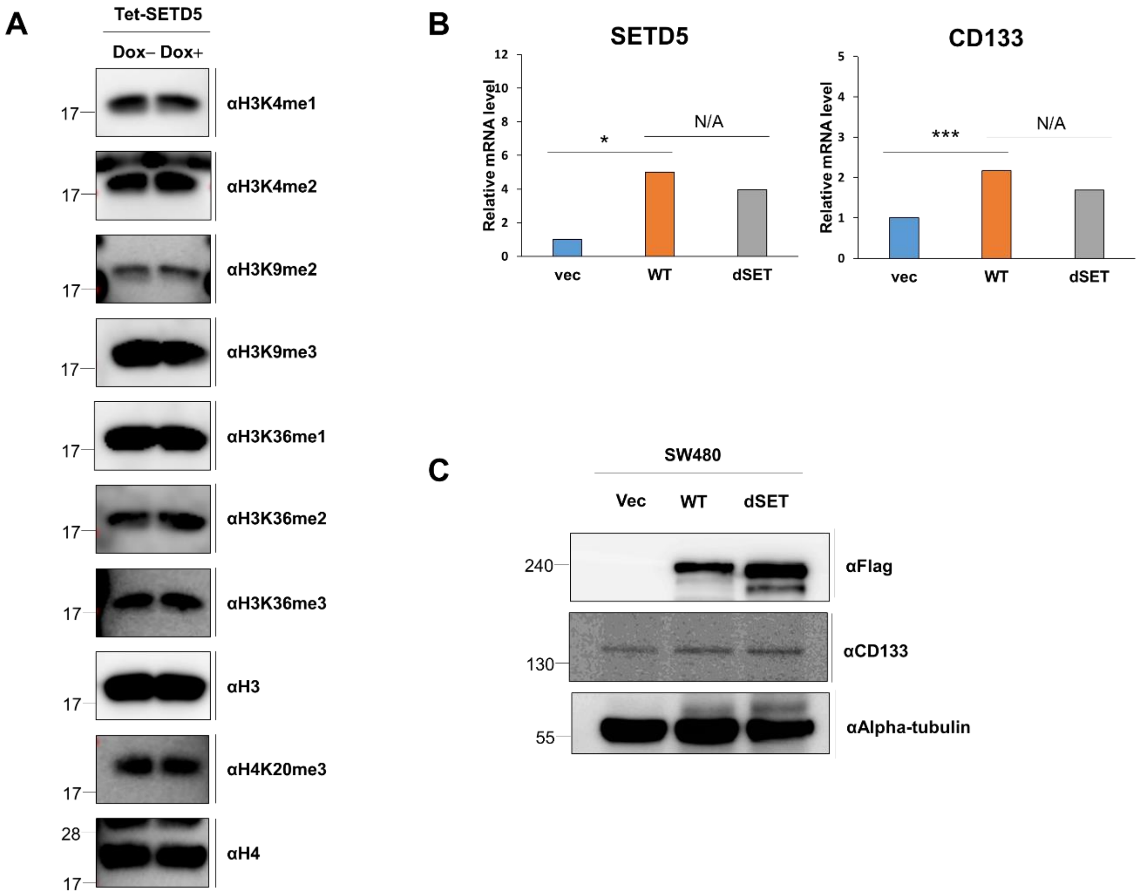

Supplementary Figure S1. The ectopic overexpression of SETD5 little changed the major histone methylations and its SET domain was dispensable for CD133 gene regulation. (A) Western blot analysis of histone methylation levels in Tet-inducible SETD5-based SW480 cells after treatment with 100 ng/ml of doxycycline for 24 h. H3 and H4 were used as loading control. Symbols used: Dox(−), mock treatment without doxycycline; Dox(+), treated with doxycycline. (B) The mRNA level of SETD5 and CD133 in SW480 cells transiently expressing Flag-tagged wild-type (WT) SETD5 or SETD5 deletion mutant (dSET) lacking the SET domain. The CD133 expression levels in the cells expressing the dSET SETD5 mutant were comparable to those of WT-SETD5 overexpressed cells. Flag-tagged WT or dSET SETD5 were transfected into SW480 cells. The mRNA levels were normalized to that of GAPDH. The expression level of the control cells was set as 1. n=3 independent experiments. (C) The protein level of SETD5 and CD133 in SW480 cells transiently expressing Flag-tagged WT SETD5 or dSET SETD5. Flag-tagged WT and dSET SETD5 were transfected into SW480 cells. Alpha-tubulin was used as a loading control. Data are represented as mean ±SEM of triplicate measurements; * $p < 0.05$, ** $p < 0.01$, and *** $p < 0.001$.

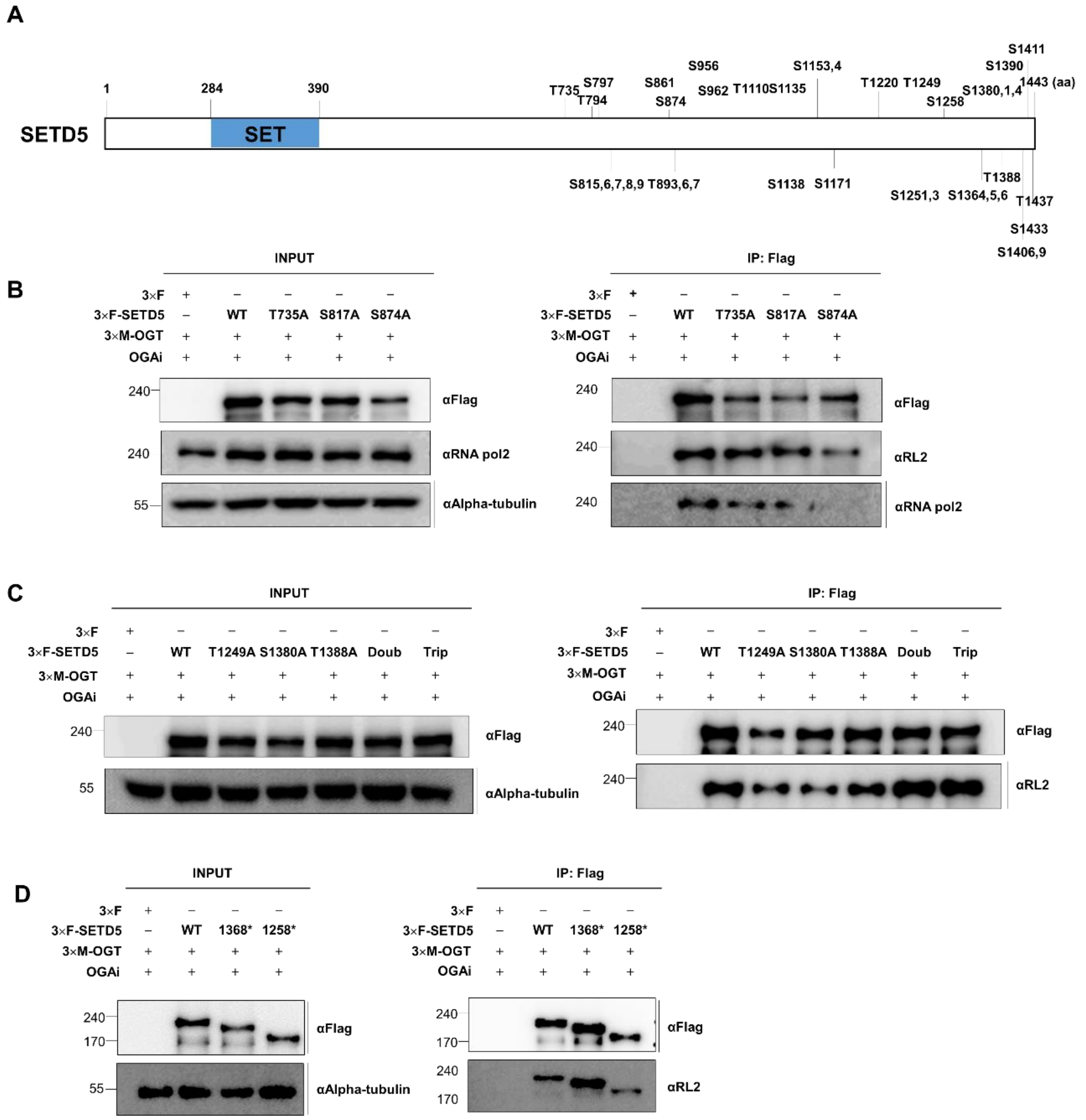

Supplementary Figure S2. Most potential glycosylation sites within the C-terminal region might be required for the SETD5 *O*-GlcNAcylation. (A) The schematic diagram shows the *O*-GlcNAcylation sites of SETD5 as identified by mass spectrometry. (B-C) *In vivo* *O*-GlcNAcylation assay for determining the specific *O*-GlcNAcylation sites within SETD5. Among the 22 sites identified by Mass-spec, six threonine(T)/serine(S) residues (T735, S817, S874, T1249, S1380, T1388) were selected and subjected to site-directed mutagenesis for replacement by alanine. Flag-tagged WT-SETD5 or glycosylation-defective SETD5 mutants were transfected into 293T cells and applied to *in vivo* O-GlcNAcylation assay. The *O*-GlcNAcylation levels of those mutants including double (S1380A/T1388A) and triple (T1249A/S1380A/T1388A) mutants were little changed compared with those of WT-SETD5, suggesting a failure in specifying the SETD5 *O*-GlcNAcylation site(s). Symbols used: 3×F, 3×FLAG-tagged empty vector; 3×F-SETD5, 3×FLAG-tagged-SETD5 plasmid; 3×M-OGT, MYC-tagged OGT plasmid; OGAi, OGA inhibitor; Doub, double mutant (S1380A/T1388A);

Trip, triple mutant (T1249A/S1380A/T1388A); αRL2, *O*-GlcNAc-specific antibody. (D) *In vivo O*-GlcNAcylation assay for determining if the C-terminal deletion mutants (1258* and 1368*) of SETD5 were glycosylation-defective. Their glycosylation levels were still comparable to those of WT-SETD5, suggesting the requirement of the domain (S893~S1258) in the SETD5 glycosylation. Symbols used: 1258*, a deletion mutant form that cannot bind with its binding partners; 1368*, a frameshift mutation form found in ID patients (Deliu et al., 2018).

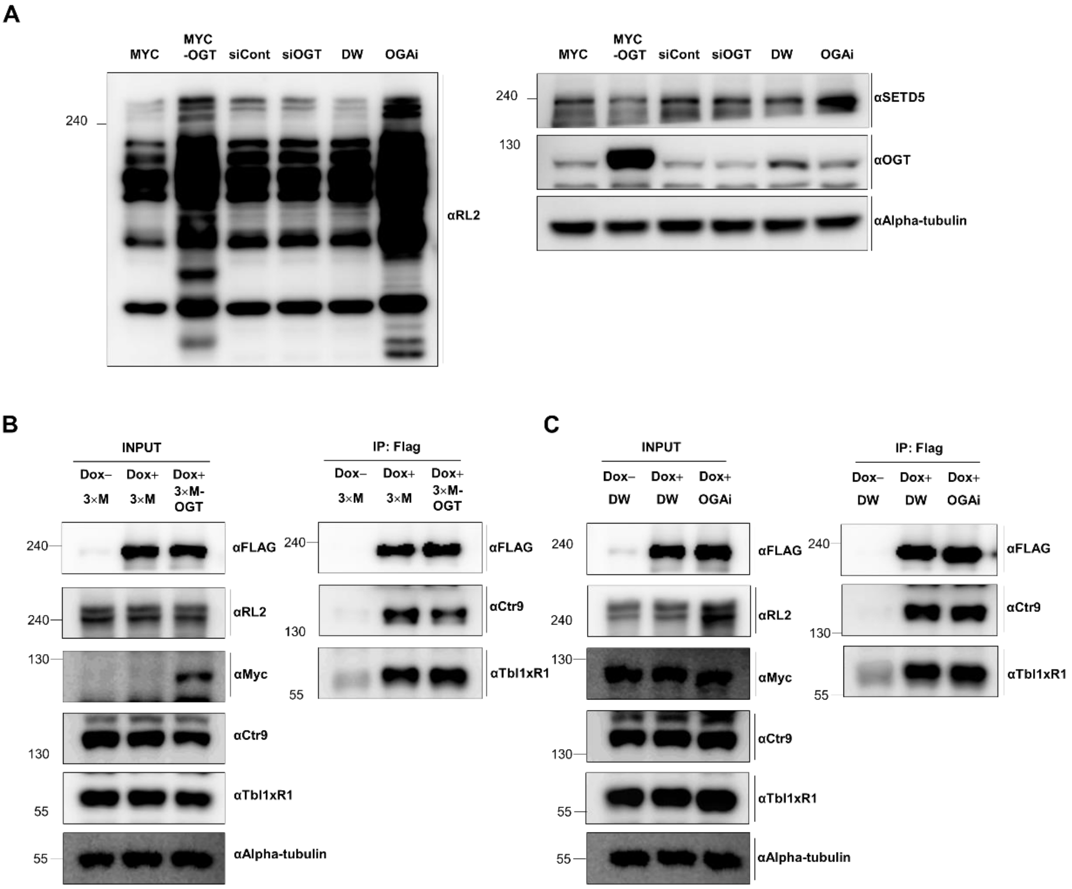

Supplementary Figure S3. The SETD5 *O*-GlcNAcylation does not affect protein stability and its interaction with binding partners. (A) SETD5 protein stability was analyzed by western blot in HCT116 cells with different *O*-GlcNAcylation levels. The enrichment of global *O*-GlcNAcylation in cells was induced by the MYC-tagged OGT overexpression and the treatment with OGA inhibitor. In contrast, the depletion of *O*-GlcNAcylation was caused by treatment with siRNA targeting OGT mRNAs. Symbols used: MYC, MYC-tagged empty vector; MYC-OGT, MYC-tagged OGT plasmid; OGAi, OGA inhibitor; siCont, control siRNAs; siOGT, siRNAs targeting OGT mRNAs; DW, distilled water; αRL2, *O*-GlcNAc-specific antibody. (B) The effect of *O*-GlcNAcylation enrichment on the SETD5-partners interaction was investigated. The interaction between SETD5 and its binding partner proteins (CTR9 and Tbl1xR1) was analyzed by IP assay when the MYC-tagged OGT overexpression in SETD5-overexpressing cells enriched the O-GlcNAcylation. Myc-tagged OGT was transfected into Tet-inducible SETD5-based SW480 cells to induce *O*-GlcNAc enrichment. FLAG-tagged SETD5 was immunoprecipitated by FLAG-affinity gel, and the immunoprecipitates were subjected to western blot for detecting CTR9 and Tbl1xR1. Symbols used: Dox(–), mock treatment without doxycycline; Dox(+), treated with doxycycline. (C) The effect of OGAi-caused *O*-GlcNAcylation enrichment on the SETD5-partners interaction was investigated. The interaction between SETD5 and its binding partner proteins (CTR9 and Tbl1xR1) was analyzed by IP assay when the O-GlcNAcylation was enriched by the treatment of OGA inhibitor (OGAi) in SETD5-overexpressing cells.

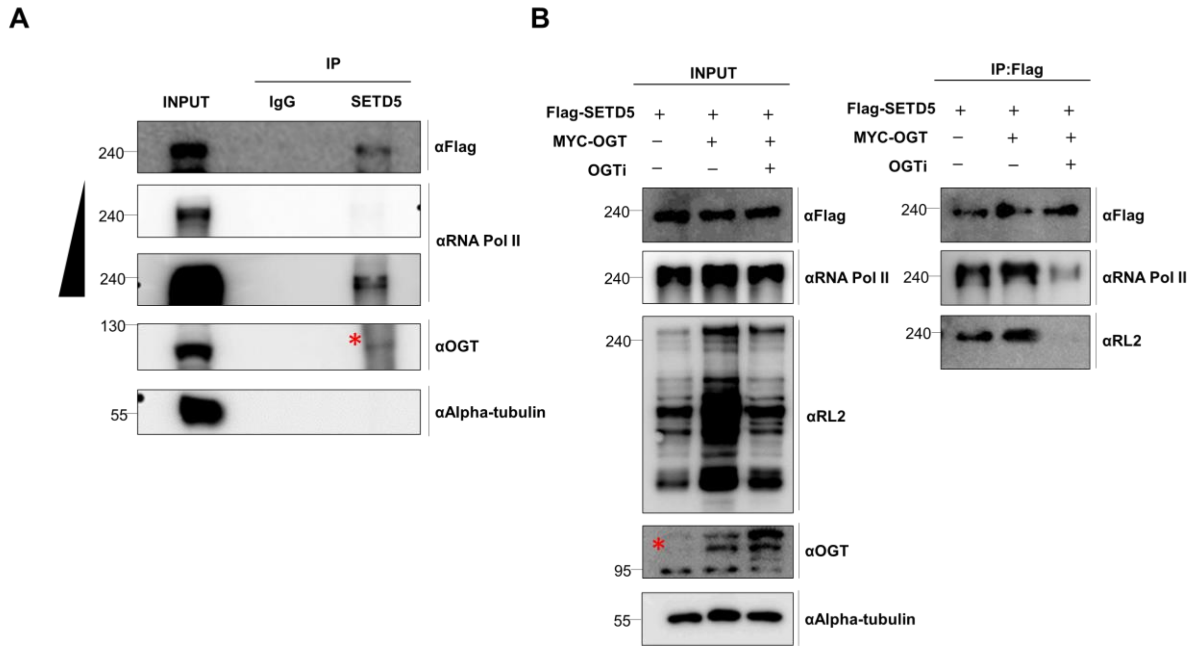

Supplementary Figure S4. SETD5 forms a complex with OGT and RNA polymerase II to mediate the *O*-GlcNAcylation of RNA polymerase II. (A) IP assay using IgG and SETD5 antibody showed that endogenous RNA polymerase II and OGT could form a complex with endogenous SETD5 protein. Symbols used: IgG, Immunoglobulin G. The filled triangle means different exposure times of the western blot image. The asterisk indicates the OGT band. (B) The interaction of SETD5 with RNA polymerase II was analyzed by IP assay in HCT116 cells expressing Flag-tagged SETD5 with MYC-tagged empty vector or MYC-tagged OGT vector. The day after the transfection of indicated vectors, 50µM OSMI-1 (OGT inhibitor) or DMSO (mock treatment) was treated with HCT116 cells for 24 hours. After 24 hours, the cells were harvested. The total protein extracts were subjected to IP assay. Symbols used: Flag-SETD5, Flag-tagged SETD5, MYC-OGT, MYC-tagged OGT, OGTi, OGT catalytic inhibitor, αRL2, O-GlcNAc-specific antibody. The asterisk indicates the OGT band.

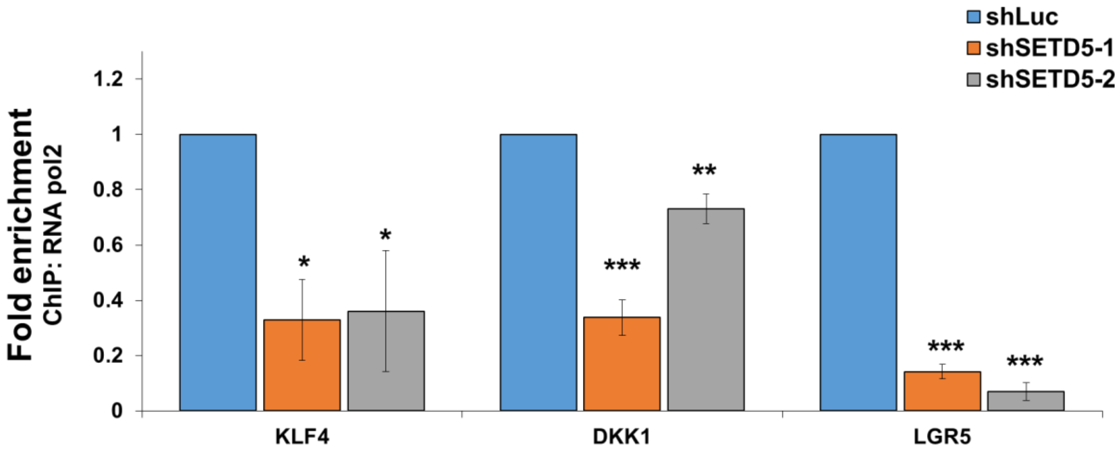

Supplementary Figure S5. The RNA polymerase II occupancy was decreased in the promoter of the WNT target genes under the *SETD5* depletion. RNA polymerase II enrichment in promoters of WNT target genes (*KLF4, DKK1*, and *LGR5*) was investigated by ChIP analysis in *SETD5*-depleted HCT116 cells. The enrichment level of the control cells was set as 1. n=3 independent experiments. Data are represented as mean ±SEM of triplicate measurements; * $p < 0.05$, ** $p < 0.01$, and *** $p < 0.001$. Symbols used: shLuc, shRNAs targeting luciferase mRNAs, shSETD5, shRNAs targeting SETD5 mRNAs.

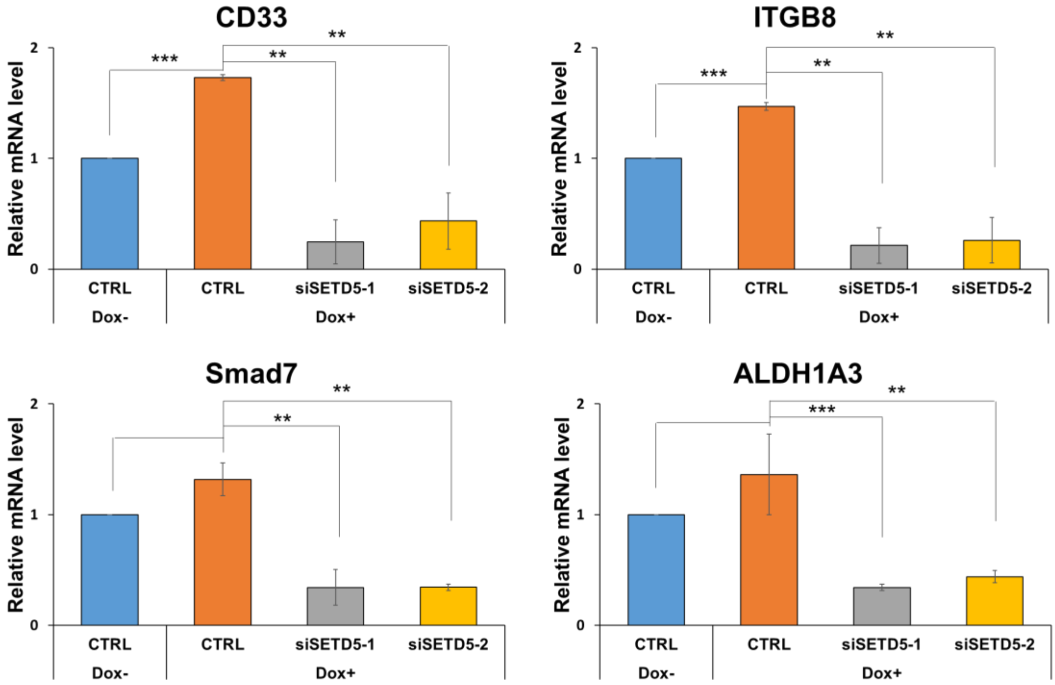

Supplementary Figure S6. The increased expression of PI3K-AKT pathway-related genes in the SETD5-overexpressed CRC cells was nullified by the *SETD5* depletion. The mRNA levels of PI3K-AKT pathway-related genes (*CD33, ITG88, Smad7*, and *ALDH1A3*) were examined by qRT-PCR when the *SETD5* was depleted by siSETD5s in the SETD5-overexpressed SW480 cells. The Dox-inducible overexpression of SETD5 and siRNA-based *SETD5* depletion were done the same in (A-B) of Fig. 6. n=3 independent experiments. Symbols used: Dox(−), mock treatment without doxycycline; Dox(+), treated with doxycycline.

Supplementary Table S1. Primer sequences used for cloning

Gene	Primer sequence (5’ to 3’)
SETD5 WT	F:GCGGATATCATGAGCATTGCAATCCCT<br>R:ATAGTCGACCTAGGAAAGTCCCGTCTG
SETD5 N-term	F:GCGGATATCATGAGCATTGCAATCCCT<br>R:ATAGTCGACCTA ATGAGCTAGGTTCTCCTG
SETD5 Mid-term	F:AGAAAGCTTAGCAGGAGGACCAGGGAA<br>R:AGAGTCGACCTATGGTGAAAACATGAGGCT
SETD5 C-term	F:GCGAAGCTTTCACATCTCTTACTACTGC<br>R:ATAGTCGACCTAGGAAAGTCCCGTCTG
SETD5 1368*	F:GCGGATATCATGAGCATTGCAATCCCT<br>R:ATAGTCGACTCAAGTTCCTGTGCTGGACTG
SETD5 1258*	F:GCGGATATCATGAGCATTGCAATCCCT<br>R:ATAGTCGACTCAACTCTGCTGAAGGAGGCT
SETD5 △C	F:GCGGATATCATGAGCATTGCAATCCCT<br>R:ATAGTCGACTCAACTGGTGAAAACATGAGG
SETD5 dSET	F:TTGGGAACCAATAACTGT<br>R:AATTATAAAGTGGACTGT
SETD5 T735A	F:ACTGTACTGGCAACGGCCCTAAACATGTTAC<br>R:GTAACATGTTTAGGGCCGTTGCCAGTACAGT
SETD5 S817A	F: AATGAGAATAGTAGCGCTTCTAGTATCTGCA<br>R: TGCAGATACTAGAAGCGCTACTATTCTCATT
SETD5 S874A	F:GCCACACCTGGCTCAGCTCACCCAGGAGAAG<br>R:CTTCTCCTGGGTGAGCTGAGCCAGGTGTGGC
SETD5 T1249A	F:TGTGATAGTCCTCGGGCAGAATCACAAAGC C<br>R:GGCTTTGTGATTCTGCCCGAGGACTATCACA
SETD5 S1380A	F:CCTCAGAACTCTAGGGCGTCATTGCCATCAG<br>R:CTGATGGCAATGACGCCCTAGAGTTCTGAGG
SETD5 T1388A	F:CCATCAGACTTACGGGCTATCAGTCTGCCC A<br>R:TGGGCAGACTGATAGCCCGTAAGTCTGATGG
SETD5 S1380A/T1388A(Dou)	F:CCTCAGAACTCTAGGGCGTCATTGCCATCAGACTTACGGGCTATCAGTCTGCCCA<br>R:TGGGCAGACTGATAGCCCGTAAGTCTGATGGCAATGACGCCCTAGAGTTCTGAGG

Supplementary Table S2. Primer sequences used for real-time PCR

Gene	Primer sequence (5' to 3')
GAPDH	F :TGATGACATCAAGAAGGTGGTGAAG<br>R : TCCTTGGAGGCCATGTGGGCCAT
LGR5	F : CCTGTCCTTGCCTGTGCT<br>R : CCACCCTGAGCAACATCC
SETD5	F:GCGGTAAGCCCATCAGATT<br>R:ATGTGGGAACCATCCACCT
CD133	F : TGCAGTGGATCGAGTTCT<br>R : TCCTATGCCAAACCAAAA
AKT1	F:CCGTGTGTGGACGAATGC<br>R:AGGTAGGGACCGGAGAGC
AKT2	F:TCAGTGTGTTTGGGTTGG<br>R:AAGGGTGGGGGAAGGAAG
AKT3	F:CCGGAAAGATTGTGTACCG<br>R:CTTCATGGTGGCTGCATCT
KLF4	F:CTTCGTGAACATTAACGACAG GGCC<br>R:AATGGCCACCCTGACTAAGGAGTGG
ESRRB	F:AGAGAGCAGCCCATACCTGA<br>R:AGGCATGGCATAGAGCTTGT
DKK1	F:CATCAGACTGTGCCTCAGGA<br>R:TATCCGGCAAGACAGACCTT
CD33	F:GCCCTGCACTTTCTTCCAT<br>R:CTTGTTTGTGGCCACTGGA
FXYD3	F:ATGGGAAGGGGGTATAGTGG<br>R:TCGCTGGGCTGAGAGATAA
ALDH1A3	F:TCTCGACAAAGCCCTGAAGT<br>R:GGCCAAAGCGTATTCACCTA
SMAD7	F:TCATCAAGTCCGCCACACT<br>R:CACGGCTGCTGCATAAACT
ITGB8	F:CAAAGGCTGCAAACCTCAA<br>R:TTCTGGACCCATCTGGACA

Supplementary Table S3. Primer sequences used for ChIP

Gene	Primer sequence (5' to 3')
CD44	F :TGATGACATCAAGAAGGTGGTGAAG<br>R : TCCTTGGAGGCCATGTGGGCCAT
AKT2	F:TGCTCCAAGCAGACAGATGGG<br>R:TTTTACCTATTCTACCTCCTTGGTG
AKT3	F:CTTCGTGAACATTAACGACAGGGCC<br>R:AATGGCCACCCTGACTAAGGAGTGG
CD133	F:GGATGCTGTCCAGGTGCT<br>R:TGGGGATCTGCCTCAGTC
FXYD3	F:CAGACAGTTCTCATTCTC<br>R:GCCGCTGGTACAATTTCA
CD33	F:TGAGCACGTGTGGGTCTG<br>R:GTCTGAGGCAGAGGCTTC
ALDH1A3	F:CAGCAAAGGTCTCATGTGCTT<br>R:GGGGGCAATTGATCTGATTT
ITGB8	F:TGCCCCTAGAGGGAAAAGA<br>R:TCGGGTCCTGTCATTACCA
SMAD7	F:AGCACCTGCATCTCCCTTT<br>R:CTGTTGGCCATTGGTTGAG
KLF4	F: TTCCCATGGCAGGACTTTCAA<br>R: ATGGCAGCTAAATAAACAAACT
DKK1	F: AGAGCCTATCACCCCTCGG<br>R: TGGGAGGGAGACAACAAAGC
LGR5	F: AGGTCTGGTGTGTTGCTGAG<br>R: GAGTGACGTGGGGAAGTACT

Supplementary Table S4. SETD5 protein expression in colorectal tissue

Diagnosis	n	SETD5 (-) n(%)	SETD5 (+) n(%)	χ^2	R	P-value
				4.319	0.172	0.115
**Hyperplastic polyps**	34	17(50.0)	17(50.0)			
**Tubular adenoma**	9	3(33.3)	6(66.7)			
**Carcinoma**	98	30(30.6)	68(69.4)			

Supplementary Table S5. Comparison of clinicopathologic characteristics according to SETD5 expression in colorectal cancer tissues

Variable	n	SETD5 (-) n(%)	SETD5 (+) n(%)	χ^2	R	P-value
**Age(years)**				2.790	0.166	0.095
**≤60**	45	9(20.0)	36(80.0)			
**>60**	53	19(35.8)	34(64.2)			
**Sex**				0.098	0.031	0.755
**Male**	58	16(27.6)	42(72.4)			
**Female**	40	12(30.0)	28(70.0)			
**Grade**				3.343	0.183	0.329
**Well**	29	9(31.0)	20(69.0)			
**Moderately**	61	19(31.1)	42(68.9)			
**Poorly**	3	0(0)	3(100.0)			
**Mucinous**	5	0(0)	5(100.0)			
**Multiple**				2.431	0.159	0.119
**Negative**	82	22(26.8)	60(73.2)			
**Positive**	16	7(43.7)	9(56.3)			
**Location**				2.622	0.161	0.623
**A-colon**	3	1(33.3)	2(66.7)			
**T-colon**	9	4(44.4)	5(55.6)			
**S-colon**	22	7(31.8)	15(68.2)			
**Rectum**	61	16(26.2)	45(73.8)			
**Anus**	3	0(0.0)	3(100.0)			
**T stage**				0.369	0.061	0.544
**T1-2**	11	4(36.4)	7(63.6)			

**T3-4**	87	24(27.6)	63(72.4)			
**Lymph node metastasis**				0.038	0.020	0.845
**Negative**	58	17(29.3)	41(70.7)			
**Positive**	40	11(27.5)	29(72.5)			
**Distant metastasis**				4.919	0.219	0.027
**Negative**	68	24(35.3)	44(64.7)			
**Positive**	30	4(13.3)	26(86.7)			
**Clinical stage**				5.039	0.221	0.169
**1**	9	3(33.3)	6(66.7)			
**2**	38	13(34.2)	25(65.8)			
**3**	21	8(38.1)	13(61.9)			
**4**	30	4(13.3)	26(86.7)			
**Radiotherapy**				5.188	0.224	0.023
**Negative**	81	27(33.3)	54(66.7)			
**Positive**	17	1(5.9)	16(94.1)			
**Chemotherapy**				2.673	0.163	0.102
**Negative**	21	9(42.9)	12(57.1)			
**Positive**	77	19(24.7)	58(75.3)			

Supplementary Table S6. Univariate and Multivariate analyses for prognostic variables of overall survival in colorectal cancer patients using Cox proportional-hazards regression

Characteristic	Univariate analyses			Multivariate analyses		
	HR	95% CI	p-value	HR	95% CI	p-value
**Age (years)**			0.502			0.530
**≤60**	1.00		-	1.00		-
**>60**	1.275	0.627-2.592		1.247	0.625-2.489	
**T stage**			0.976			0.144
**T1-2**	1.00		-	1.00		-
**T3-4**	200350.304	0.000-3.298		25.305	0.331-1935.418	
**Lymph node metastasis**			0.010			< 0.001
**Negative**	1.00		-	1.00		-
**Positive**	2.825	1.281-6.227		4.198	1.989-8.862	
**Distant metastasis**			< 0.001			< 0.001
**Negative**	1.00		-	1.00		-
**Positive**	13.740	5.184-36.419		21.021	8.319-53.117	
**SETD5**			0.128			0.012
**Negative**	1.00		-	1.00		-
**Positive**	2.630	0.757-9.141		4.620	1.409-15.152	

Supplementary Table S7. Univariate and Multivariate analyses for prognostic variables of disease-free survival in colorectal cancer patients using Cox proportional-hazards regression

Characteristic	Univariate analyses			Multivariate analyses		
	HR	95% CI	p-value	HR	95% CI	p-value
**Age (years)**			0.809			0.974
**≤60**	1.00		-	1.00		-
**>60**	0.921	0.471-1.800		0.989	0.518-1.889	
**T stage**			0.969			0.118
**T1-2**	1.00		-	1.00		-
**T2-4**	200168.316	0.000-3.17		25.490	0.442-1471.368	
**Lymph node metastasis**			0.018			< 0.001
**Negative**	1.00		-	1.00		-
**Positive**	2.365	1.161-4.815		3.378	1.715-6.653	
**Distant metastasis**			< 0.001			< 0.001
**Negative**	1.00		-	1.00		-
**Positive**	7.532	3.349-16.938		11.439	5.353-24.445	
**SETD5**			0.041			0.005
**Negative**	1.00		-	1.00		-
**Positive**	3.547	1.051-11.973		5.527	1.696-18.015	
