# Supplementary material for: SETD5 regulates the OGT-catalyzed O-GlcNAcylation of RNA polymerase II, which is involved in the stemness of colorectal cancer cells
Source: Sci Rep. 2023 Nov 14;13:19885. doi: 10.1038/s41598-023-46923-1 (PMC10646014; doi:10.1038/s41598-023-46923-1)
Supplement: Supplementary file 4 — Supplementary Information 4. [file 41598_2023_46923_MOESM4_ESM.pdf]

Fig2A

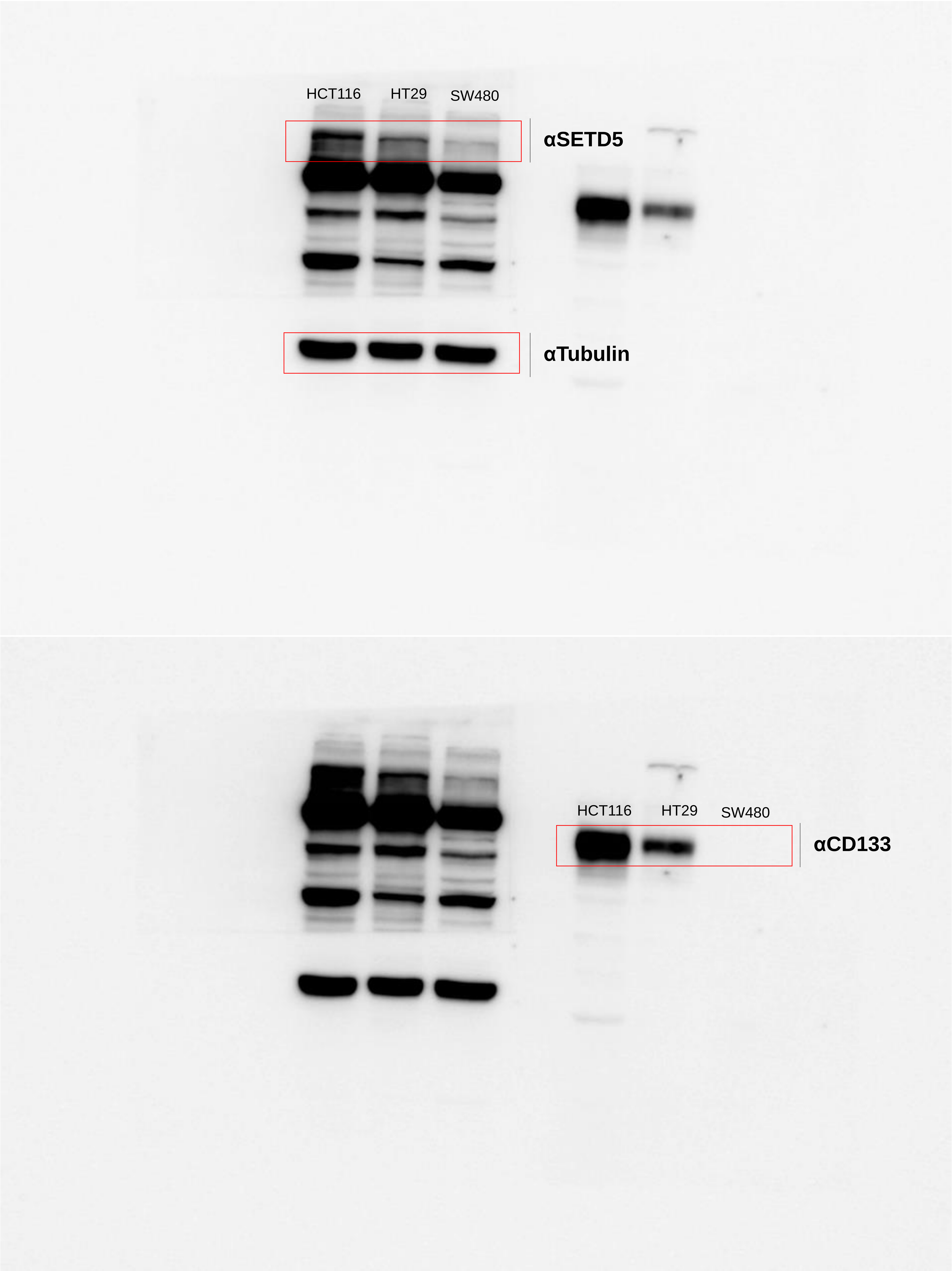

HCT116
HT29
SW480
αSETD5
αTubulin
HCT116
HT29
SW480
αCD133

Fig2C

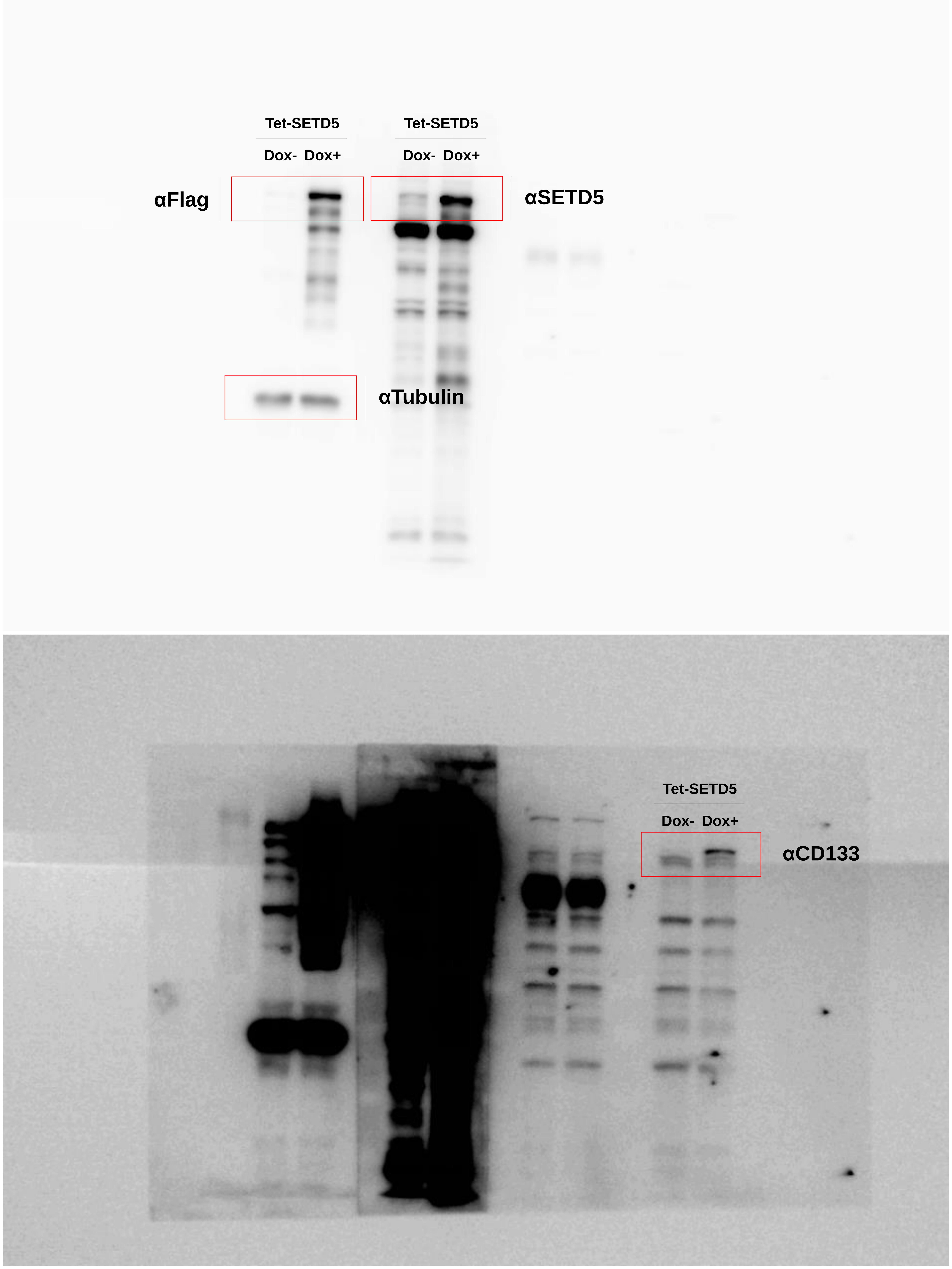
Tet-SETD5
Dox- Dox+
Tet-SETD5
Dox- Dox+
αFlag
αSETD5
αTubulin
Tet-SETD5
Dox- Dox+
αCD133

Fig2E

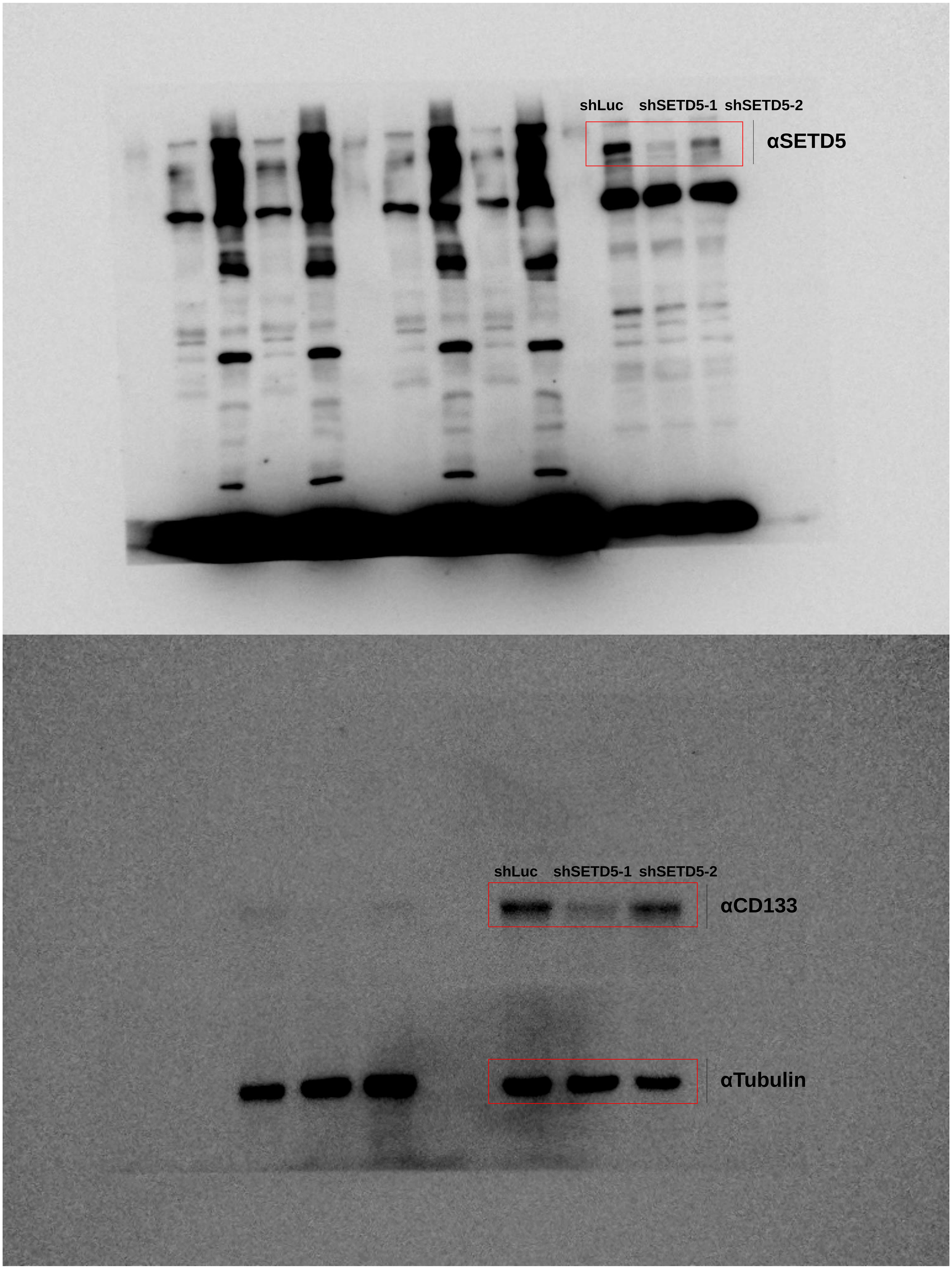
shLuc shSETD5-1 shSETD5-2
αSETD5
shLuc shSETD5-1 shSETD5-2
αCD133
αTubulin

Fig3A

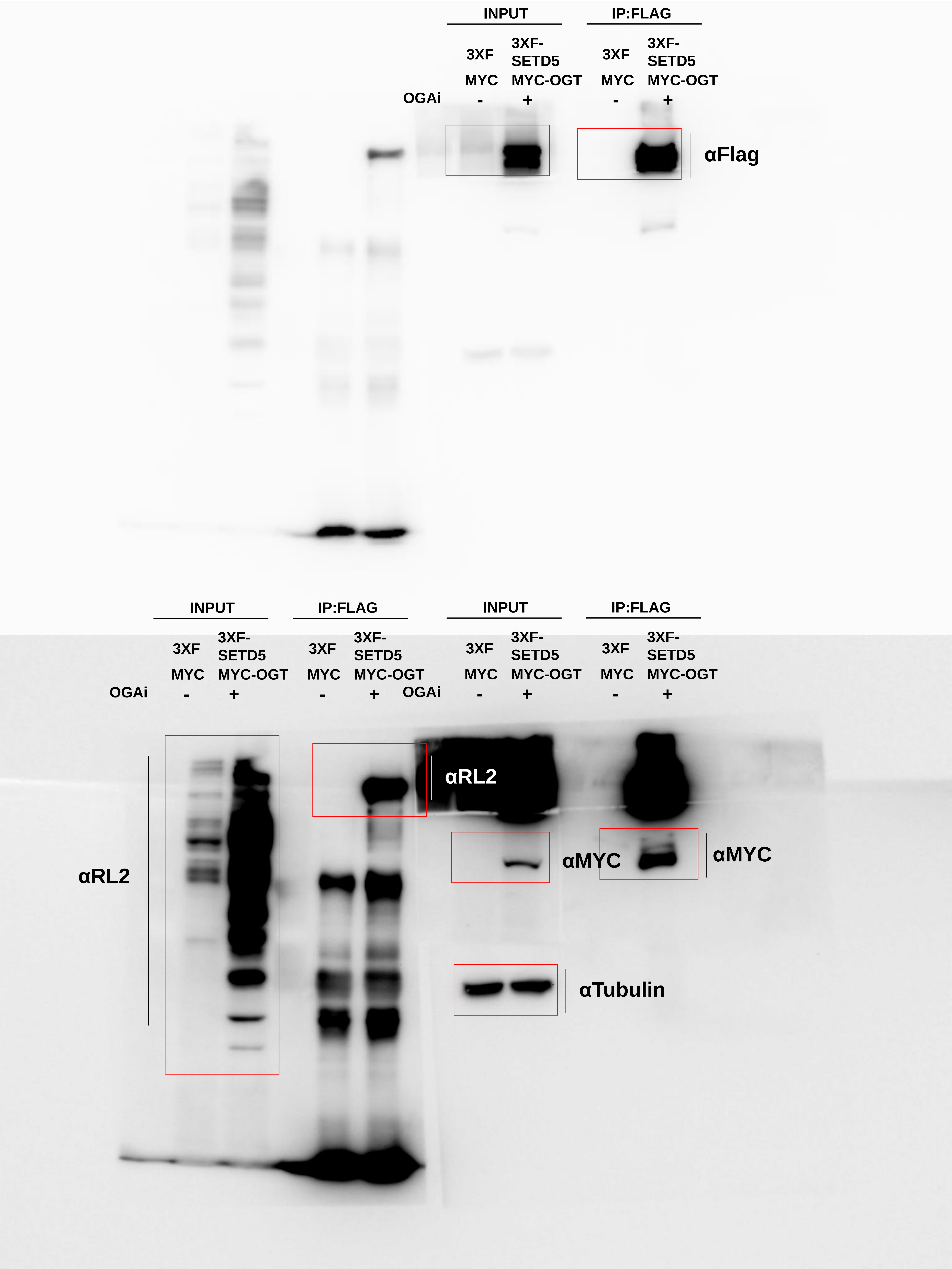
INPUT
IP:FLAG
3XF
MYC
3XF-
SETD5
MYC-OGT
OGAi
-
+
αFlag
INPUT
IP:FLAG
INPUT
IP:FLAG
OGAi
αRL2
αRL2
αMYC
αMYC
αTubulin

Fig3B-1

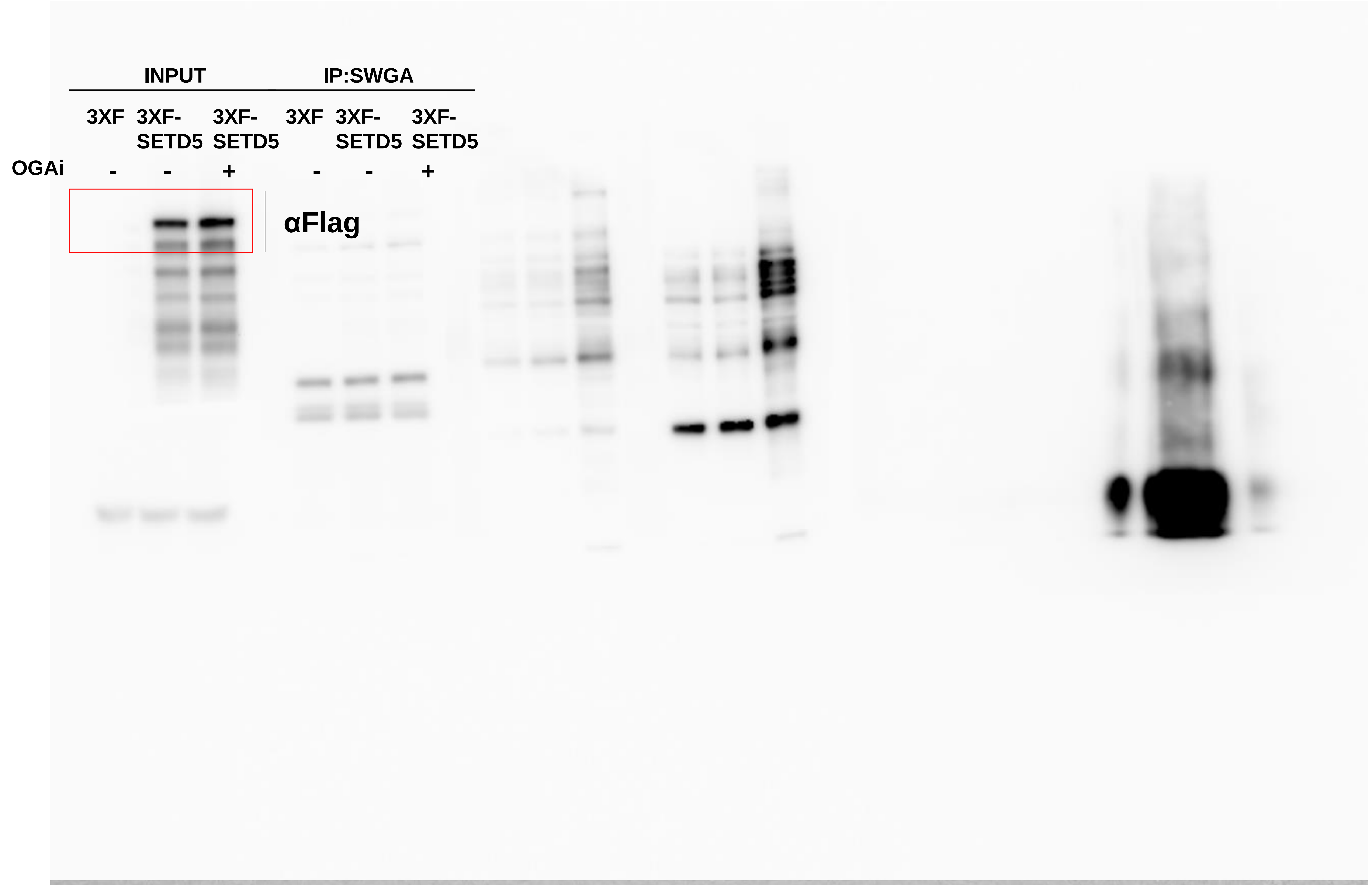
INPUT
IP:SWGA
3XF
3XF-SETD5
3XF-SETD5
3XF
3XF-SETD5
3XF-SETD5
OGAi
-
-
+
-
-
+
αFlag

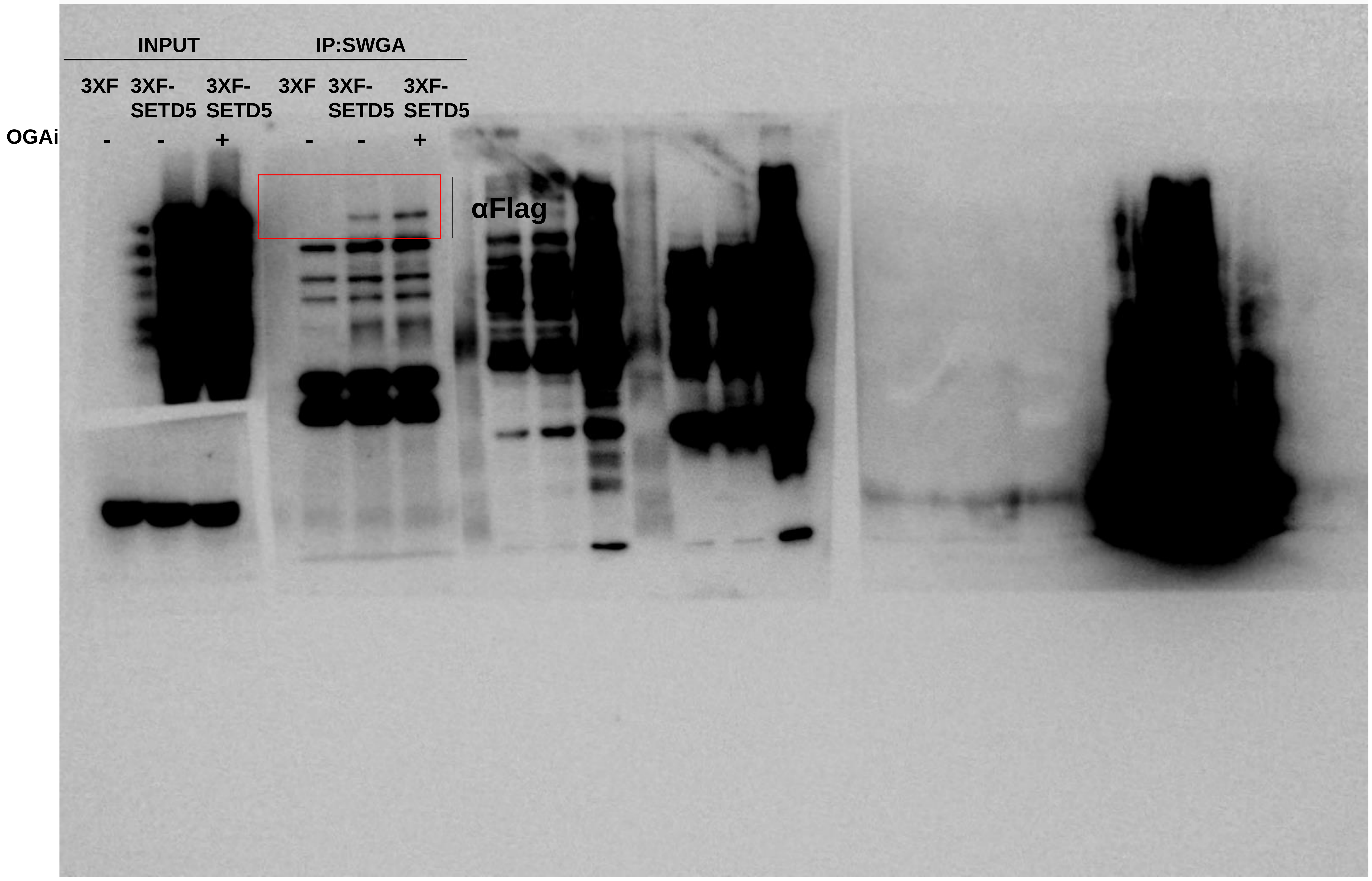
INPUT
IP:SWGA
3XF
3XF-SETD5
3XF-SETD5
3XF
3XF-SETD5
3XF-SETD5
OGAi
-
-
+
-
-
+
αFlag

Fig3B-2

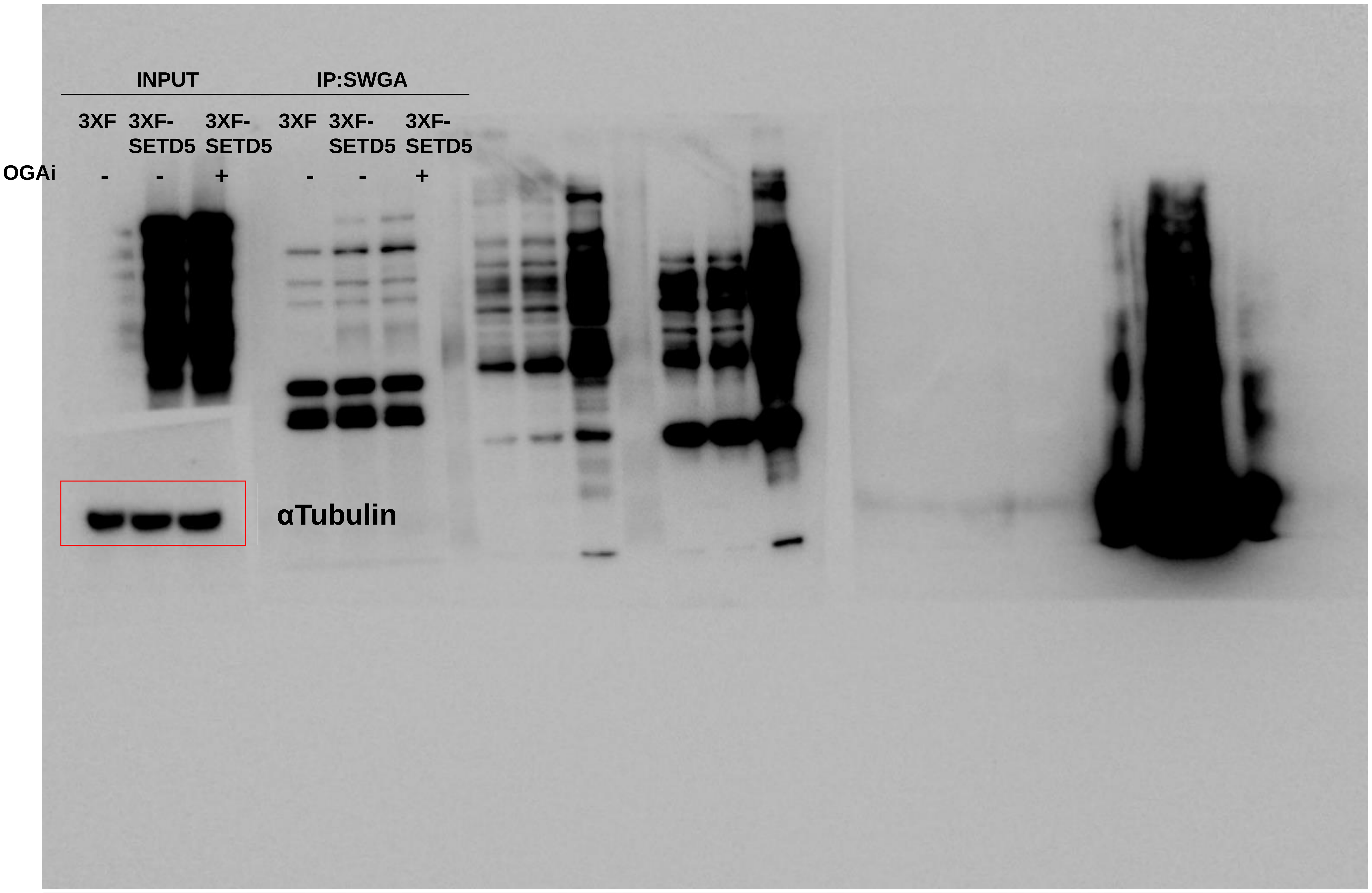
INPUT
IP:SWGA
3XF
3XF-SETD5
3XF-SETD5
3XF
3XF-SETD5
3XF-SETD5
OGAi
- - + - - +
αTubulin

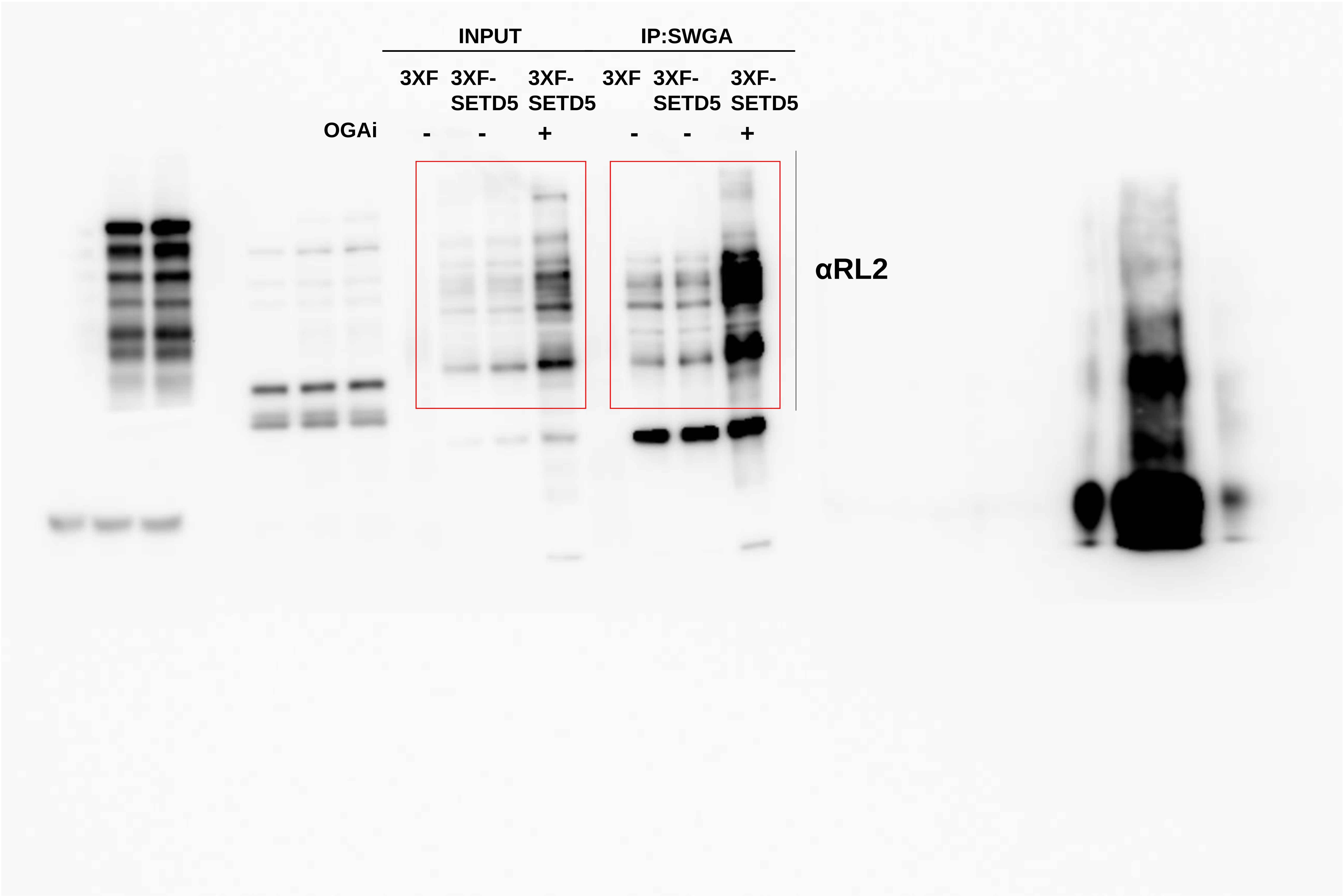
INPUT
IP:SWGA
3XF
3XF-SETD5
3XF-SETD5
3XF
3XF-SETD5
3XF-SETD5
OGAi
- - + - - +
αRL2

Fig3D-1

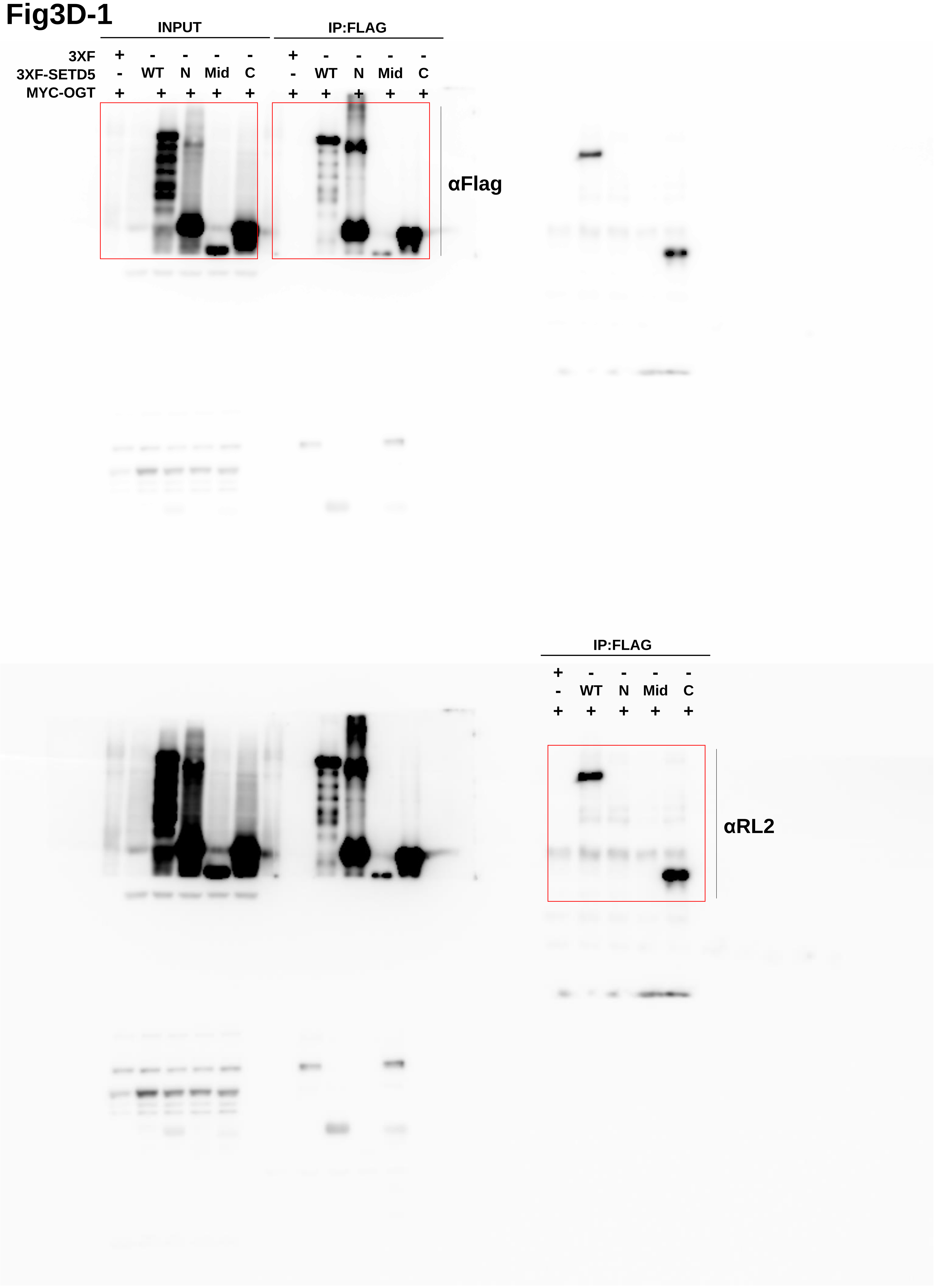

INPUT
IP:FLAG
3XF
3XF-SETD5
MYC-OGT
+ - - - - + - - - -
- WT N Mid C - WT N Mid C
+ + + + + + + + + +
αFlag
IP:FLAG
+ - - - -
- WT N Mid C
+ + + + +
αRL2

Fig3D-2

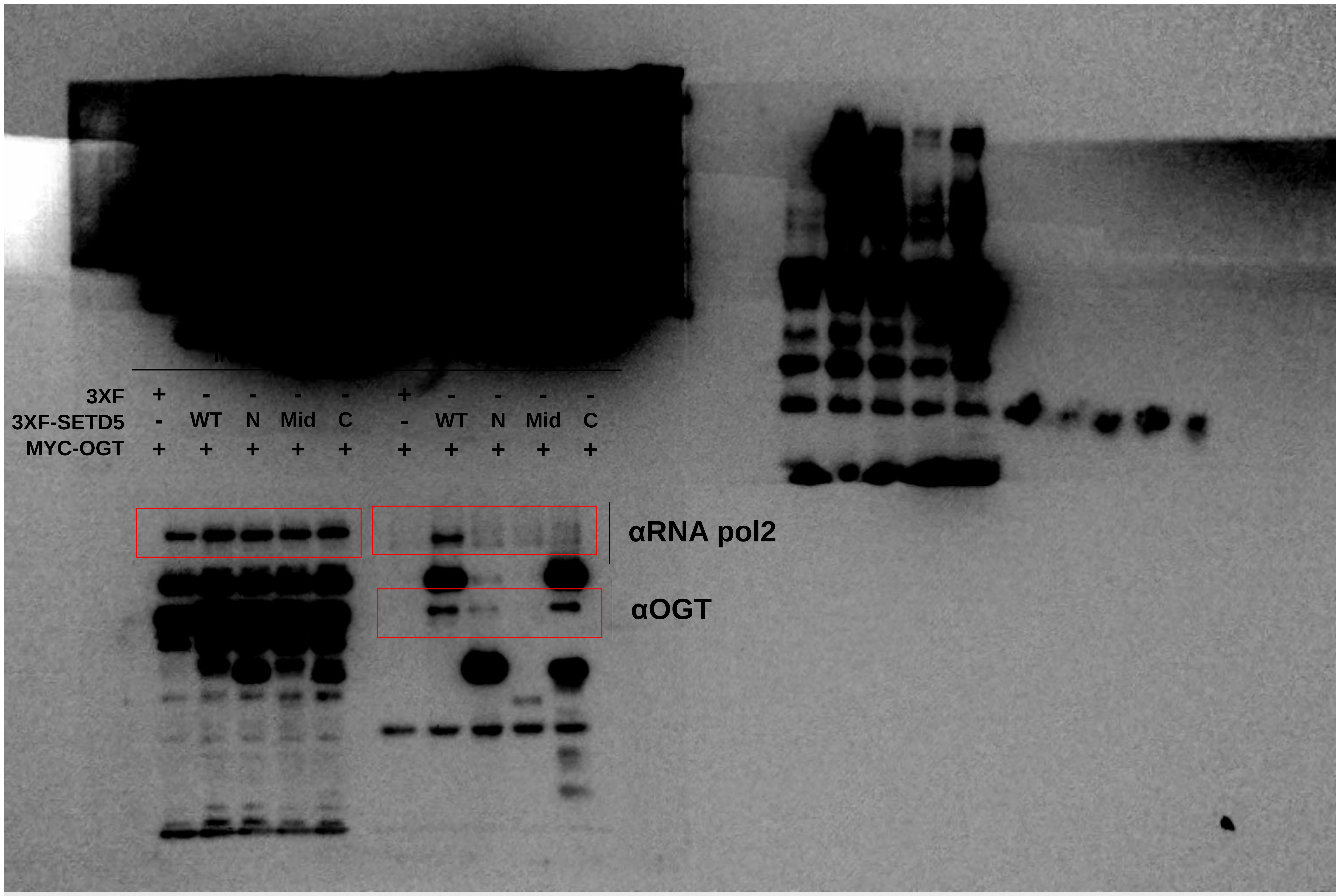

3XF + - - - - + - - - -
3XF-SETD5 - WT N Mid C - WT N Mid C
MYC-OGT + + + + + + + + + +
αRNA pol2
αOGT

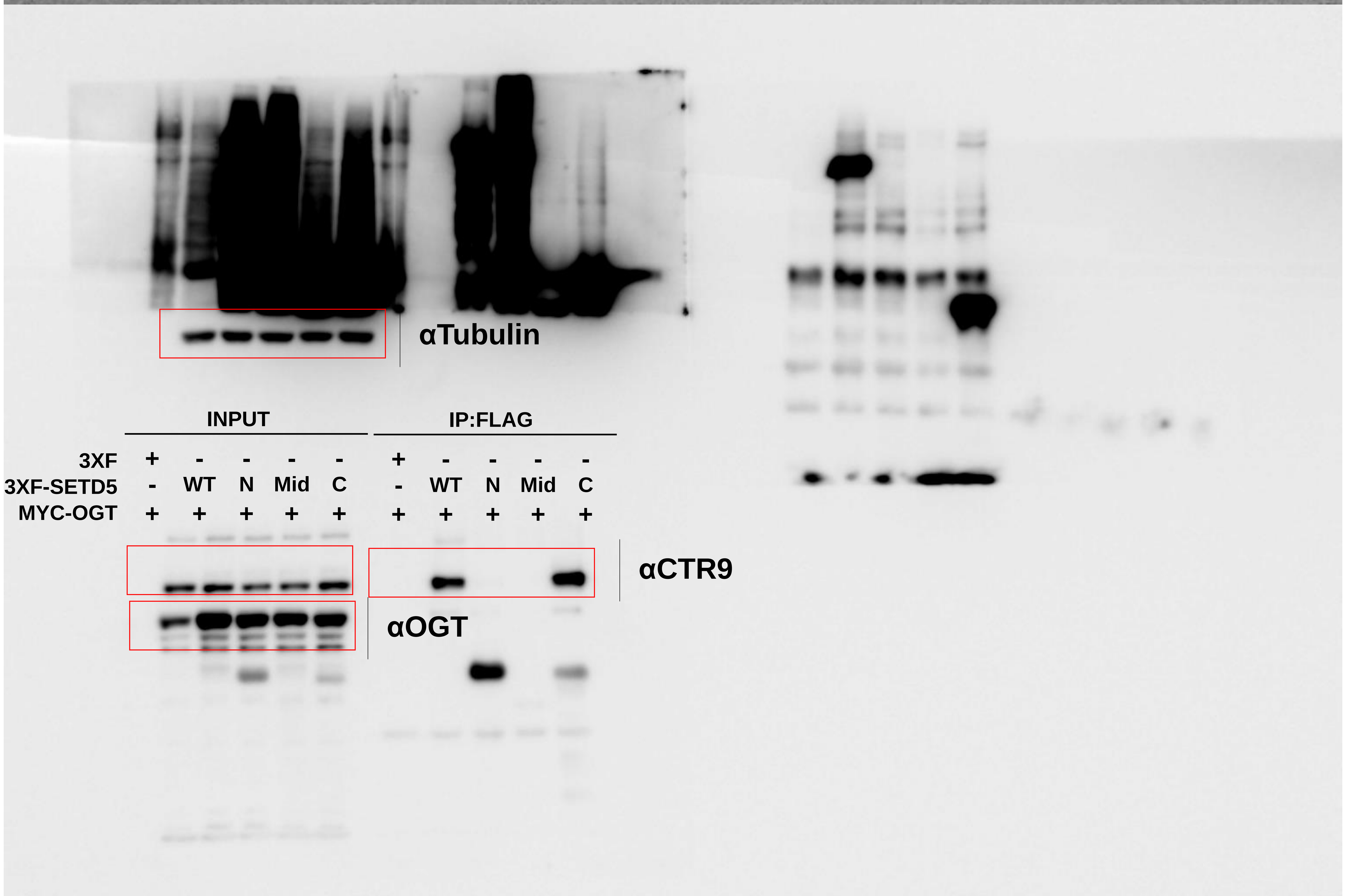

αTubulin
INPUT
IP:FLAG
3XF + - - - - + - - - -
3XF-SETD5 - WT N Mid C - WT N Mid C
MYC-OGT + + + + + + + + + +
αCTR9
αOGT

Fig4A-1

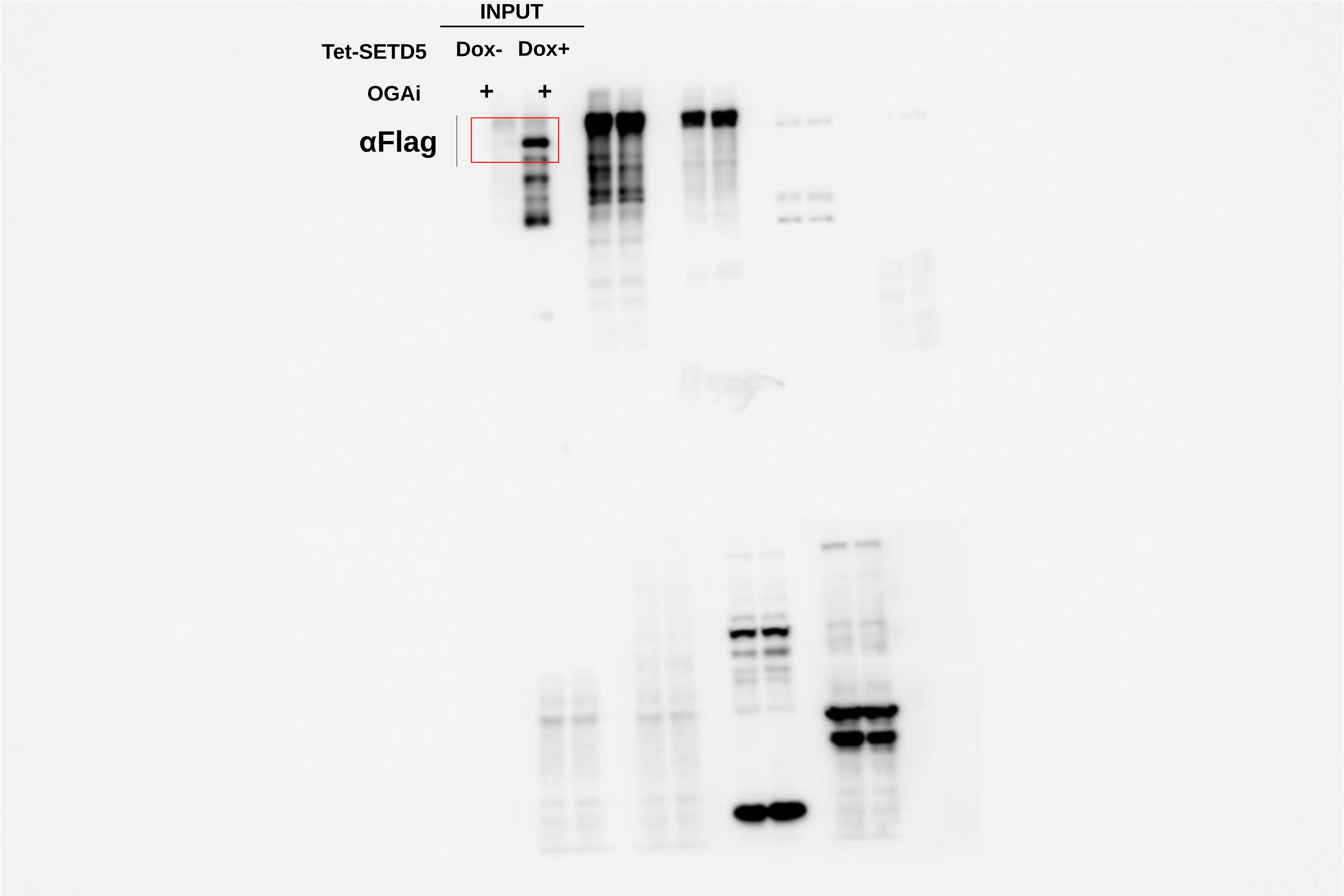
INPUT
Tet-SETD5
Dox-
Dox+
OGAi
+
+
αFlag

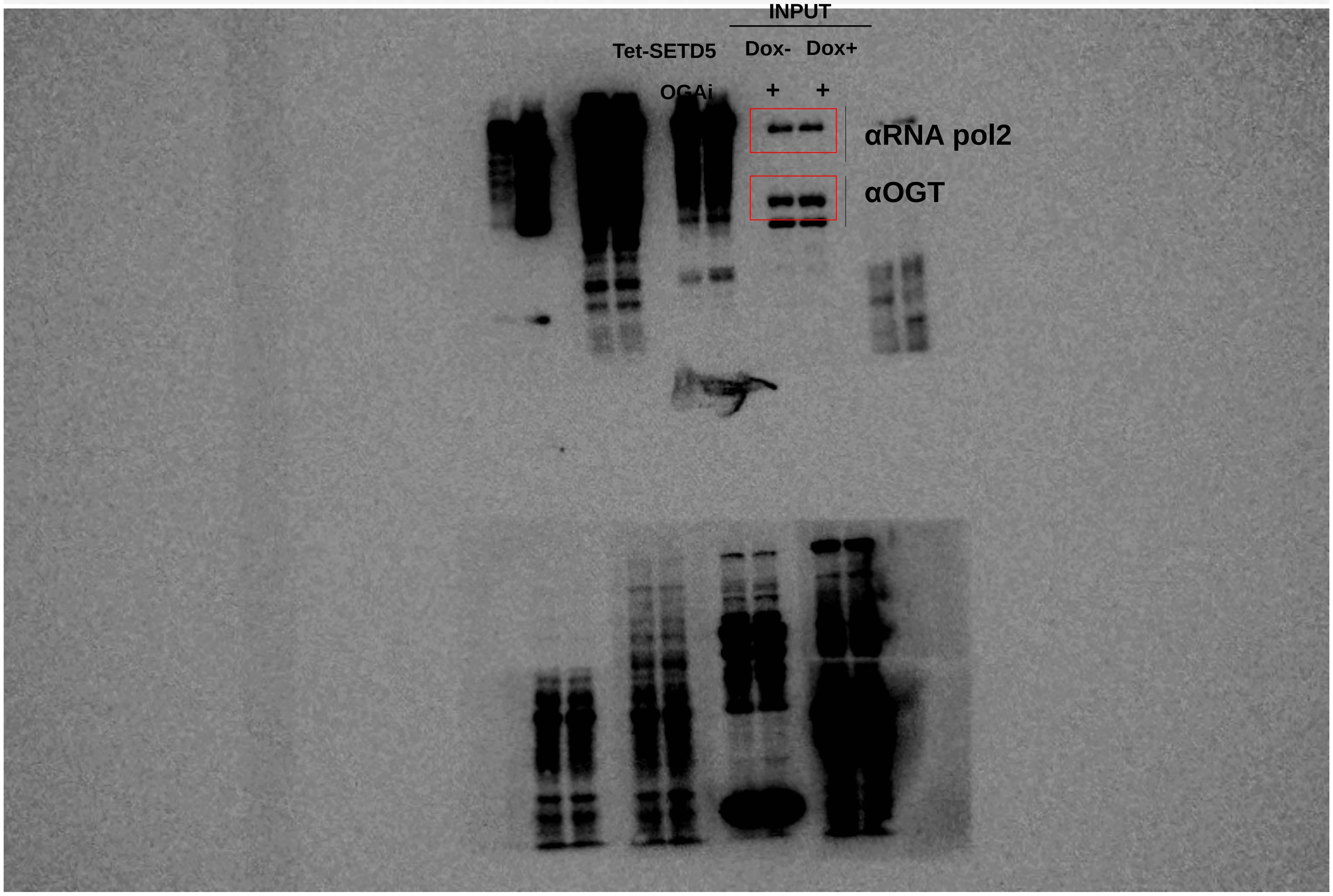
INPUT
Tet-SETD5
Dox-
Dox+
OGAi
+
+
αRNA pol2
αOGT

Fig4A-2

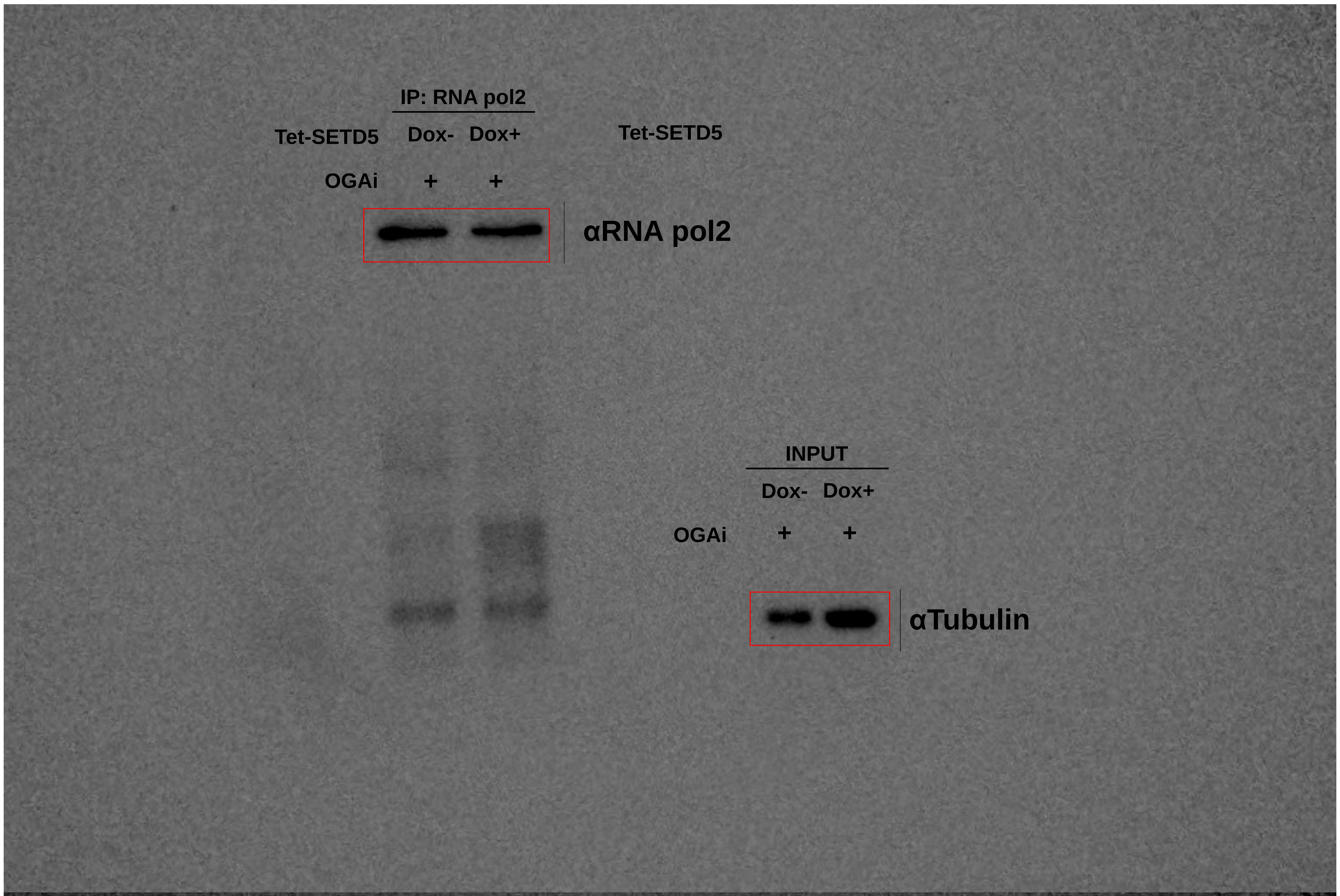
IP: RNA pol2
Tet-SETD5
Dox-
Dox+
Tet-SETD5
OGAi
+
+
αRNA pol2
INPUT
Dox-
Dox+
OGAi
+
+
αTubulin

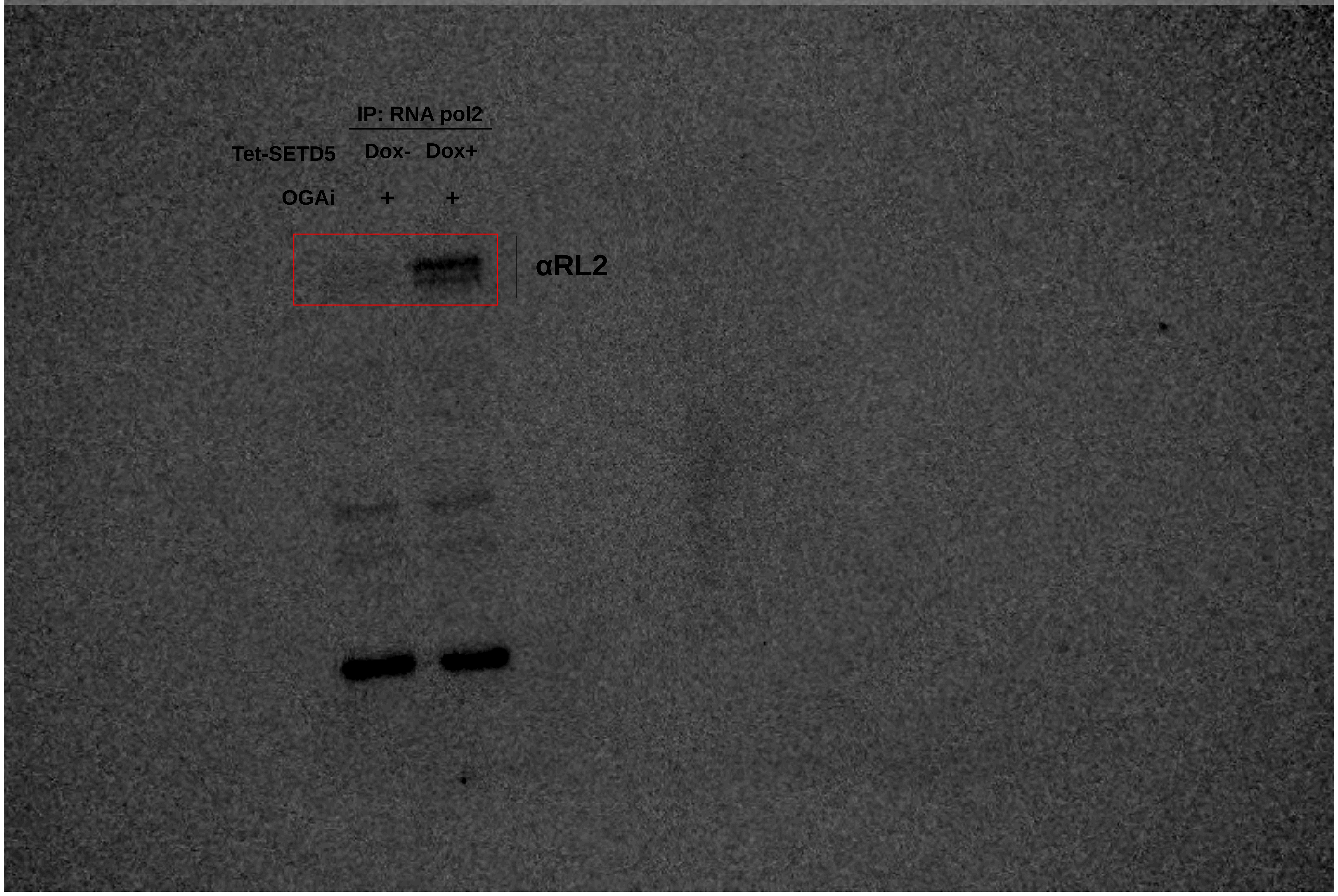
IP: RNA pol2
Tet-SETD5
Dox-
Dox+
OGAi
+
+
αRL2

Fig4B-1

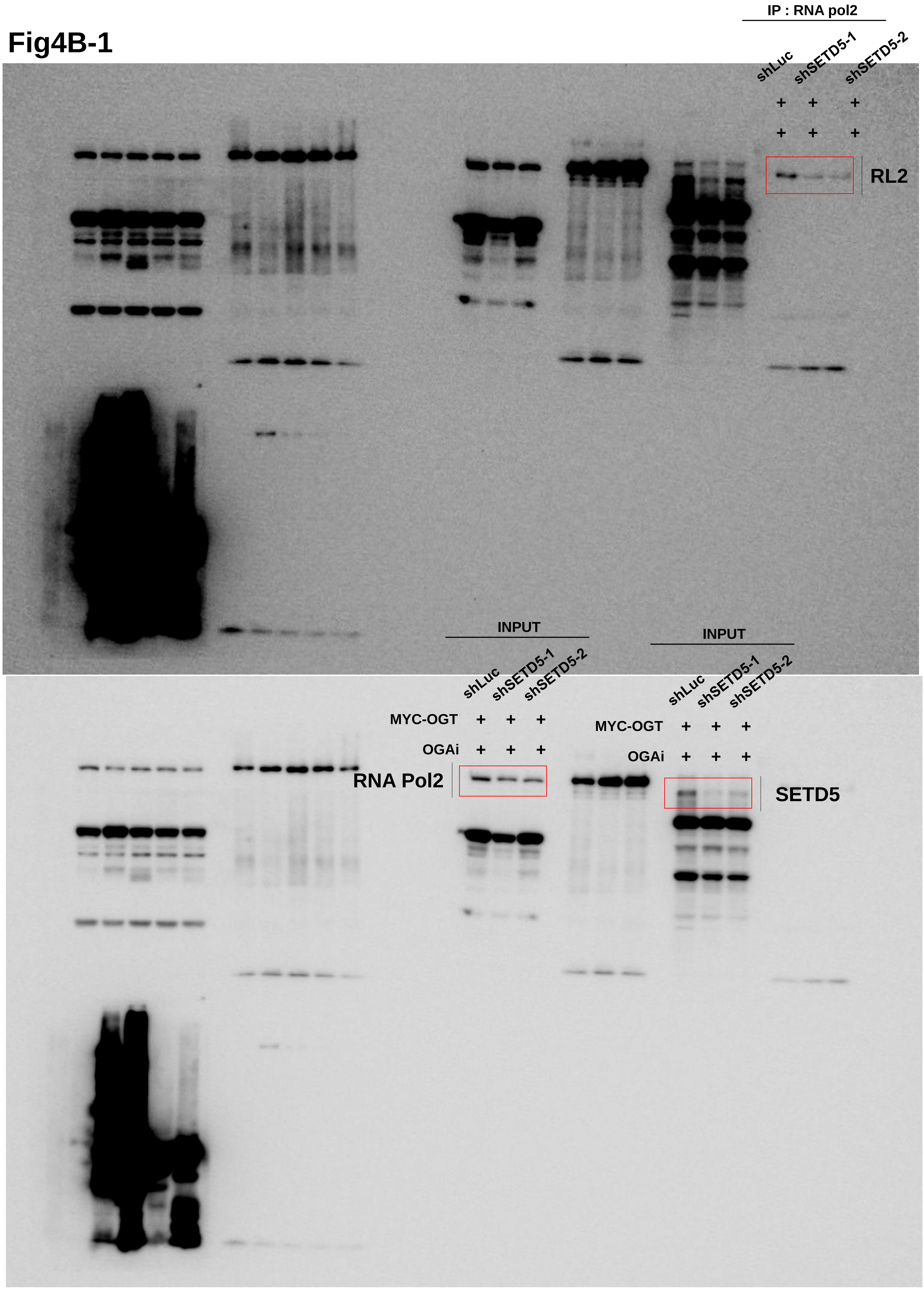

IP : RNA pol2
shLuc
shSETD5-1
shSETD5-2
+ + +
+ + +
RL2
INPUT
shLuc
shSETD5-1
shSETD5-2
MYC-OGT + + +
OGAi + + +
RNA Pol2
INPUT
shLuc
shSETD5-1
shSETD5-2
MYC-OGT + + +
OGAi + + +
SETD5

Fig4B-2

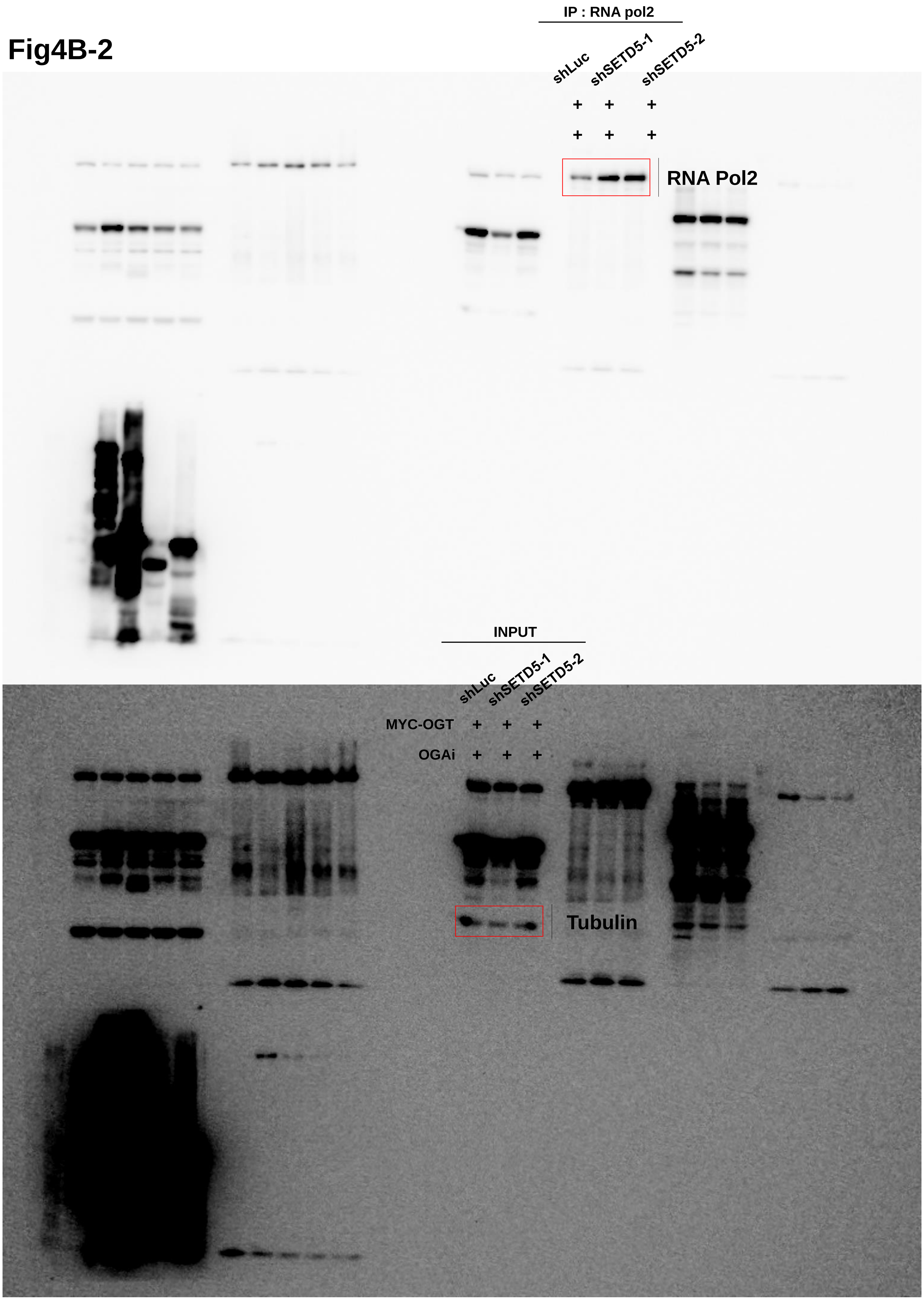
IP : RNA pol2
shLuc
shSETD5-1
shSETD5-2
+ + +
+ + +
RNA Pol2
INPUT
shLuc
shSETD5-1
shSETD5-2
MYC-OGT + + +
OGAi + + +
Tubulin

Fig4C-1

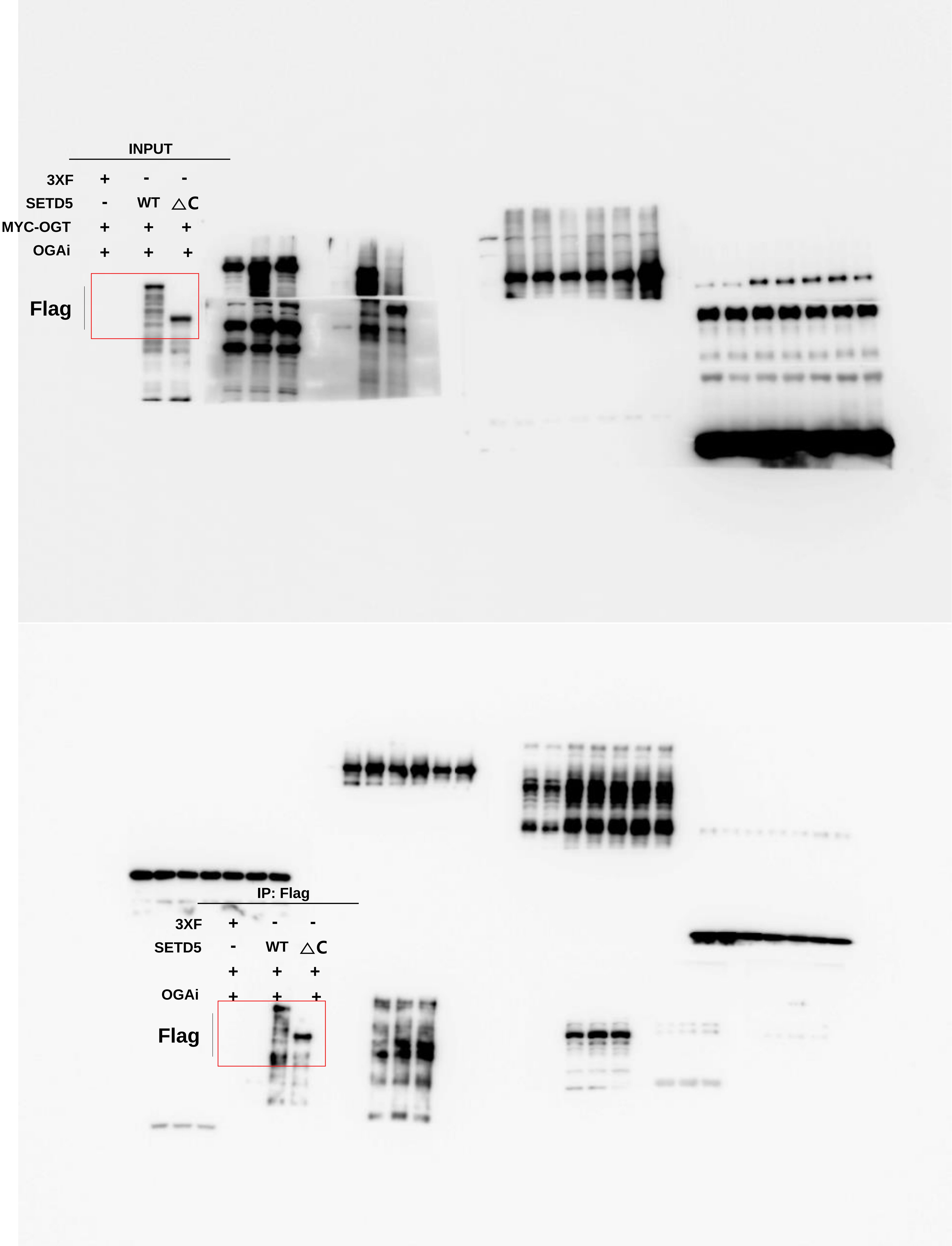
INPUT
3XF + - -
SETD5 - WT △C
MYC-OGT + + +
OGAi + + +
Flag
IP: Flag
3XF + - -
SETD5 - WT △C
+ + +
OGAi + + +
Flag

Fig4C-2

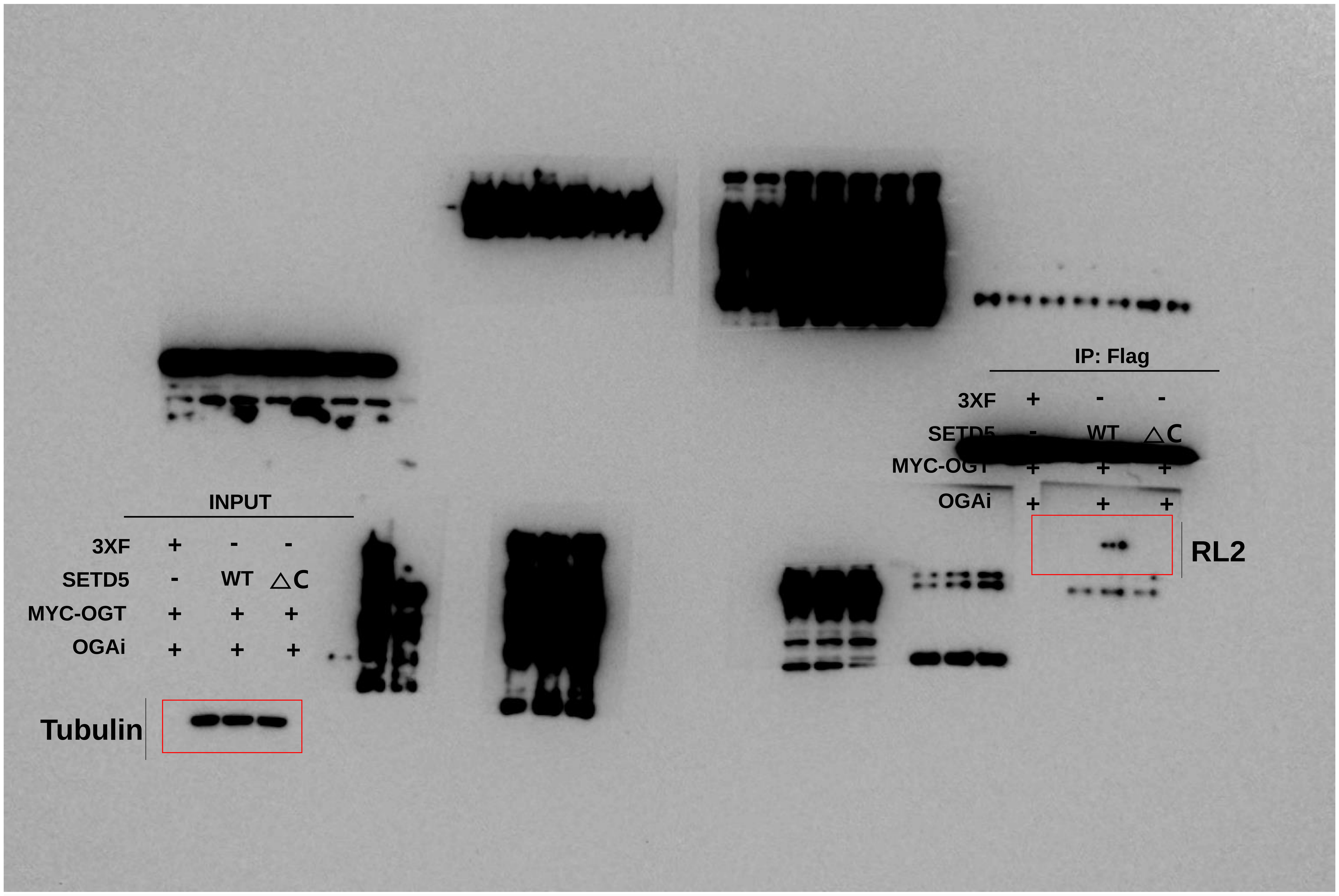
IP: Flag
3XF + - -
SETD5 - WT △C
MYC-OGT + + +
OGAi + + +
RL2
INPUT
3XF + - -
SETD5 - WT △C
MYC-OGT + + +
OGAi + + +
Tubulin

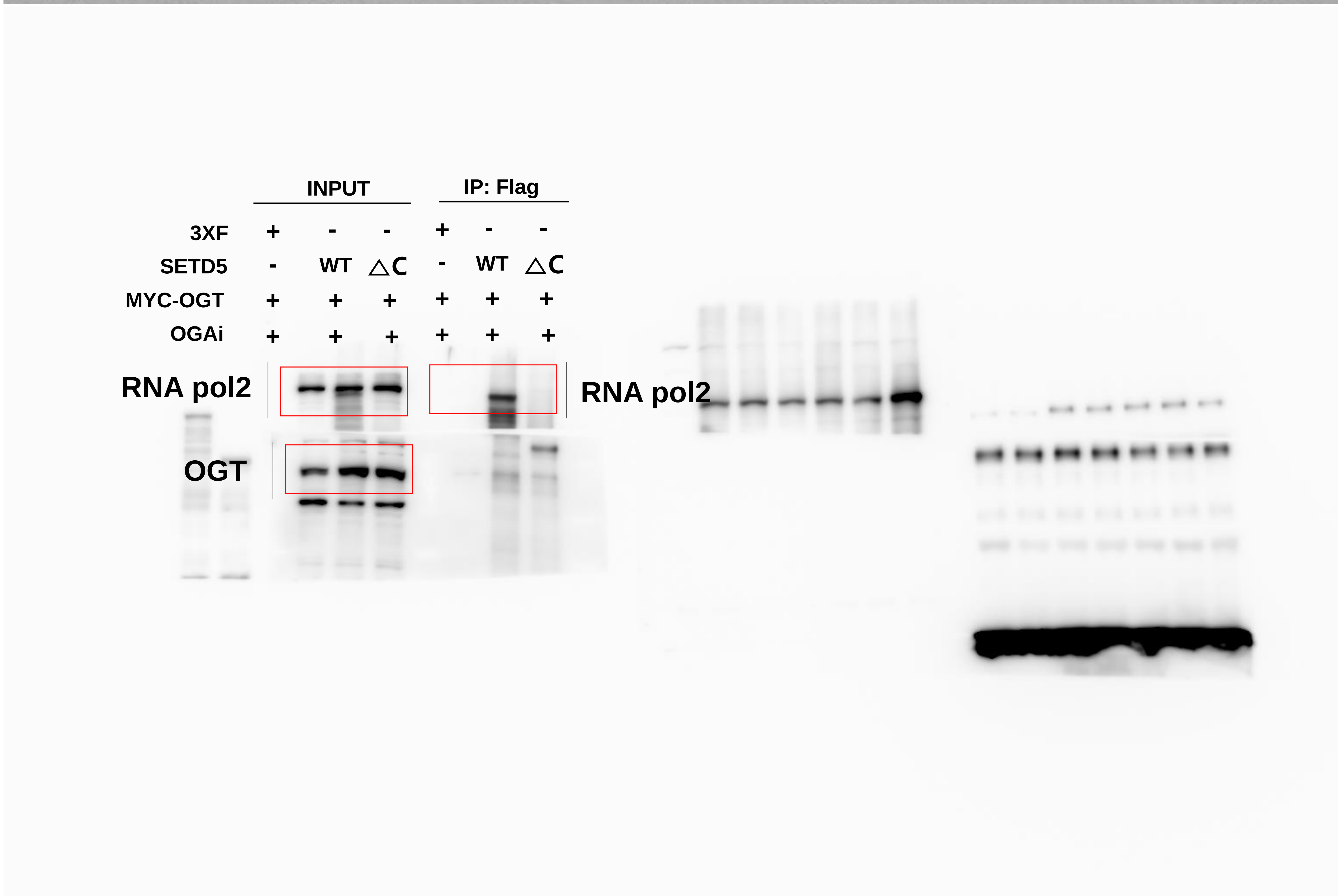
INPUT
IP: Flag
3XF + - - + - -
SETD5 - WT △C - WT △C
MYC-OGT + + + + + +
OGAi + + + + + +
RNA pol2
RNA pol2
OGT

Fig4D-1

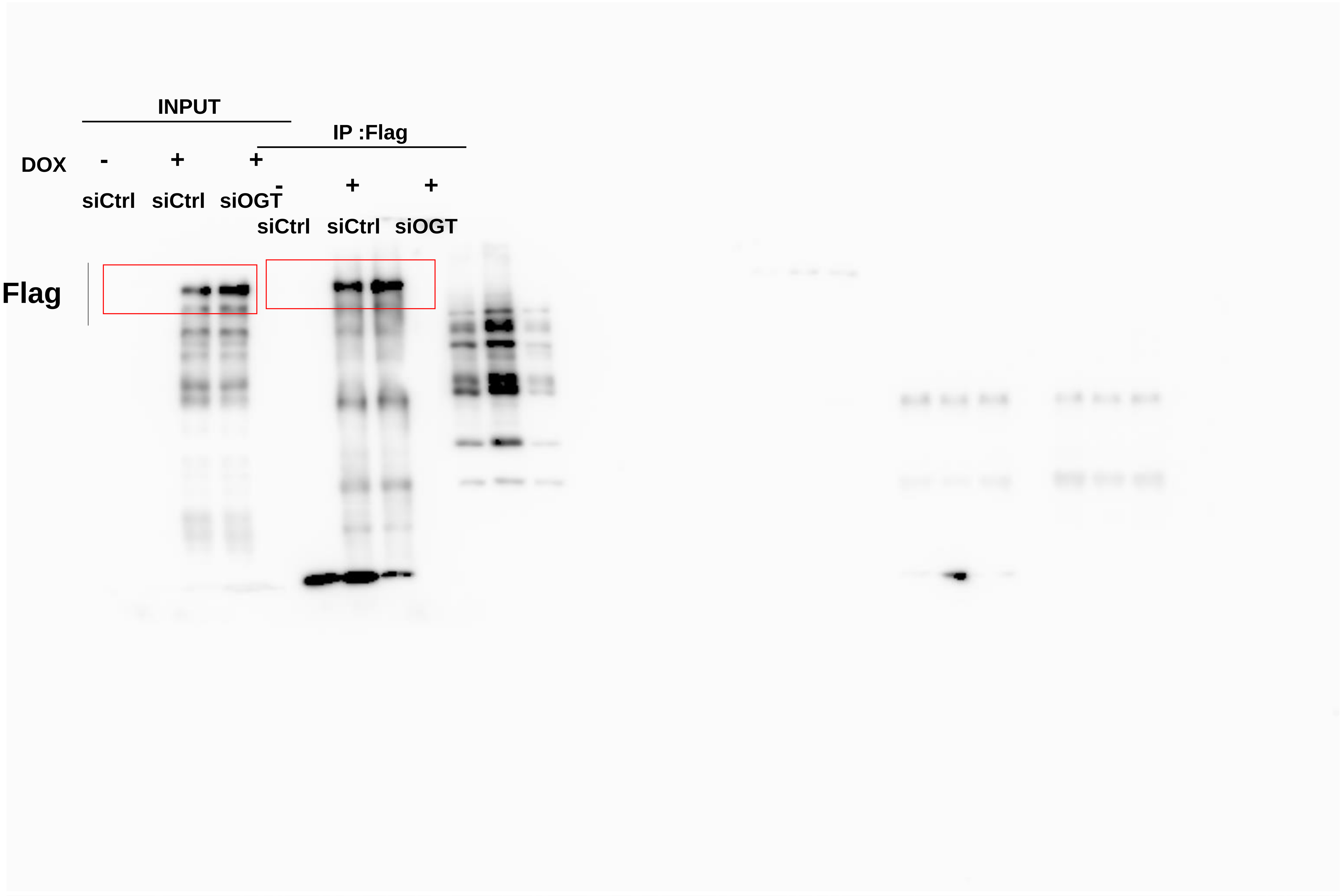
INPUT
IP :Flag
DOX
- + +
siCtrl siCtrl siOGT
- + +
siCtrl siCtrl siOGT
Flag

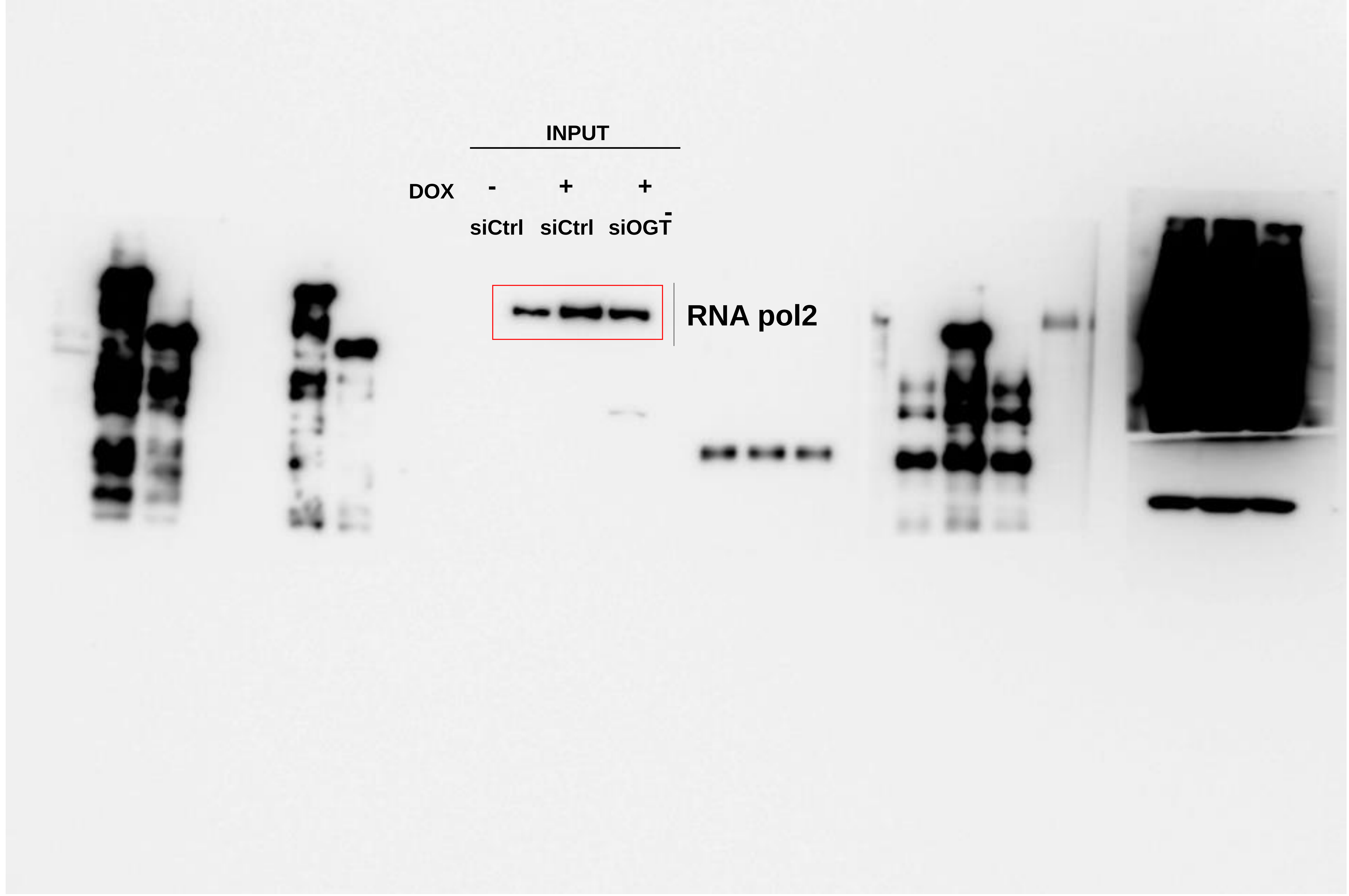
INPUT
DOX
- + +
siCtrl siCtrl siOGT
RNA pol2

Fig4D-2

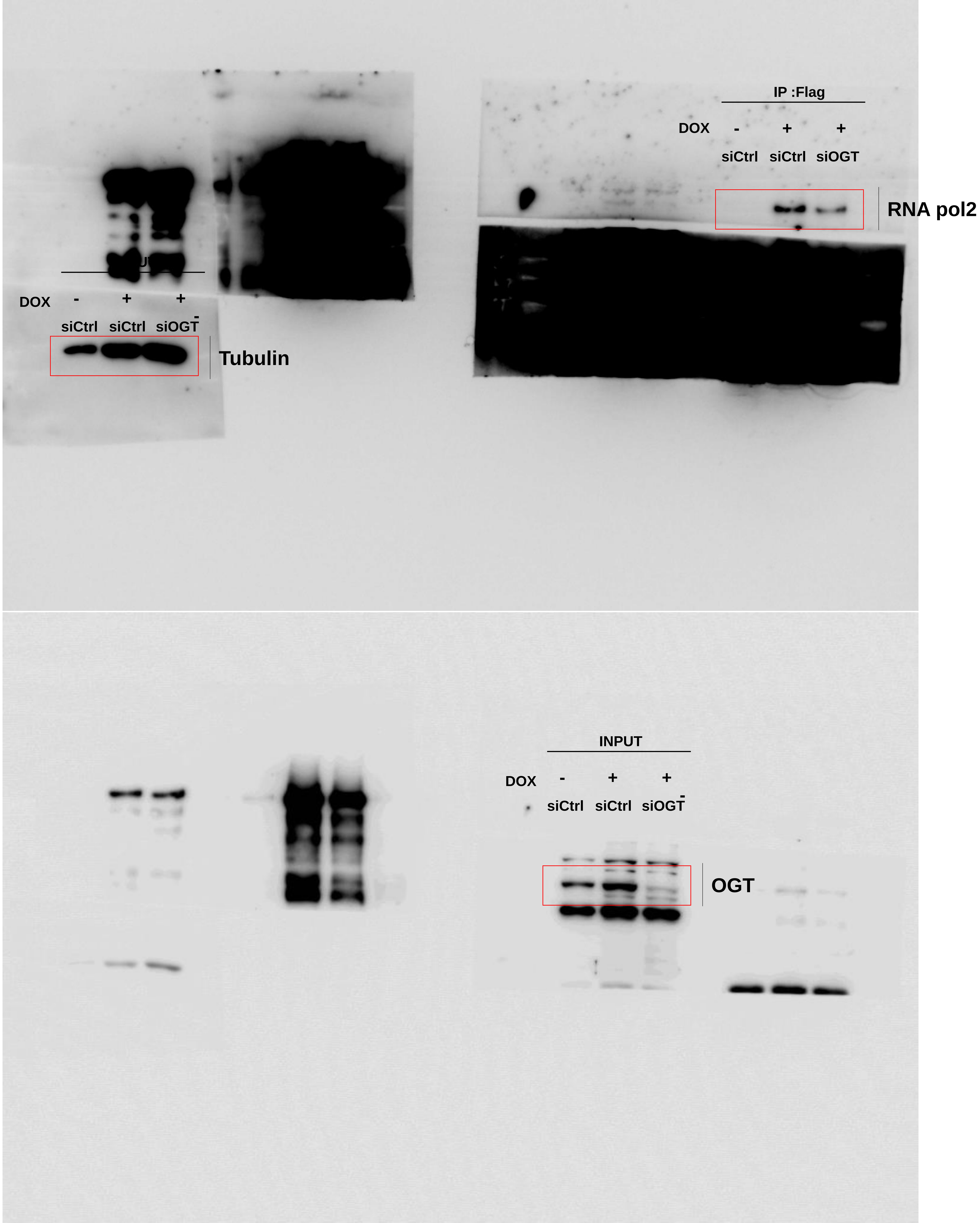
IP :Flag
DOX - + +
siCtrl siCtrl siOGT
RNA pol2
DOX - + +
siCtrl siCtrl siOGT
Tubulin
INPUT
DOX - + +
siCtrl siCtrl siOGT
OGT

Fig4D-3

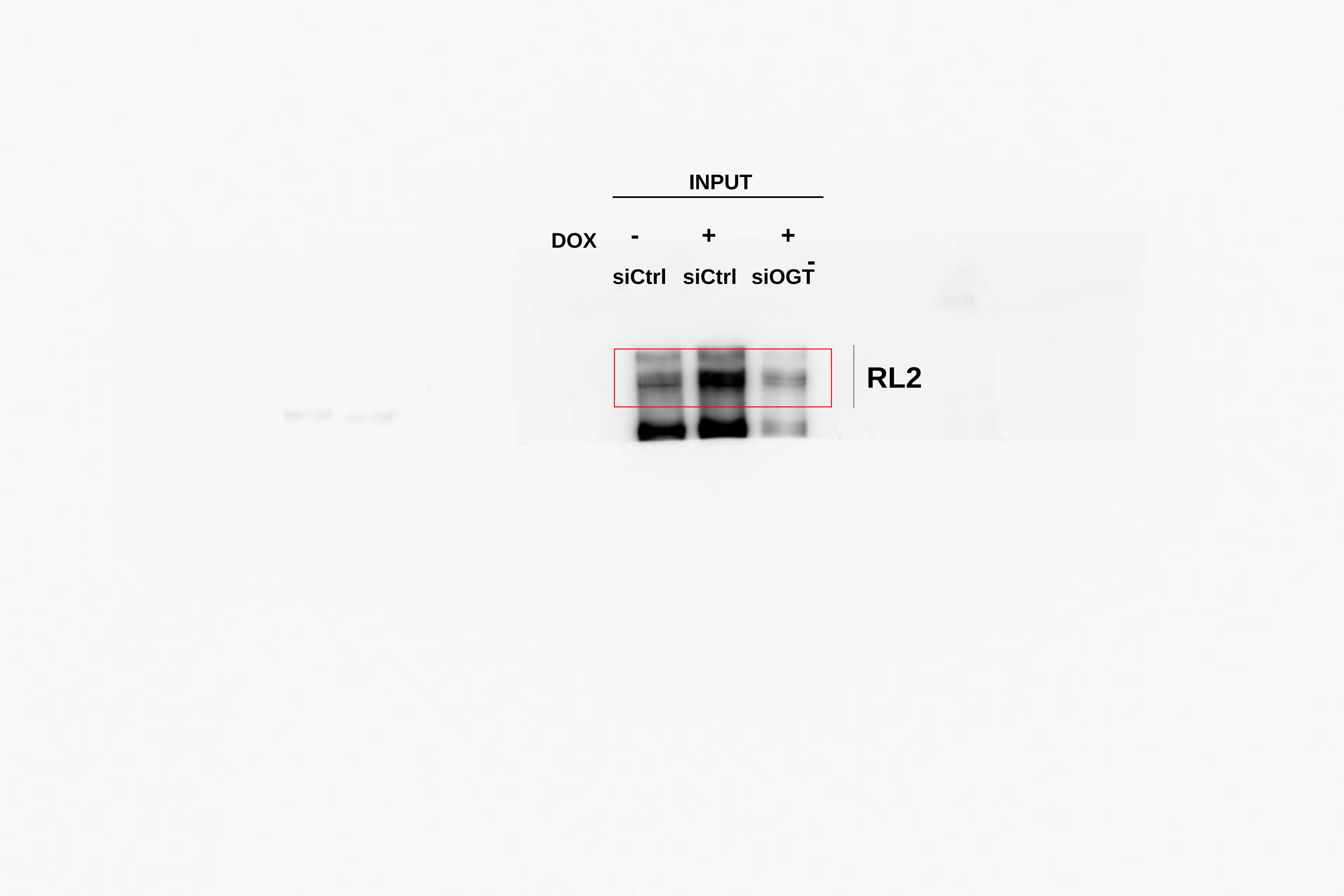
INPUT
DOX - + +
siCtrl siCtrl siOGT
RL2

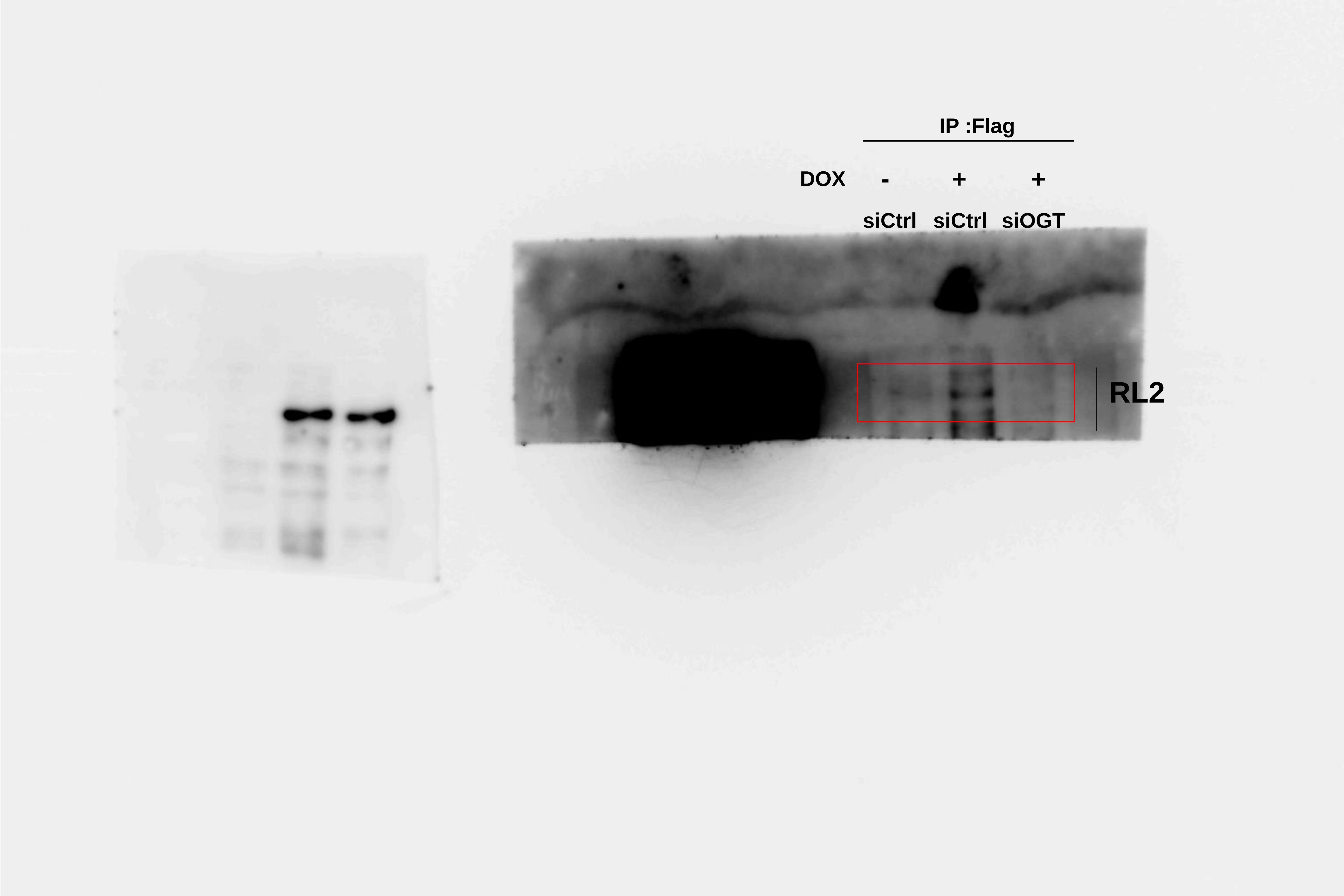
IP :Flag
DOX - + +
siCtrl siCtrl siOGT
RL2

Fig5E-1

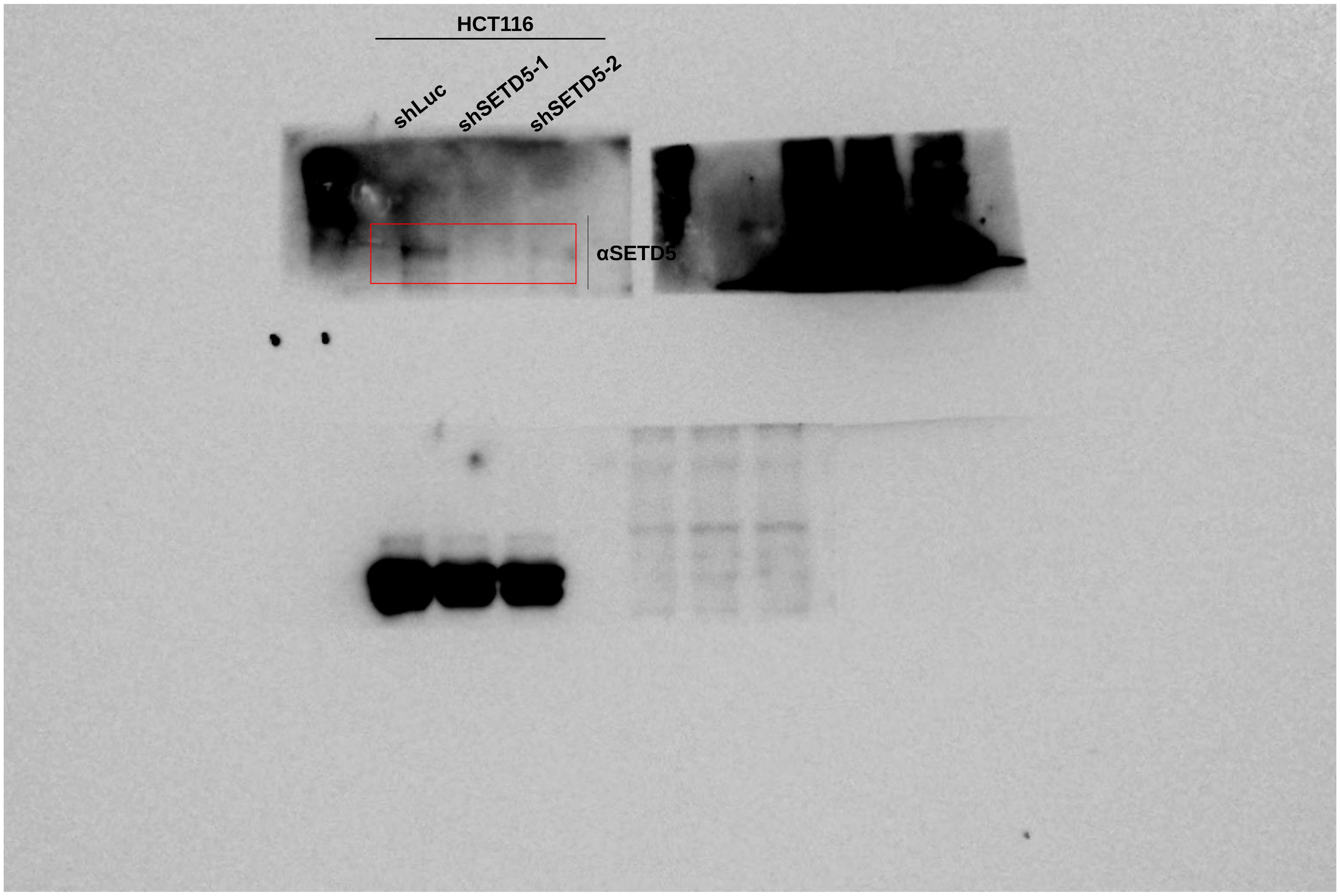
HCT116
shLuc
shSETD5-1
shSETD5-2
αSETD5

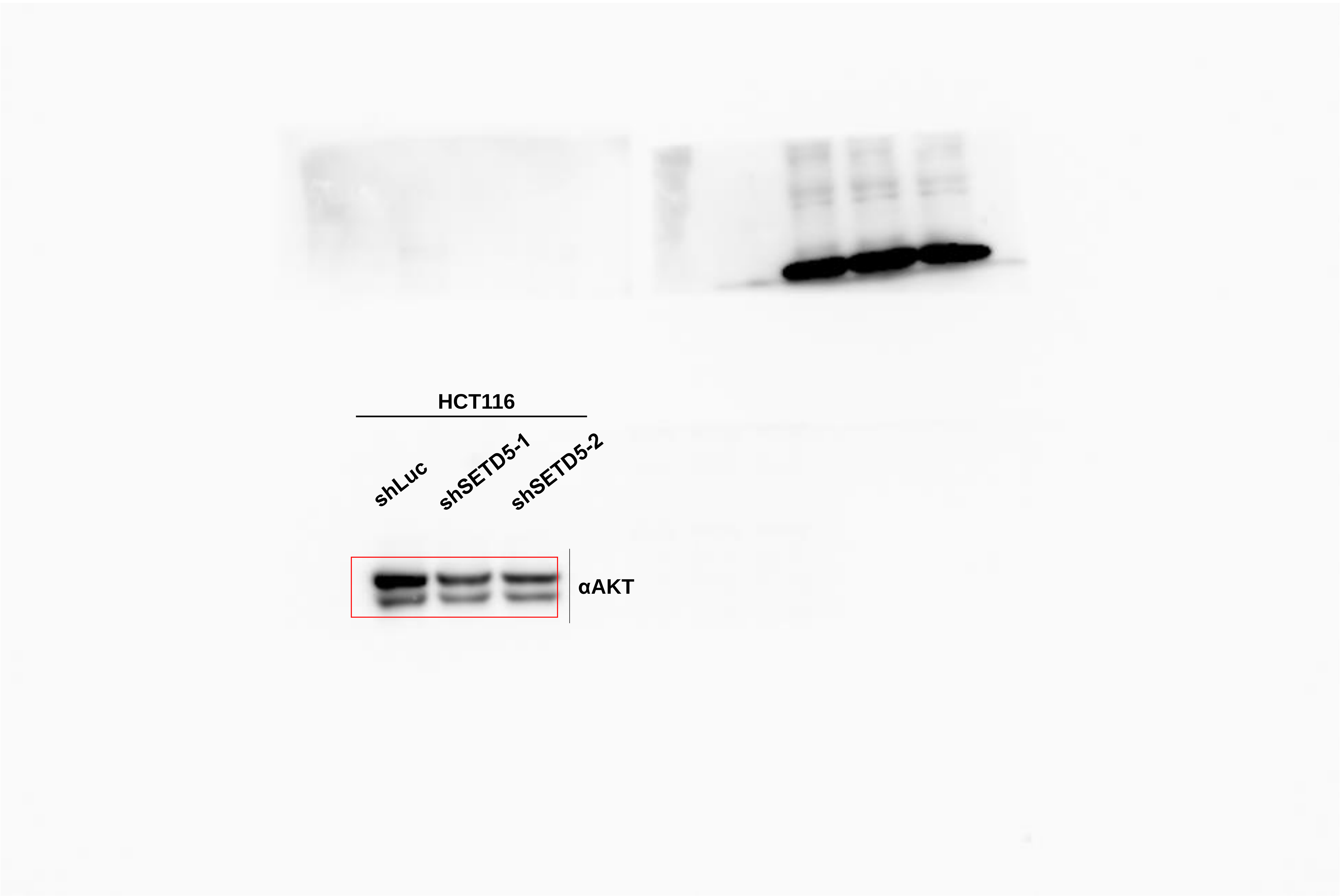
HCT116
shLuc
shSETD5-1
shSETD5-2
αAKT

Fig5E-2

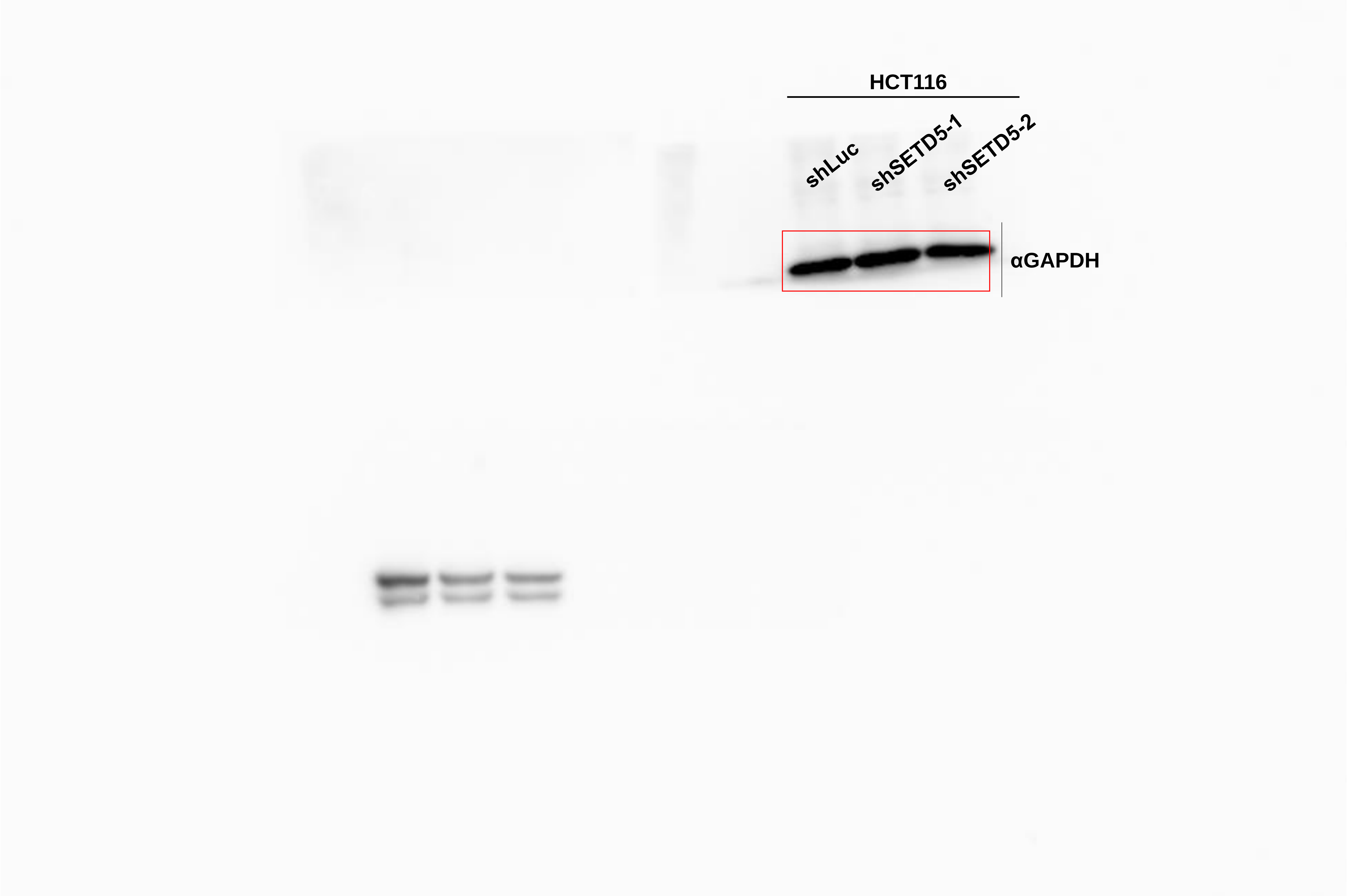

Fig6B

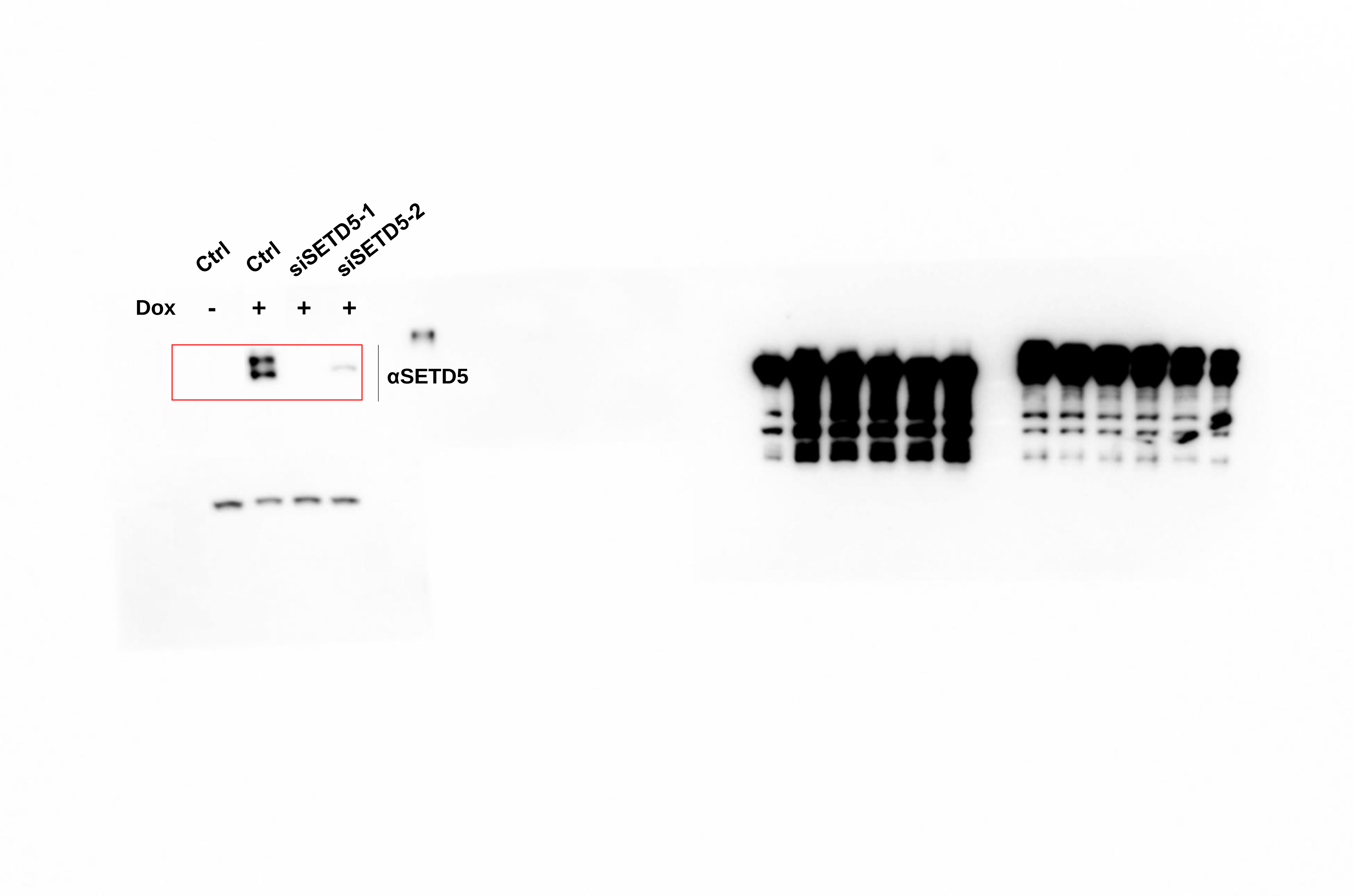
Ctrl
Ctrl
siSETD5-1
siSETD5-2
Dox - + + +
αSETD5

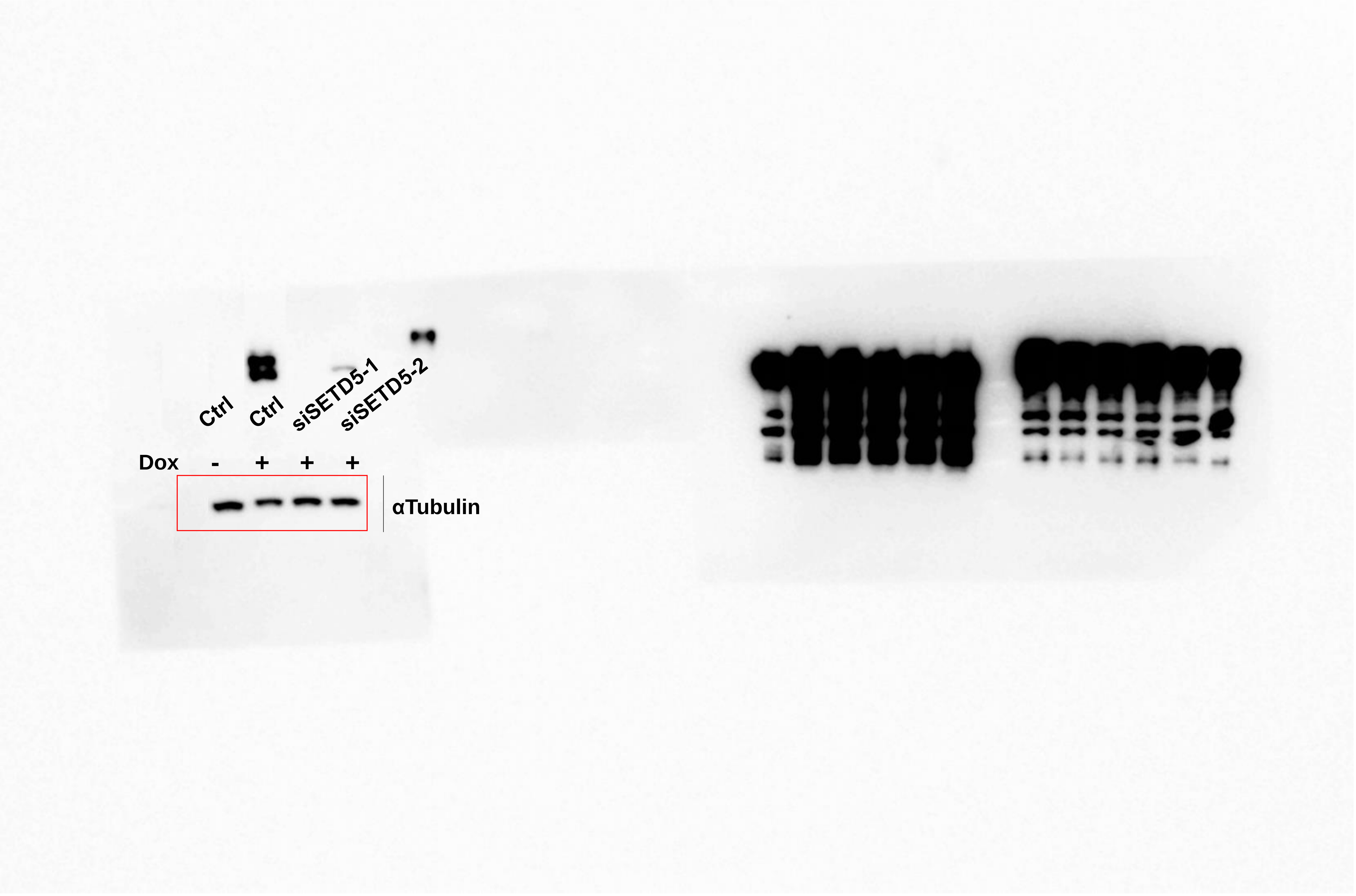
Ctrl
Ctrl
siSETD5-1
siSETD5-2
Dox - + + +
αTubulin

Fig6F

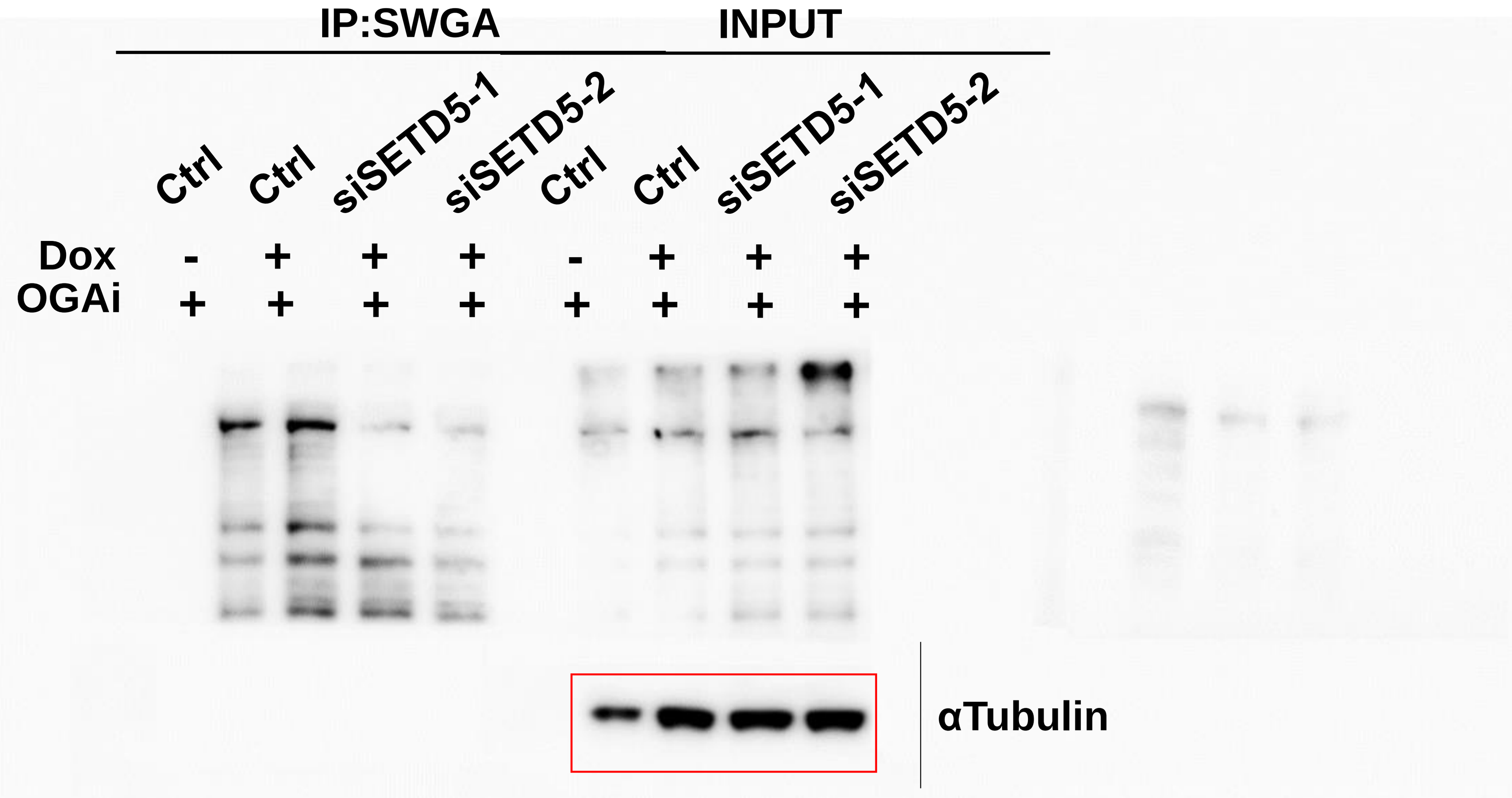
IP:SWGA
INPUT
Ctrl
Ctrl
siSETD5-1
siSETD5-2
Ctrl
Ctrl
siSETD5-1
siSETD5-2
Dox - + + + - + + +
OGAi + + + + + + + +
αTubulin

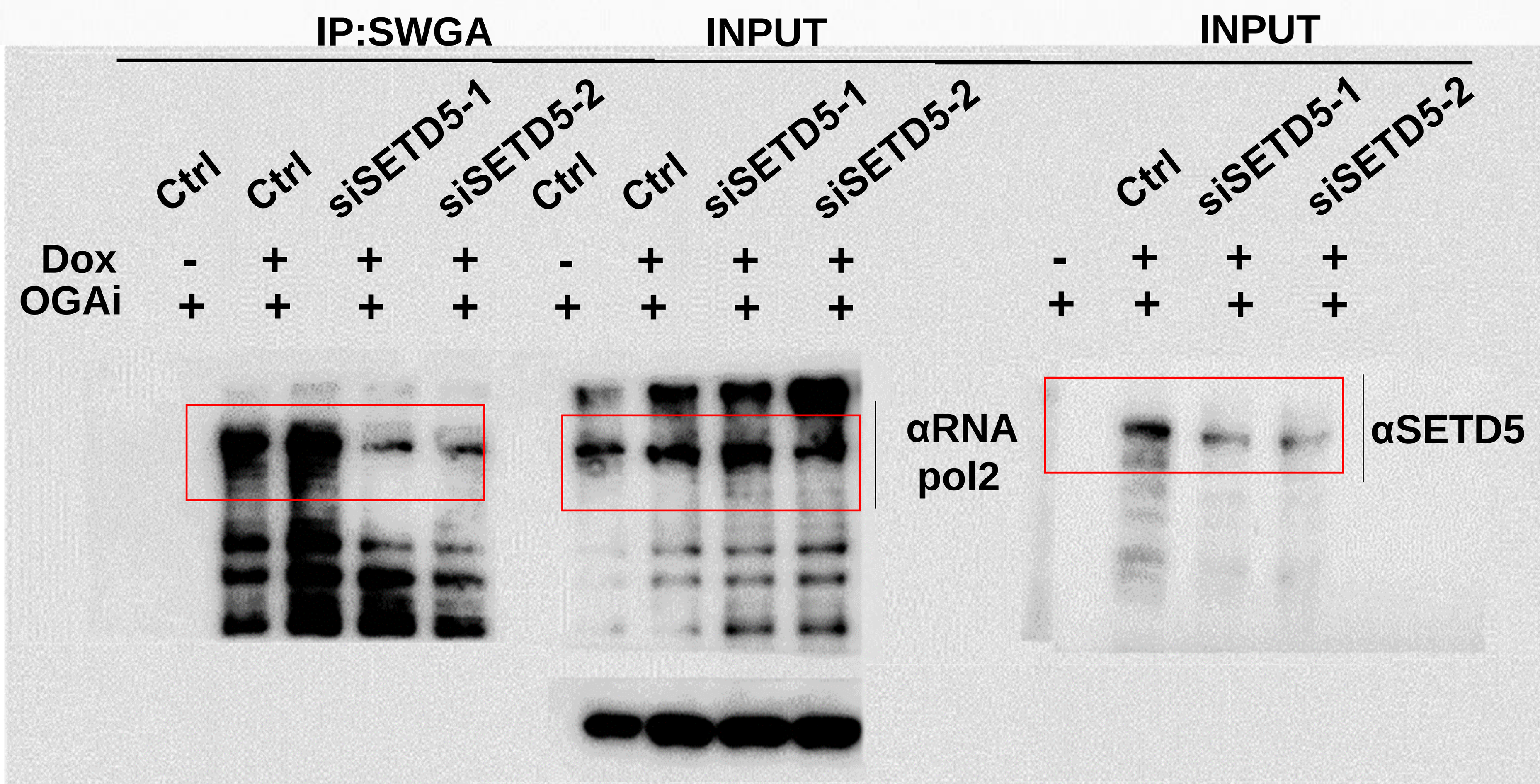
IP:SWGA
INPUT
INPUT
Ctrl
Ctrl
siSETD5-1
siSETD5-2
Ctrl
Ctrl
siSETD5-1
siSETD5-2
Ctrl
siSETD5-1
siSETD5-2
Dox - + + + - + + + - + + +
OGAi + + + + + + + + + + + +
αRNA
pol2
αSETD5

Fig6F

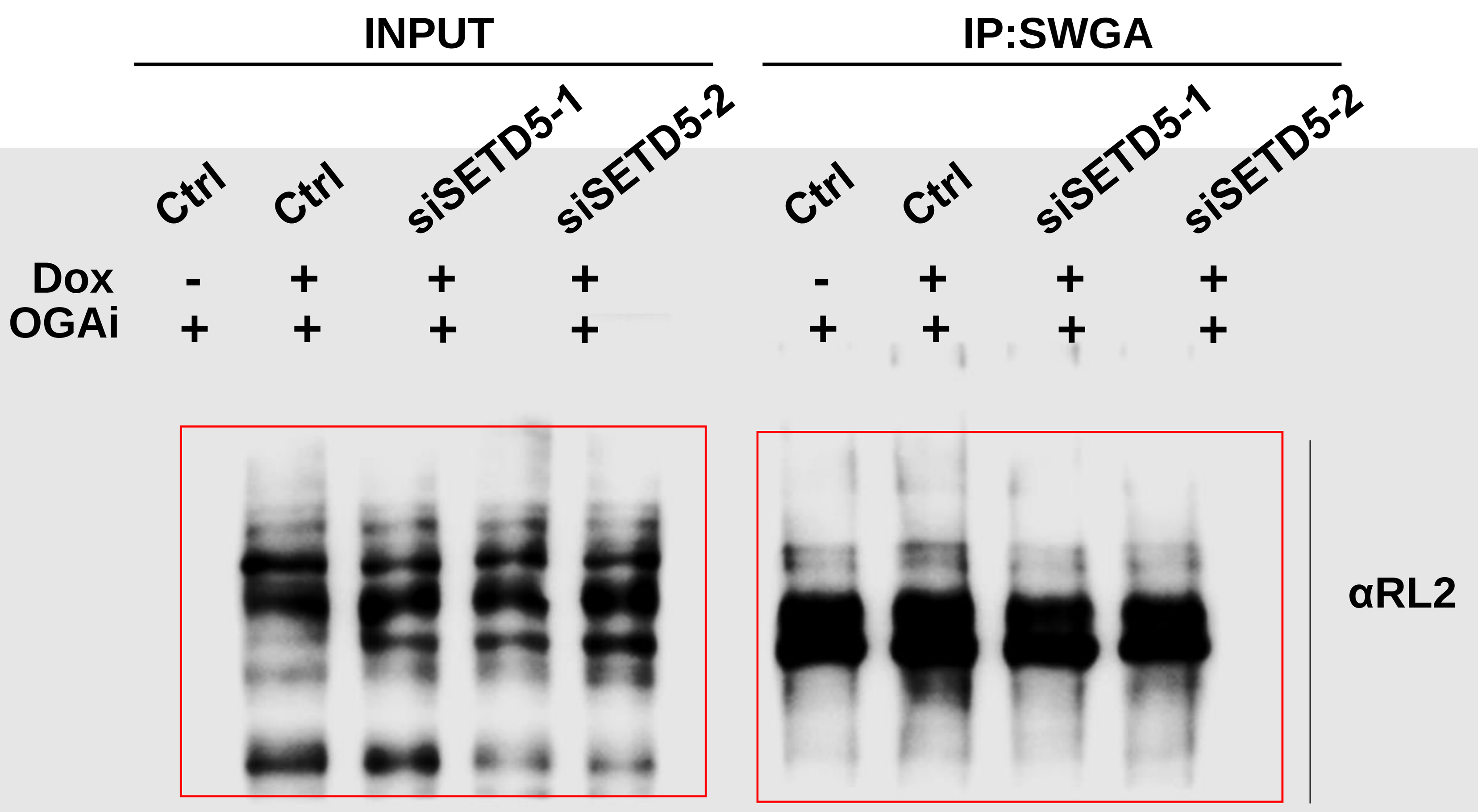
INPUT
IP:SWGA
Ctrl
Ctrl
siSETD5-1
siSETD5-2
Ctrl
Ctrl
siSETD5-1
siSETD5-2
Dox - + + + - + + +
OGAi + + + + + + + +
αRL2

SFig4A

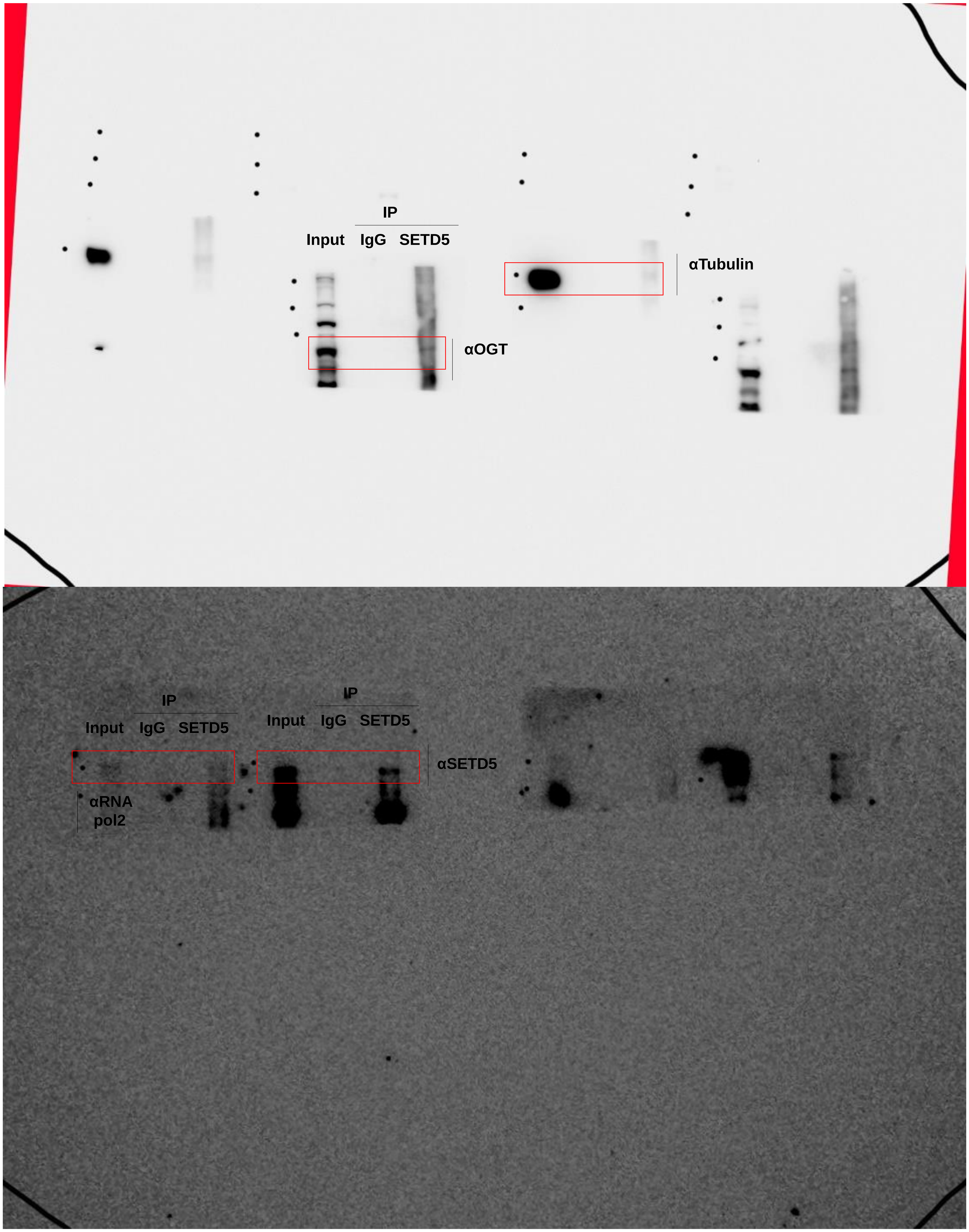
IP
Input IgG SETD5
αTubulin
αOGT
IP
Input IgG SETD5
IP
Input IgG SETD5
αSETD5
αRNA pol2

SFig4A

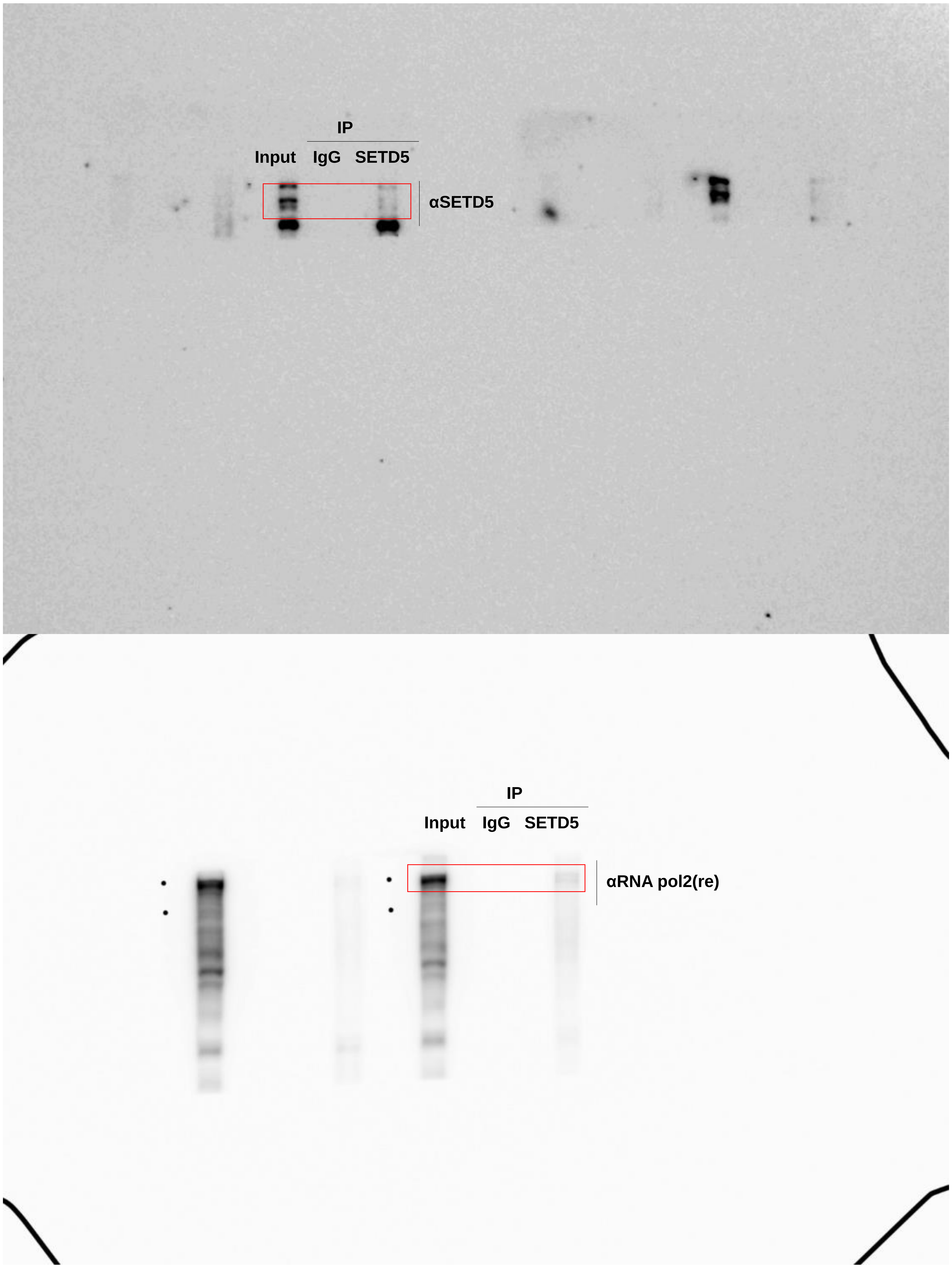

SFig4B

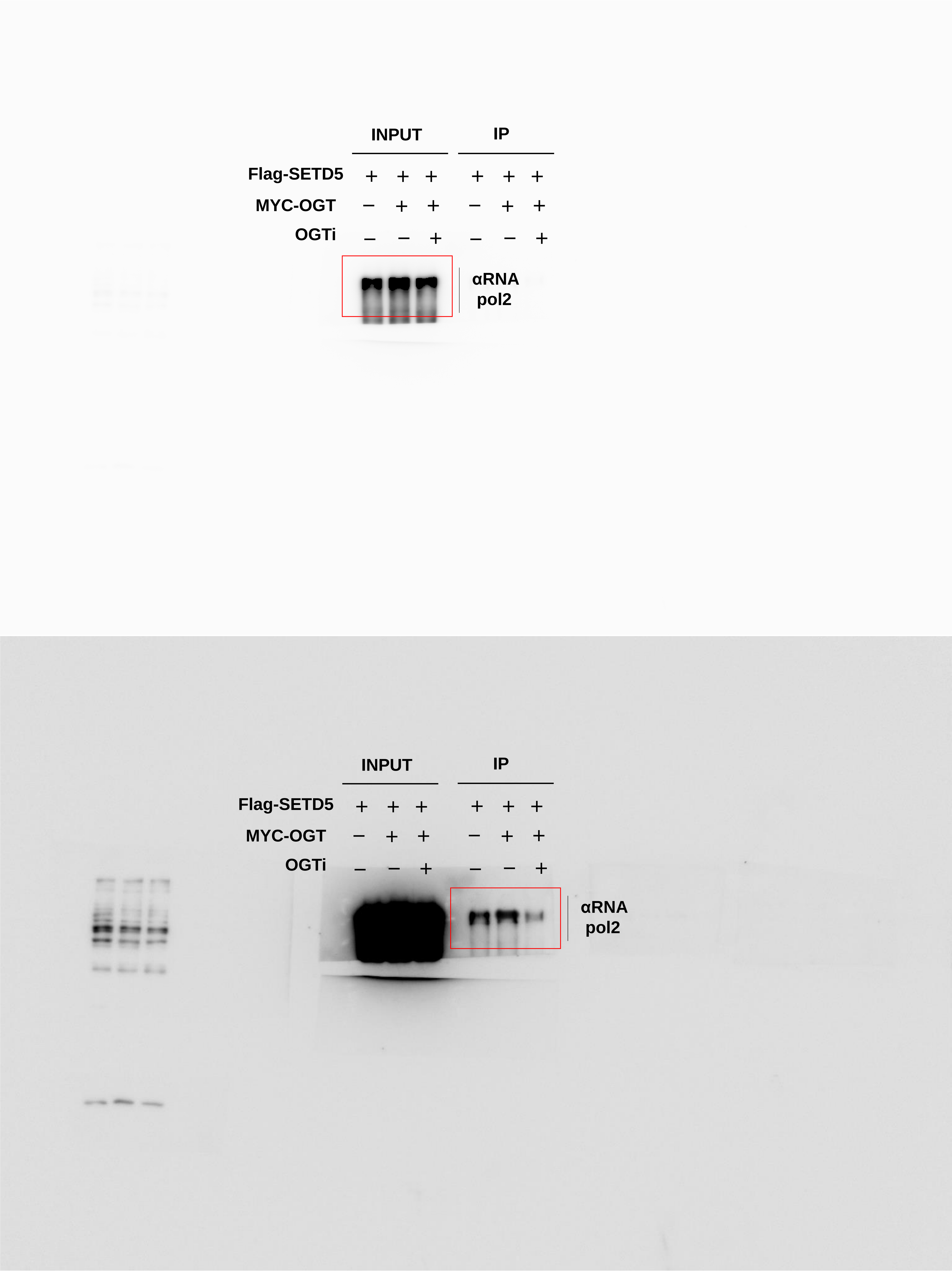
INPUT
IP
Flag-SETD5 + + + + + +
MYC-OGT − + + − + +
OGTi − − + − − +
αRNA pol2
INPUT
IP
Flag-SETD5 + + + + + +
MYC-OGT − + + − + +
OGTi − − + − − +
αRNA pol2

SFig4B

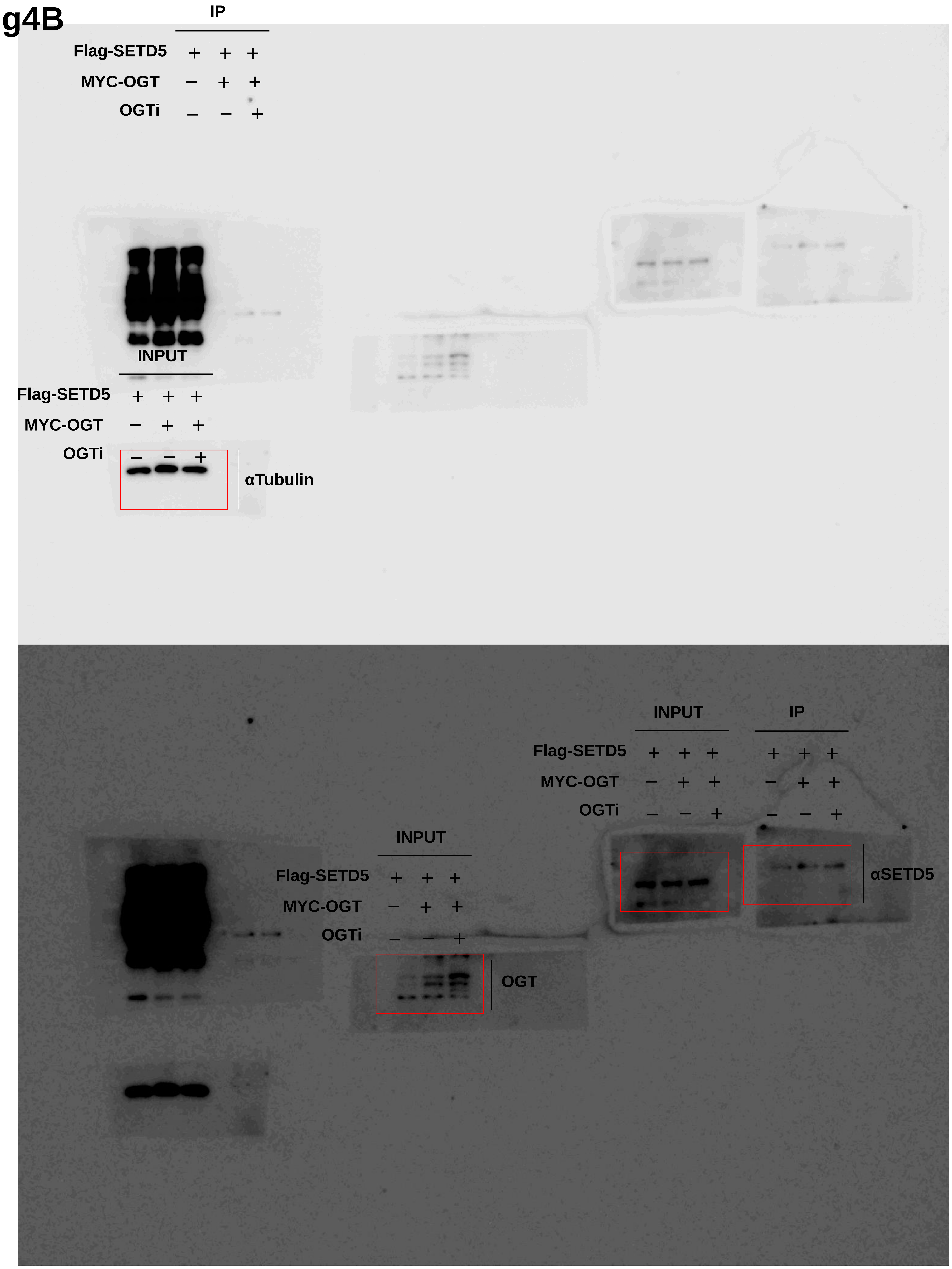
IP
Flag-SETD5 + + +
MYC-OGT − + +
OGTi − − +
INPUT
Flag-SETD5 + + +
MYC-OGT − + +
OGTi − − +
αTubulin
INPUT
Flag-SETD5 + + +
MYC-OGT − + +
OGTi − − +
OGT
INPUT
IP
Flag-SETD5 + + + + + +
MYC-OGT − + + − + +
OGTi − − + − − +
αSETD5

SFig4B

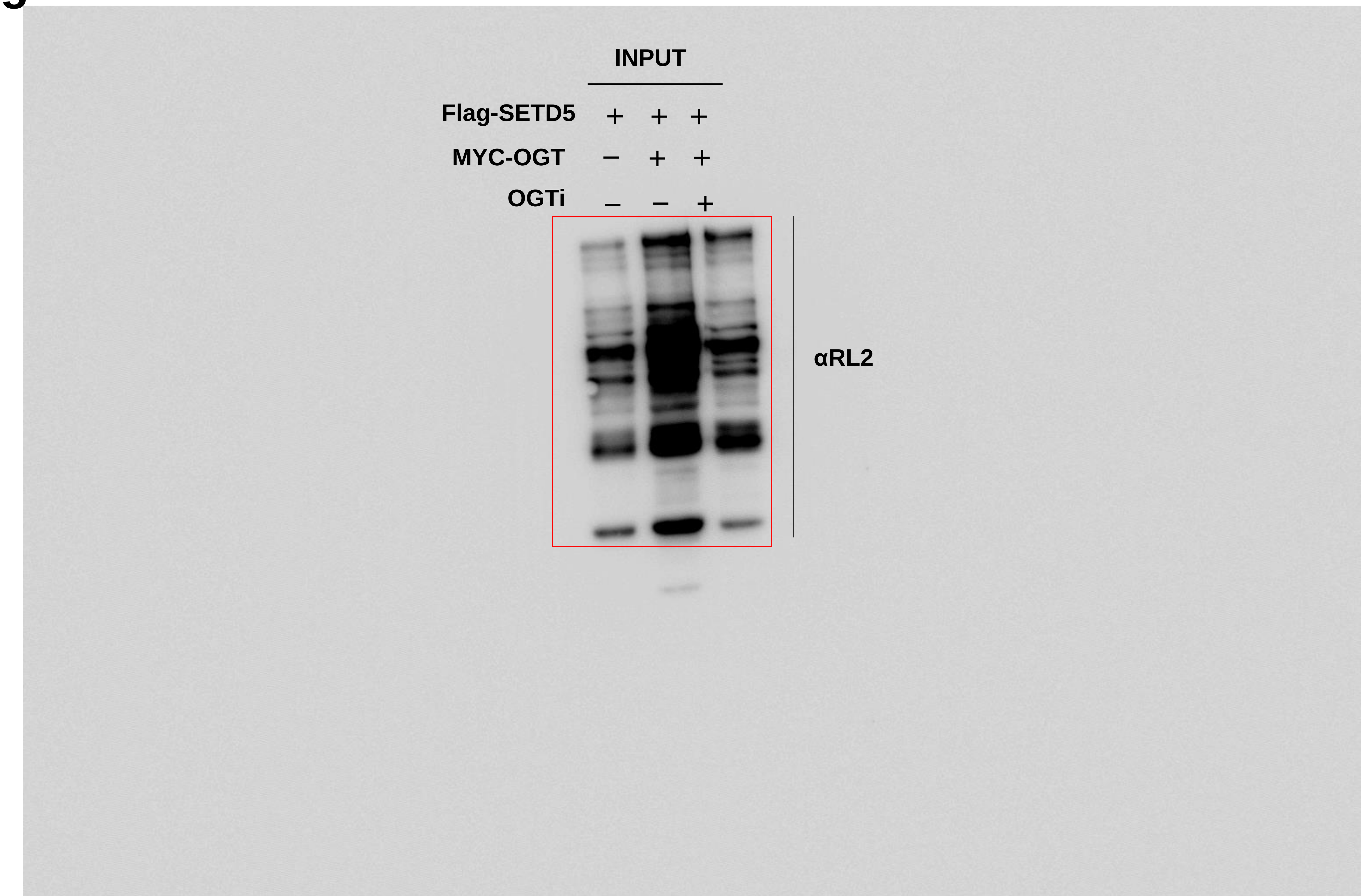
INPUT
Flag-SETD5 + + +
MYC-OGT − + +
OGTi − − +
αRL2

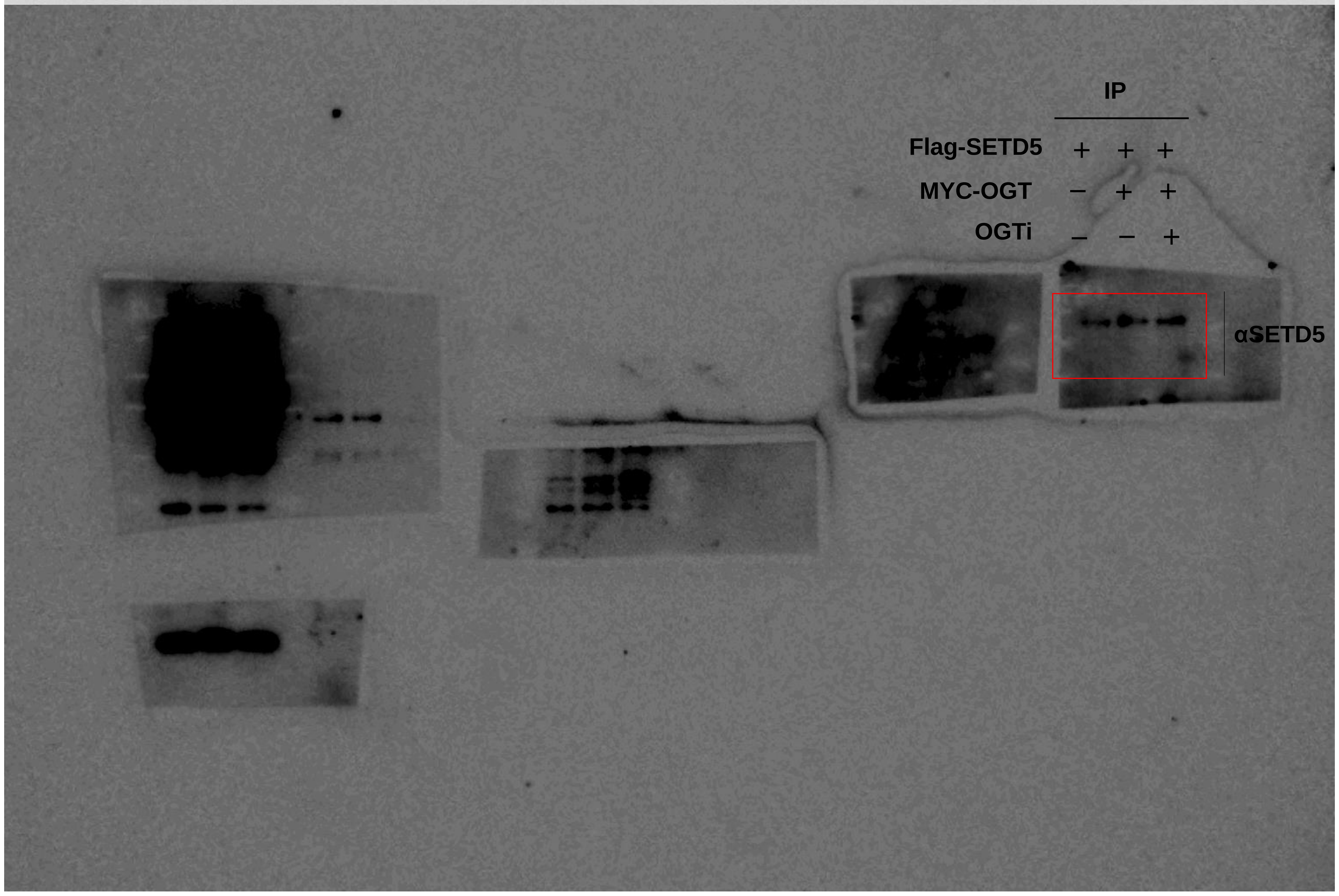
IP
Flag-SETD5 + + +
MYC-OGT − + +
OGTi − − +
αSETD5
